# Supplementary material for: Depression, depressive symptoms and treatments in women who have recently given birth: UK cohort study
Source: BMJ Open. 2018 Oct 24;8(10):e022152. doi: 10.1136/bmjopen-2018-022152 (PMC6224756; doi:10.1136/bmjopen-2018-022152)
Supplement: Supplementary file 1 [file bmjopen-2018-022152supp001.pdf]

STable 1. Rates and relative risk estimates of depression diagnosis, postnatal depression diagnosis and depression symptoms in the first year after delivery for 142,234 women who gave birth between 2000 and 2013 and had no prior record suggestive of depression. Adjusted by age group, calendar period, and Townsend deprivation index. RR = relative risk.

	Depression diagnosis		Postnatal depression diagnosis		Depression symptom	
	Rate per 100	Adjusted RR	Rate per 100	Adjusted RR	Rate per 100	Adjusted RR
Age group						
15-20	4.9 (4.4 to 5.3)	2.74 (2.40 to 3.13)	6.6 (6.0 to 7.1)	2.88 (2.56 to 3.23)	8.4 (7.8 to 9.0)	3.35 (3.01 to 3.72)
20-25	3.8 (3.5 to 4.0)	2.21 (1.97 to 2.47)	4.4 (4.1 to 4.7)	1.98 (1.80 to 2.17)	5.2 (4.9 to 5.6)	2.13 (1.95 to 2.33)
25-30	2.2 (2.0 to 2.4)	1.33 (1.21 to 1.47)	3.0 (2.8 to 3.2)	1.33 (1.22 to 1.45)	3.0 (2.8 to 3.1)	1.26 (1.16 to 1.37)
30-35	1.6 (1.5 to 1.7)	1	2.3 (2.1 to 2.4)	1	2.3 (2.1 to 2.4)	1
35-40	1.4 (1.3 to 1.6)	0.90 (0.81 to 1.01)	2.0 (1.8 to 2.2)	0.89 (0.80 to 0.98)	2.0 (1.9 to 2.2)	0.90 (0.82 to 0.99)
40-50	1.2 (0.9 to 1.5)	0.75 (0.59 to 0.95)	1.8 (1.5 to 2.1)	0.81 (0.67 to 0.99)	1.8 (1.5 to 2.1)	0.79 (0.64 to 0.96)
Calendar period						
2000-2004	3.0 (2.8 to 3.2)	1	3.9 (3.7 to 4.1)	1	2.6 (2.4 to 2.8)	1
2005-2009	2.1 (2.0 to 2.2)	0.67 (0.60 to 0.75)	2.9 (2.7 to 3.0)	0.70 (0.64 to 0.77)	3.4 (3.3 to 3.6)	1.27 (1.15 to 1.40)
2010-2013	1.7 (1.6 to 1.8)	0.53 (0.47 to 0.60)	2.1 (2.0 to 2.3)	0.52 (0.47 to 0.57)	3.1 (2.9 to 3.2)	1.13 (1.01 to 1.26)
Townsend deprivation index quantile						
1	1.6 (1.5 to 1.8)	1	2.4 (2.2 to 2.5)	1	2.3 (2.1 to 2.4)	1
2	1.9 (1.7 to 2.0)	1.11 (0.98 to 1.26)	2.6 (2.4 to 2.8)	1.06 (0.97 to 1.17)	2.6 (2.4 to 2.7)	1.05 (0.95 to 1.15)
3	2.1 (2.0 to 2.3)	1.19 (1.05 to 1.34)	3.0 (2.8 to 3.2)	1.17 (1.06 to 1.29)	3.1 (2.9 to 3.3)	1.18 (1.06 to 1.31)
4	2.6 (2.4 to 2.8)	1.34 (1.18 to 1.51)	3.3 (3.1 to 3.5)	1.16 (1.04 to 1.29)	3.8 (3.6 to 4.0)	1.28 (1.15 to 1.42)
5	3.1 (2.8 to 3.3)	1.42 (1.24 to 1.64)	3.5 (3.2 to 3.7)	1.10 (0.96 to 1.27)	4.4 (4.1 to 4.7)	1.32 (1.18 to 1.48)

STable 2. Rates and relative risk estimates of SSRI prescription and non-pharmacological treatment in the first year after delivery for 142,234 women who gave birth between 2000 and 2013 and had no prior record suggestive of depression. Adjusted by age group, calendar period, and Townsend deprivation index. RR = relative risk.

	SSRI prescription		Non-pharmacological treatment	
	Rate per 100	Adjusted RR	Rate per 100	Adjusted RR
Age group				
15-20	14.2 (13.4 to 15.0)	3.10 (2.85 to 3.37)	3.4 (3.0 to 3.8)	2.48 (2.12 to 2.90)
20-25	9.9 (9.4 to 10.3)	2.20 (2.06 to 2.36)	2.2 (2.0 to 2.4)	1.60 (1.40 to 1.84)
25-30	5.8 (5.5 to 6.0)	1.32 (1.24 to 1.41)	1.6 (1.5 to 1.8)	1.21 (1.08 to 1.36)
30-35	4.3 (4.1 to 4.5)	1	1.3 (1.2 to 1.4)	1
35-40	3.7 (3.5 to 3.9)	0.86 (0.80 to 0.93)	1.3 (1.1 to 1.4)	0.94 (0.83 to 1.07)
40-50	3.1 (2.7 to 3.5)	0.73 (0.63 to 0.84)	1.2 (0.9 to 1.4)	0.87 (0.69 to 1.11)
Calendar period				
2000-2004	6.5 (6.3 to 6.8)	1	1.2 (1.1 to 1.3)	1
2005-2009	5.8 (5.7 to 6.0)	0.86 (0.81 to 0.91)	1.8 (1.7 to 1.9)	1.42 (1.25 to 1.62)
2010-2013	5.1 (4.9 to 5.3)	0.75 (0.70 to 0.80)	1.7 (1.6 to 1.9)	1.40 (1.22 to 1.61)
Townsend deprivation index quantile				
1	4.5 (4.3 to 4.7)	1	1.4 (1.2 to 1.5)	1
2	4.9 (4.7 to 5.2)	1.05 (0.98 to 1.13)	1.5 (1.4 to 1.6)	1.06 (0.92 to 1.23)
3	5.9 (5.6 to 6.1)	1.15 (1.07 to 1.24)	1.6 (1.5 to 1.8)	1.08 (0.93 to 1.24)
4	6.7 (6.4 to 7.0)	1.18 (1.09 to 1.28)	1.8 (1.7 to 2.0)	1.12 (0.97 to 1.30)
5	8.0 (7.6 to 8.4)	1.26 (1.13 to 1.40)	2.0 (1.8 to 2.2)	1.12 (0.94 to 1.32)

STable 3. Rates and relative risk estimates of depression diagnosis, postnatal depression diagnosis and depression symptoms in the first year after delivery for 64,283 women who gave birth between 2000 and 2013 and had a prior record suggestive of depression. Adjusted by age group, calendar period, and Townsend deprivation index. RR = relative risk.

	Depression diagnosis		Postnatal depression diagnosis		Depression symptom	
	Rate per 100	Adjusted RR	Rate per 100	Adjusted RR	Rate per 100	Adjusted RR
Age group						
15-20	12.6 (11.3 to 14.0)	1.48 (1.31 to 1.67)	11.1 (9.8 to 12.4)	1.50 (1.32 to 1.71)	18.3 (16.7 to 19.9)	1.76 (1.58 to 1.95)
20-25	10.6 (10.0 to 11.2)	1.24 (1.15 to 1.35)	8.2 (7.7 to 8.7)	1.11 (1.02 to 1.21)	13.3 (12.7 to 14.0)	1.29 (1.22 to 1.38)
25-30	9.2 (8.7 to 9.6)	1.10 (1.03 to 1.17)	7.9 (7.5 to 8.3)	1.07 (0.99 to 1.15)	10.6 (10.1 to 11.1)	1.06 (0.99 to 1.12)
30-35	8.3 (7.9 to 8.7)	1	7.6 (7.2 to 8.0)	1	9.8 (9.3 to 10.2)	1
35-40	8.0 (7.6 to 8.5)	0.98 (0.91 to 1.06)	6.8 (6.3 to 7.2)	0.90 (0.82 to 0.97)	9.2 (8.7 to 9.7)	0.95 (0.88 to 1.01)
40-50	6.7 (5.8 to 7.5)	0.84 (0.73 to 0.97)	5.7 (4.9 to 6.5)	0.78 (0.67 to 0.89)	9.9 (8.9 to 11.0)	1.02 (0.91 to 1.14)
Calendar period						
2000-2004	12.7 (12.1 to 13.2)	1	10.8 (10.2 to 11.3)	1	9.1 (8.7 to 9.6)	1
2005-2009	8.8 (8.4 to 9.1)	0.68 (0.63 to 0.74)	7.7 (7.3 to 8.0)	0.71 (0.66 to 0.77)	11.7 (11.3 to 12.1)	1.26 (1.16 to 1.38)
2010-2013	7.0 (6.7 to 7.3)	0.54 (0.50 to 0.59)	5.9 (5.6 to 6.2)	0.54 (0.50 to 0.59)	10.6 (10.3 to 11.0)	1.14 (1.03 to 1.25)
Townsend deprivation index quantile						
1	7.6 (7.2 to 8.1)	1	7.6 (7.1 to 8.0)	1	9.2 (8.7 to 9.7)	1
2	8.1 (7.6 to 8.6)	1.06 (0.98 to 1.16)	7.8 (7.3 to 8.2)	1.04 (0.95 to 1.13)	9.5 (9.0 to 10.1)	1.01 (0.93 to 1.11)
3	8.5 (8.0 to 8.9)	1.10 (1.01 to 1.20)	7.1 (6.6 to 7.5)	0.93 (0.85 to 1.03)	10.0 (9.5 to 10.5)	1.04 (0.95 to 1.13)
4	9.7 (9.2 to 10.2)	1.25 (1.15 to 1.36)	7.7 (7.2 to 8.1)	1.00 (0.92 to 1.09)	11.9 (11.4 to 12.4)	1.21 (1.11 to 1.32)
5	10.6 (10.0 to 11.1)	1.33 (1.20 to 1.47)	8.2 (7.7 to 8.7)	1.05 (0.94 to 1.16)	13.0 (12.4 to 13.6)	1.29 (1.16 to 1.43)

STable 4. Rates and relative risk estimates of SSRI prescription and non-pharmacological treatment in the first year after delivery for 64,283 women who gave birth between 2000 and 2013 and had a prior record suggestive of depression. Adjusted by age group, calendar period, and Townsend deprivation index. RR = relative risk.

	SSRI prescription		Non-pharmacological treatment	
	Rate per 100	Adjusted RR	Rate per 100	Adjusted RR
Age group				
15-20	34.3 (32.4 to 36.3)	1.47 (1.38 to 1.57)	10.1 (8.8 to 11.3)	1.42 (1.25 to 1.62)
20-25	27.3 (26.4 to 28.1)	1.18 (1.13 to 1.23)	8.3 (7.8 to 8.9)	1.18 (1.09 to 1.29)
25-30	23.9 (23.2 to 24.5)	1.04 (1.01 to 1.08)	6.8 (6.4 to 7.2)	0.99 (0.91 to 1.06)
30-35	22.6 (22.0 to 23.2)	1	6.8 (6.4 to 7.1)	1
35-40	22.0 (21.2 to 22.7)	0.98 (0.94 to 1.02)	6.5 (6.0 to 6.9)	0.95 (0.87 to 1.04)
40-50	21.2 (19.8 to 22.6)	0.94 (0.88 to 1.01)	6.5 (5.7 to 7.4)	0.95 (0.83 to 1.09)
Calendar period				
2000-2004	24.0 (23.3 to 24.8)	1	5.4 (5.0 to 5.8)	1
2005-2009	23.6 (23.1 to 24.1)	0.97 (0.93 to 1.02)	7.3 (7.0 to 7.6)	1.34 (1.21 to 1.49)
2010-2013	24.1 (23.6 to 24.6)	0.99 (0.95 to 1.03)	7.8 (7.5 to 8.1)	1.43 (1.27 to 1.61)
Townsend deprivation index quantile				
1	21.7 (21.0 to 22.4)	1	6.5 (6.0 to 6.9)	1
2	22.7 (22.0 to 23.5)	1.04 (0.99 to 1.09)	6.8 (6.4 to 7.3)	1.04 (0.93 to 1.16)
3	23.2 (22.5 to 23.9)	1.04 (0.99 to 1.10)	6.9 (6.5 to 7.3)	1.02 (0.92 to 1.14)
4	25.0 (24.3 to 25.7)	1.11 (1.06 to 1.16)	7.5 (7.1 to 8.0)	1.10 (0.99 to 1.23)
5	26.6 (25.8 to 27.4)	1.16 (1.10 to 1.22)	7.6 (7.2 to 8.1)	1.10 (0.97 to 1.25)

STable 5. Rates of early recording (recording date within 42 days of delivery date) and odds ratio estimates of early vs. late recording (at or after 42 days) for depression diagnosis, postnatal depression diagnosis and depression symptoms in the first year after delivery for women who gave birth between 2000 and 2013. The values are calculated in datasets that only contain the women with the corresponding record within one year after delivery (number of women are given in the headers). Adjusted by age group, calendar period, Townsend deprivation index, and a prior record suggestive of depression. OR = odds ratio.

	Depression diagnosis (N=8,815)		Postnatal depression diagnosis (N=9,005)		Depression symptom (N=11,318)	
	Rate per 100	Adjusted OR	Rate per 100	Adjusted OR	Rate per 100	Adjusted OR
Age group						
15-20	8.6 (6.4 to 10.7)	1.23 (0.89 to 1.67)	23.3 (20.3 to 26.3)	1.43 (1.17 to 1.75)	12.5 (10.5 to 14.5)	1.23 (0.98 to 1.53)
20-25	8.1 (6.8 to 9.4)	1.00 (0.80 to 1.26)	19.2 (17.3 to 21.1)	1.04 (0.88 to 1.22)	12.9 (11.6 to 14.3)	1.19 (1.00 to 1.41)
25-30	8.6 (7.4 to 9.7)	1.00 (0.82 to 1.24)	17.6 (16.0 to 19.1)	0.91 (0.79 to 1.06)	10.8 (9.6 to 11.9)	0.96 (0.81 to 1.14)
30-35	8.5 (7.4 to 9.7)	1	18.7 (17.2 to 20.3)	1	10.9 (9.7 to 12.0)	1
35-40	9.8 (8.3 to 11.4)	1.14 (0.91 to 1.43)	18.2 (16.2 to 20.2)	0.95 (0.80 to 1.12)	10.4 (9.0 to 11.8)	0.95 (0.78 to 1.16)
40-50	10.0 (6.6 to 13.4)	1.12 (0.73 to 1.66)	19.5 (15.0 to 23.9)	1.03 (0.76 to 1.39)	12.5 (9.4 to 15.6)	1.08 (0.79 to 1.46)
Calendar period						
2000-2004	8.6 (7.5 to 9.6)	1	17.6 (16.2 to 19.0)	1	8.4 (7.2 to 9.5)	1
2005-2009	8.4 (7.4 to 9.3)	0.94 (0.79 to 1.13)	18.0 (16.7 to 19.2)	0.99 (0.87 to 1.13)	10.9 (10.1 to 11.8)	1.31 (1.10 to 1.57)
2010-2013	9.4 (8.3 to 10.5)	1.06 (0.87 to 1.28)	21.6 (20.0 to 23.2)	1.24 (1.08 to 1.42)	13.6 (12.6 to 14.6)	1.65 (1.38 to 1.97)
Townsend deprivation index quantile						
1	8.7 (7.2 to 10.1)	1	19.3 (17.5 to 21.1)	1	9.5 (8.2 to 10.8)	1
2	9.0 (7.6 to 10.5)	1.04 (0.81 to 1.34)	16.2 (14.5 to 18.0)	0.79 (0.66 to 0.94)	10.6 (9.2 to 12.0)	1.10 (0.89 to 1.36)
3	8.3 (7.0 to 9.6)	0.94 (0.74 to 1.21)	18.2 (16.5 to 19.9)	0.91 (0.77 to 1.07)	12.5 (11.2 to 13.8)	1.30 (1.07 to 1.59)
4	8.7 (7.5 to 9.9)	0.97 (0.77 to 1.24)	21.2 (19.4 to 23.0)	1.07 (0.91 to 1.26)	11.9 (10.7 to 13.1)	1.20 (0.99 to 1.46)
5	9.0 (7.7 to 10.3)	0.99 (0.78 to 1.27)	19.0 (17.1 to 20.9)	0.89 (0.75 to 1.06)	12.0 (10.7 to 13.3)	1.19 (0.98 to 1.46)
Any prior record suggestive of depression						
No	4.8 (4.0 to 5.5)	1	15.4 (14.2 to 16.5)	1	8.9 (8.0 to 9.7)	1
Yes	10.8 (10.0 to 11.6)	2.43 (2.02 to 2.94)	21.8 (20.6 to 22.9)	1.58 (1.41 to 1.77)	13.0 (12.2 to 13.8)	1.55 (1.37 to 1.76)

STable 6. Rates of early recording (recording date within 42 days of delivery date) and odds ratio estimates of early vs. late recording (at or after 42 days) for SSRI prescription and non-pharmacological treatment in the first year after delivery for women who gave birth between 2000 and 2013. The values are calculated in datasets that only contain the women with the corresponding record within one year after delivery (number of women are given in the headers). Adjusted by age group, calendar period, Townsend deprivation index, and a prior record suggestive of depression. OR = odds ratio.

	SSRI prescription (N=23,557)		Non-pharmacological treatment (N=6,848)	
	Rate per 100	Adjusted OR	Rate per 100	Adjusted OR
Age group				
15-20	17.4 (15.7 to 19.1)	0.99 (0.86 to 1.14)	12.5 (9.6 to 15.4)	1.01 (0.74 to 1.36)
20-25	17.6 (16.5 to 18.7)	0.83 (0.75 to 0.92)	11.7 (9.9 to 13.4)	0.82 (0.66 to 1.03)
25-30	18.6 (17.7 to 19.6)	0.85 (0.77 to 0.93)	13.4 (11.7 to 15.0)	0.96 (0.79 to 1.17)
30-35	21.0 (20.0 to 22.0)	1	13.8 (12.2 to 15.3)	1
35-40	21.0 (19.7 to 22.3)	0.96 (0.87 to 1.06)	14.3 (12.3 to 16.3)	1.03 (0.84 to 1.27)
40-50	24.3 (21.5 to 27.1)	1.07 (0.90 to 1.26)	16.0 (11.8 to 20.2)	1.13 (0.79 to 1.57)
Calendar period				
2000-2004	15.2 (14.2 to 16.1)	1	12.0 (10.1 to 13.8)	1
2005-2009	18.4 (17.6 to 19.2)	1.20 (1.09 to 1.31)	11.4 (10.3 to 12.6)	0.95 (0.77 to 1.17)
2010-2013	23.7 (22.8 to 24.6)	1.59 (1.46 to 1.74)	16.0 (14.6 to 17.4)	1.36 (1.11 to 1.68)
Townsend deprivation index quantile				
1	18.7 (17.6 to 19.9)	1	13.3 (11.5 to 15.2)	1
2	19.1 (17.9 to 20.3)	1.00 (0.90 to 1.12)	12.8 (10.9 to 14.6)	0.94 (0.74 to 1.19)
3	19.3 (18.2 to 20.4)	1.02 (0.92 to 1.14)	14.0 (12.2 to 15.8)	1.05 (0.84 to 1.31)
4	20.4 (19.4 to 21.5)	1.09 (0.98 to 1.21)	13.4 (11.7 to 15.1)	0.98 (0.79 to 1.23)
5	20.0 (18.9 to 21.2)	1.06 (0.95 to 1.18)	13.3 (11.4 to 15.1)	0.97 (0.77 to 1.23)
Any prior record suggestive of depression				
No	9.6 (8.9 to 10.2)	1	8.8 (7.6 to 9.9)	1
Yes	24.9 (24.2 to 25.6)	3.02 (2.78 to 3.29)	15.7 (14.7 to 16.8)	1.91 (1.62 to 2.27)
