# Supplementary material for: Depression, depressive symptoms and treatments in women who have recently given birth: UK cohort study
Source: BMJ Open. 2018 Oct 24;8(10):e022152. doi: 10.1136/bmjopen-2018-022152 (PMC6224756; doi:10.1136/bmjopen-2018-022152)
Supplement: Author's manuscript [file bmjopen-2018-022152.draft_revisions.pdf]

BMJ Open is committed to open peer review. As part of this commitment we make the peer review history of every article we publish publicly available.

When an article is published we post the peer reviewers' comments and the authors' responses online. We also post the versions of the paper that were used during peer review. These are the versions that the peer review comments apply to.

The versions of the paper that follow are the versions that were submitted during the peer review process. They are not the versions of record or the final published versions. They should not be cited or distributed as the published version of this manuscript.

BMJ Open is an open access journal and the full, final, typeset and author-corrected version of record of the manuscript is available on our site with no access controls, subscription charges or pay-per-view fees (http://bmjopen.bmj.com).

If you have any questions on BMJ Open's open peer review process please email info.bmjopen@bmj.com

BMJ Open

# Depression, depressive symptoms and treatments in women who have recently given birth: UK cohort study



| | |
|---|---|
| Journal: | *BMJ Open* |
| Manuscript ID | bmjopen-2018-022152 |
| Article Type: | Research |
| Date Submitted by the Author: | 06-Feb-2018 |



| | |
|---|---|
| Complete List of Authors: | Petersen, Irene; University College London Research Department of Primary Care and Population Health<br>Peltola, Tomi; Aalto University, Helsinki Institute for Information Technology HIIT<br>Kaski, Samuel; Aalto University, Helsinki Institute for Information Technology HIIT<br>Walters, Kate; University College London Research Department of Primary Care and Population Health<br>Hardoon, Sarah; University College London Research Department of Primary Care and Population Health |



| | |
|---|---|
| Keywords: | postnatal depression, SSRI treatment, non-pharmacological treatment, PRIMARY CARE, Depression & mood disorders < PSYCHIATRY |


SCHOLARONE™ Manuscripts

For peer review only

# Depression, depressive symptoms and treatments in women who have recently given birth: UK cohort study

Irene Petersen, PhD (1, 2), Tomi Peltola, PhD (1,3), Samuel Kaski, PhD (3), Kate Walters, PhD (1) and Sarah Hardoon, PhD (1)

Affiliations:

1) Department of Primary Care and Population Health, University College London, Rowland Hill St, London NW3 2PF, UK

2) Department of Clinical Epidemiology, Aarhus University, Olof Palmes Alle 43 – 45, 8200 Aarhus N, Denmark

3) Helsinki Institute for Information Technology HIIT, Department of Computer Science, Aalto University, Konemiehentie 2, 02150 Espoo, Finland

Corresponding author: Irene Petersen, e-mail: i.petersen@ucl.ac.uk, Department of Primary Care and Population Health, UCL, Rowland Hill St, London NW3 2PF, UK.

Word count: 3387

For peer review only

## Abstract

Objectives: To investigate how depression is recognised in the year after child birth and treatment given in clinical practice.

Design: Cohort study

Setting: Primary Care

Participants: Women who have given live birth between 2000 and 2013.

Outcomes: Prevalence of postnatal depression, depression, depressive symptoms, antidepressant and non-pharmacological treatment within a year after birth.

Results: Of 206,517 women 23,623 (11%) had a record of depressive diagnosis or symptoms in the year after delivery and more than 1 in 8 women received antidepressant treatment. Recording and treatment peaked 6 to 8 weeks after delivery. Initiation of SSRI treatment has become earlier in the more recent years. Thus, the initiation rate of SSRI treatment per 100 pregnancies (95% CI) at 8 weeks were 2.6 (2.5 to 2.8) in 2000-2004, increasing to 3.0 (2.9 to 3.1) in 2005-2009, and 3.8 (3.6 to 3.9) in 2010-2013. The overall rate of initiation of SSRI within the year after delivery, however, has not changed noticeably. A third of the women had at least one record suggestive of depression at any time prior to delivery and of these 1 in 4 received SSRI treatment in the year after delivery.

Younger women were most likely to have records of depression and depressive symptoms. Relative Risk for postnatal depression: Age 15 – 19: 1.92 (1.76 to 2.10), Age 20 – 24: 1.49 (1.39 to 1.59) versus Age 30 – 34)). The risk of depression, postnatal depression and depressive symptoms increased with increasing social deprivation.

Conclusions: More than 1 in 10 women had electronic health records indicating depression or depressive symptoms within a year after delivery and more than 1 in 8 women received antidepressant treatment in this period. Women aged below 30 and from the most deprived areas were at highest risk depression and most likely to receive antidepressant treatment.


## Summary

Strength and limitations of this study



For peer review only - http://bmjopen.bmj.com/site/about/guidelines.xhtml

- A major strength of this study is that we have access to a very large sample of primary care electronic health records of women who gave live birth.
- These records reflect clinical practice in UK primary care and were made prospectively.
- We considered a broad definition of depression on clinical evaluation in the year after delivery as there are no specific guidelines to how it should be recorded.
- This study may overestimate the number of women with postnatal depression compared to estimates based on a diagnostic interview and specific diagnostic instruments.
- Non-pharmacological treatment may not be well recorded in primary care electronic health records.

For peer review only



For peer review only - http://bmjopen.bmj.com/site/about/guidelines.xhtml

## Introduction

Many women experience depression in the year after they have given birth. Postnatal depression affects an estimated 10 – 19% of women, although the estimates vary substantially between countries and settings. (1–3) Depression may have severe consequences for the mother and, in turn, have physical, cognitive, and emotional effects on their children's development, continuing into later life. (4–7) A report published by the London School of Economics estimated that perinatal depression, anxiety and psychosis carry a total long-term cost to society of about £6.6 billion for each one-year cohort of births in the UK. (4) This is equivalent to a cost of just under £10,000 for every single birth in the country. Nearly three-quarters (72%) of this cost relates to adverse impacts on the child rather than the mother. (4)

Guidelines in both US and UK on antenatal and postnatal mental health recommend that health care professionals should consider asking simple screening questions about current and past histories of depression, anxiety, alcohol and illicit drug use as part of a general discussion about mental health and wellbeing in pregnancy and the perinatal period. (8,9) However, very limited information is available on when depression is recognised and how it is treated in clinical practice in the year after women have given birth. For most women who experience depression in this period primary care physicians would be a first point of contact. In this study, we sought to obtain an overview of actual clinical practice in UK primary care by examining electronic health records on more than 200,000 women who have given life birth between 2000 and 2013. We followed the women for a year after delivery and our aim was to examine how and when depression and depressive symptoms were recorded and treatment provided in general practice and the interrelation between antidepressant and non-pharmacological treatment.

For peer review only



For peer review only - http://bmjopen.bmj.com/site/about/guidelines.xhtml

# Methods

## Data source

We used data from The Health Improvement Network (THIN). This is a large primary care database that provides anonymised longitudinal general practice (family practice) data on patients' clinical and prescribing records and includes data from around 6% of the United Kingdom population. Diagnoses and symptoms are recorded by practice staff using Read codes, which is a hierarchical coding system including more than 100,000 codes. (10,11) The Read code system can be mapped to ICD-10, but in addition the Read codes include a number of symptoms and administrative codes. (11) Prescriptions are issued electronically and directly recorded on the general practice computer systems. In addition, the database holds individual patient level information about year of birth, date of registration, date of death and transfer out of the practice and information about social deprivation (quintiles of Townsend deprivation scores) (12) is linked by census data.

Over 98% of the UK population are registered with a general practitioner (GP) (13) and the UK primary care databases are broadly representative of the United Kingdom population. (14,15) While perinatal care is often shared between general practice staff and midwives, the GP remains responsible for women's general medical care including continued prescribing of medicines such as antidepressants. Some women may also receive care from local National Health Service (NHS) mental health trusts, but trusts have limited prescribing budgets and for most women prescribing of psychotropic medication remains with the GP. Furthermore, after a few weeks after delivery the care by the midwife ends and general practitioners are the first point of contact. Typically, women will consult their general practitioner for a postnatal maternal check-up at 6 to 8 weeks after delivery.

## Study population

We utilised data from women who have given live birth between 1st January 2000 and 31st December 2013 and who were permanently registered with the same general practice for at least one year after delivery. As some women had more than one pregnancy and the risk of postnatal depression may be strongly correlated within women we randomly selected one pregnancy per woman for our analyses.

## Variables

We identified women with one or more records entered as a Read code in their primary care electronic health records which suggested they had depression, postnatal depression or symptoms of depression as well women on antidepressant and non-pharmacological treatment (referral to counselling and



For peer review only - http://bmjopen.bmj.com/site/about/guidelines.xhtml

psychotherapy) in the year after they have given birth. Antidepressant treatment was classified as selective serotonin reuptake inhibitors (SSRI), TCA and other antidepressants. For TCA we only considered treatment that was prescribed above treatment threshold for depression, as lower doses may be prescribed for other reasons such as chronic pain. In addition, we included information on calendar year of delivery, age at delivery and social deprivation.

## Data analysis

First, we estimated the prevalence of any records suggestive of depression (postnatal depression, depression diagnoses, depressive symptoms) as well as separate estimates for postnatal depression, depression diagnoses, depressive symptoms, antidepressant or non-pharmacological treatments within a year after giving birth. We then estimated how the records were interrelated. Interrelations were reported as conditional frequencies, that is, the frequency of having a record of X given that one has a record of Y. For example, having a prescription of a SSRI given one has a record of depression.

We estimated the timing of the recording within the follow-up year and report cumulative incidence curves (as one minus the Kaplan-Meier estimate). We also estimated smoothed daily hazards using a Gaussian process model (16) to visualize the daily changes in the timing of recording.

For each of the three depression outcomes; depression diagnoses, postnatal depression and depression symptoms we used Poisson regression to model relative risks of having a record associated with age, calendar time and social deprivation (Townsend scores). Age was split into six age groups (15-19, 20-24, 25-29, 30-34, 35-39, 40-49) and calendar time into three periods (2000-2004, 2005-2009, 2010-2013). 95% CI were computed using modified Poisson regression accounting for the clustering of women in general practices. We conducted supportive analyses stratified on whether women had records of depression or postnatal depression or SSRI treatment prior to delivery.

## Ethics

The scheme for THIN to obtain and provide anonymous patient data to researchers was approved by the National Health Service South-East Multicenter Research Ethics Committee (MREC) in 2002 and scientific approval for this study was obtained from IMS Scientific Review Committee.

## Patient and Public Involvement

Charlotte Walker, who is a mental health service user, has been involved with the original design of the study proposal and provided feedback on this manuscript and thus helped to shape the discussion of the paper from a service user's perspective.



For peer review only - http://bmjopen.bmj.com/site/about/guidelines.xhtml

## Results

In total, 206,517 women were included in the study and there were 23,623 (11%) with at least one record suggestive of depression (depression, postnatal depression or symptoms of depression) in the year after delivery. Of these women, there were 4% with a record of depression, 4% with a record of postnatal depression and 5% with symptoms of depression (Figure 1A). Of those women with a depression diagnosis, 2,349/8,815 (27%) also had depressive symptoms (Figure 1B), and of those with postnatal depression diagnosis, 2,005/9,005(22%) also had depressive symptoms (Figure 1B). In contrast, there were 7,408/11,318 (65%) women with a record of depressive symptoms *without* either a depression diagnosis or postnatal depression diagnosis.

The number of women with a record suggestive of depression continued to rise throughout the first year after delivery (Figure 2). However, the recording of postnatal depression levelled off after the first 3-4 months (Figure 2A). For all types of records, there were some clear peaks in recording immediately after delivery and in the period between 6 to 8 weeks after delivery coinciding with the time of postnatal maternal check-up consultation (Figure 2A).

There were 25,691 (12%) women with a record of antidepressant treatment. Women were predominantly prescribed SSRI (23,557 (92%)) with TCA (1,857 (7%)) and other (2,290 (8%)) prescriptions being much less common. Of the women who had an SSRI prescription, there were 31% who had a record of depression (Figure 1B), 31% who had a record of postnatal depression (Figure 1B), and 33% who had depression symptoms (Figure 1B) leaving 6,270 (27%) women with SSRI prescription *without* a record suggestive of depression within a year after delivery.

There were 6,848 (3%) women with a record of referral for non-pharmacological treatment (Figure 1A). Of the women receiving non-pharmacological treatment, there were 24% who had a record of depression (Figure 1B), 22% who had a record of postnatal depression (Figure 1B), and 29% who had depression symptoms (Figure 2B), but 3,064 (45%) with no records indicating depression, postnatal depression or depressive symptoms. Of those with non-pharmacological treatment referral, 56% had SSRI prescription (Figure 1B), whereas conversely only 16% with a SSRI prescription had a record of non-pharmacological treatment referral (Figure 1B).

After the initial peak, the hazard for recording of postnatal depression and SSRI prescription show a markedly decreasing trend, while the other records show a relatively stable rate or slower decline (Figure 2).



For peer review only - http://bmjopen.bmj.com/site/about/guidelines.xhtml

There were 64,283 (31%) women who had at least one record suggestive of depression at any time prior to delivery. The prevalence of depression and SSRI treatment *after* delivery was high among these women. Thus, there were 9,666 (15%) with a record of depression or postnatal depression and 15,348 (24%) received SSRI treatment in the year after delivery. The figures were similar for women who have received SSRI treatment (n = 40,178, 19%) at any time prior delivery. Thus, there were 6,940 (17%) with a record of depression or postnatal depression and 11,595 (29%) received SSRI treatment in the year after delivery.

## Age, social deprivation and time

Younger women were much more likely to have a record of depressive diagnoses or symptoms compared to women aged 30 years or older. For example, women aged 15 – 19 years were nearly twice as likely to have a record of postnatal depression (RR, adjusted for social deprivation: 1.92 (1.76 to 2.10)) compared to women aged 30 – 34 years (Table 1). There were no marked differences for women above the age of 30 (Table 1). The pattern of SSRI treatment followed the same trends with nearly 1 in 5 women aged 15 – 19 receiving SSRI treatment in the first year after delivery (Table 2) while for those aged above 30 it was 1 in 10 (Table 2). Younger women were also more likely to receive non-pharmacological treatment than women aged 30 years or above (Table 2).

The time to the initiation of SSRI treatment after the delivery has become earlier in the more recent years (Figure 3). Thus, the initiation rate of SSRI treatment per 100 pregnancies (95% CI) at 8 weeks were 2.6 (2.5 to 2.8) in 2000-2004, increasing to 3.0 (2.9 to 3.1) in 2005-2009, and 3.8 (3.6 to 3.9) in 2010-2013. The overall rate of initiation of SSRI within the year after delivery, however, has not changed noticeably (Table 2). The rates of non-pharmacological treatment have increased from 2.4 (2.2 to 2.5) per 100 pregnancies in 2000-2004 to 3.8 (3.6 to 3.9) in 2010 – 2013 (Table 2). The recording of both depression diagnosis and postnatal diagnosis has decreased substantially over time while the recording of symptoms increased in the earlier time period, but have remained relatively constant since 2005 (Table 1).

The risk of having a record of depression, postnatal depression and depressive symptoms increased with increasing social deprivation (Table 1) and similar patterns were observed for both SSRI treatment and non-pharmacological treatment (Table 2). Thus, nearly 1 in 7 women from the most deprived areas received SSRI treatment within the first year after delivery in contrast to 1 in 11 women from the least deprived areas (Table 2). Supportive analyses suggest that the effect of age is, in general, stronger among the women *without* records suggestive of depression prior to delivery than among women *with*



For peer review only - http://bmjopen.bmj.com/site/about/guidelines.xhtml

prior records (Appendix 1). However, the effect of social deprivation and calendar time was similar in women with and without prior records of depression (Appendix 1).

# Discussion

We found that 11% of women who have given live birth had a record suggestive of depression in their primary care electronic health records within the first year after delivery. There were some peaks in recording of depressive diagnoses and symptoms and initiation of SSRI treatment soon after delivery (6 to 8 weeks), coinciding with the time of postnatal maternal check-up consultations although they continued to be recorded throughout the first year after delivery. The time to the initiation of SSRI treatment after the delivery has become earlier in the more recent years although the overall rate of initiation of SSRI within the year after delivery has not changed. Women with records suggestive of depression or SSRI treatment prior to delivery were more likely to have a subsequent records and/or treatment after delivery.

Younger women were more likely to have a record suggestive of depression compared to women aged 30 years or older and the pattern of SSRI initiation followed the same trend with nearly 1 in 5 women aged between 15 – 19 years receiving SSRI treatment in the first year after delivery. The risk of depression increased with increasing social deprivation and similar patterns were observed for both SSRI treatment and non-pharmacological treatment.

## Strengths and limitations

A major strength of this study is that we have access to a very large sample of primary care electronic health records of women who gave live birth. These records reflect clinical practice in UK primary care and were made prospectively and therefore are not subject to recall bias. We considered a broad definition of depression based on clinical evaluation in the year after delivery as there are no specific guidelines to how it should be recorded in this period in primary care. Thus, we included women who had a specific diagnosis of postnatal depression as well women with records of depression diagnosis and symptoms, which may overestimate the number of women with postnatal depression compared to estimates based on a diagnostic interview and specific diagnostic instruments.

We are also aware that the indications for SSRI prescribing are broader than depression and some women in our study may have received SSRI for treatment for other indications for example anxiety. Yet, there is often an overlap between depression and anxiety and we chose therefore to include initiation of all SSRI prescriptions in our study. Our estimates of referral for non-pharmacological treatment were



For peer review only - http://bmjopen.bmj.com/site/about/guidelines.xhtml

relatively low. This may reflect a limited accessibility to non-pharmacological treatment, but it is also important to be aware that often in clinical practice the booking system for referrals is not directly linked to electronic health records and general practice staff will need to enter these referrals separately in the patient records. Furthermore, it is increasingly possible for women to self-refer themselves to psychological therapies through the 'Improving Access to Psychological Therapies' (IAPT) scheme in the UK (https://www.england.nhs.uk/mentalhealth/adults/iapt/). Therefore, it is likely that our study underestimates the actual referral rates for non-pharmacological treatments.

## Comparisons to existing evidence

Our summary estimate of postnatal depression, depression and symptoms of depression in the year after delivery (11%) was within the lower end of the range of previous prevalence estimates (10 – 19%). (2,3) Gavin et al estimated point prevalence of minor and major depression was highest in the third month after delivery at 12.9%, although the confidence intervals were wide. (2) The results of our study suggest a peak in depression records and antidepressant treatment within 6 to 8 weeks after delivery, coinciding with the time of postnatal check-up consultations.

Our findings of increase in the use of symptoms codes as opposed to diagnostic codes for recording of depression reflect previous findings on recording of depression in primary care in general. (17) Rait et al suggest general practitioners' coding may be linked to the perceived severity of depression, with symptom codes being used for milder depression. Alternatively, this move towards recording of symptoms and less specific terms may be perceived as less stigmatising for individuals. (17)

Nearly 1 out 5 women in our study had a record suggestive of depression and/or SSRI treatment records prior to delivery. Of these women, 17% had additional records of depression and more than a quarter received SSRI treatment in the year after delivery. Prior depression has long been recognised as one of the strongest risk factors for depression in the year after delivery. (1–3,18) Many women discontinue antidepressant treatment in pregnancy (19,20). A few studies suggest that these women are at higher risk of relapse (21) , but it is difficult to judge in observational settings and further research is needed to understand the role of antidepressant treatment in prevention of depression in the year after delivery.

Increased risk of postnatal depression among teenage mothers is well recognised with prevalence estimates as high as 26%. (22) However, our study demonstrated that the level of recording of depressive diagnoses and symptoms continued to be higher for women right up to the age of 30, whereas no marked difference was found for women above the age of 30. Previous meta-analyses of



For peer review only - http://bmjopen.bmj.com/site/about/guidelines.xhtml

For peer review only

postnatal depression have failed to recognise this ‘L-shaped’ difference in risk postnatal depression with age. (1,3)

There is some evidence that socioeconomic status is associated with prevalence of postnatal depression. (2,3,7,23) The results of a meta-regression analysis suggest that the prevalence of major depression is similar among socioeconomic status groups, but that minor depression may be more prevalent among lower socioeconomic status groups. (2) While we were unable to distinguish directly between diagnosis of major and minor depression we observed a clear gradient with increasing level of deprivation across all measures of depression and treatments. An even stronger socio-economic gradient in SSRI treatment was found among general population of adult women in UK. Hence, women from the most deprived areas were 64% more likely to have been initiated on SSRI treatment compared to women from the least deprived areas. (24)

Our study reflects women’s primary care electronic health records. For women to have records of depression it requires that they have consulted their general practitioner. However, some women may be reluctant to seek help and unwilling to disclose or discuss their problem because of fear of stigma, negative perceptions of them as a mother or fear that their baby might be taken into care. (5,25,26) Investigators and clinicians should also be aware of the potential differences in the way women express postpartum depression and that it may differ for women of different educational backgrounds.(27) Likewise, some healthcare professionals may miss or misdiagnose postnatal depression in the period soon after birth (5) and estimates based on primary care health records may underestimate the ‘true’ prevalence of postnatal depression. Our study clearly shows that for many women depression and depressive symptoms were ‘picked up’ and treatment initiated at the time of the maternal check-up consultation in accordance to guidelines on antenatal and postnatal mental health care. (26) Yet, our results also revealed that depression is not limited to the immediate period after delivery and emphasises the need for health care professionals to be alert to signs and symptoms of depression throughout the first year after delivery. Indeed, a recent systematic review suggested that screening postpartum women for depression may reduce depressive symptoms in women with depression and reduce the prevalence of depression. (28)

## Conclusions

More than 1 in 10 women had electronic health records indicating depression or depressive symptoms within a year after delivery and more than 1 in 8 women received antidepressant treatment in this



For peer review only - http://bmjopen.bmj.com/site/about/guidelines.xhtml

period. Women aged below 30 and from the most deprived areas were at highest risk depression and most likely to receive antidepressant treatment.

## Author contributions

IP, TP, SH, KW and SK conceived the study, TP conducted the statistical analyses together with IP. IP and TP drafted the manuscript. All authors contributed to preparing the manuscript and have agreed to submit the final version of the manuscript. IP is the guarantor.

## Funding for this study

SH, IP and KW received funding from National Institute for Health Research (NIHR) school of primary care (grant 325). The views expressed in this publication are those of the author(s) and not necessarily those of the NHS, the National Institute for Health Research or the Department of Health. TP was funded by Academy of Finland (Finnish Centre of Excellence in Computational Inference Research Grant number 284642).

## Competing interest

None of the authors had competing interests.

## Data sharing

As the data for this study was bought under a licence no data are available for data sharing.

For peer review only

Figure legends

Figure 1. A. Numbers of records of depressive diagnoses and symptoms as well as treatment. B. Conditional frequency of records: given that one has the condition on the y-axis, what is the frequency of having the condition on x-axis. D=Depression diagnosis, PND=Postnatal depression diagnosis, D/PND=either or both, D sym=Depression symptom, SSRI=SSRI prescription, NPT=Non-pharmacological treatment.

Figure 2. Cumulative incidences and smoothed hazards for the records. Six and eight weeks (6x7 and 8x7 days) are marked with a vertical grey line. Note the different y-axis scale for panels A and B. D=Depression, PND=Postnatal depression, SSRI=SSRI prescription, NPT=Non-pharmacological treatment.

Figure 3. Cumulative incidence of SSRI in three calendar periods. Six and eight weeks (6x7 and 8x7 days) are marked with a vertical grey line.

For peer review only



For peer review only - http://bmjopen.bmj.com/site/about/guidelines.xhtml

Table 1. Rates and Relative risk estimates of depression diagnosis, postnatal depression diagnosis and depression symptoms in the first year after delivery for 206,517 women who gave birth between 2000 and 2013. Adjusted by age group, calendar period, and Townsend deprivation

| | Depression diagnosis | | Postnatal depression diagnosis | | Depression symptom | |
|---|---|---|---|---|---|---|
| | Rate per 100 | Adjusted RR | Rate per 100 | Adjusted RR | Rate per 100 | Adjusted RR |
| Age group | | | | | | |
| 15-19 | 6.6 (6.1 to 7.1) | 1.64 (1.50 to 1.81) | 7.6 (7.1 to 8.1) | 1.92 (1.76 to 2.10) | 10.6 (10.0 to 11.2) | 2.10 (1.95 to 2.27) |
| 20-24 | 6.1 (5.9 to 6.4) | 1.59 (1.47 to 1.71) | 5.8 (5.5 to 6.0) | 1.49 (1.39 to 1.59) | 8.1 (7.7 to 8.4) | 1.63 (1.54 to 1.73) |
| 25-29 | 4.5 (4.3 to 4.7) | 1.22 (1.15 to 1.30) | 4.6 (4.4 to 4.8) | 1.21 (1.14 to 1.29) | 5.5 (5.3 to 5.7) | 1.18 (1.12 to 1.24) |
| 30-34 | 3.6 (3.4 to 3.7) | 1 | 3.8 (3.6 to 3.9) | 1 | 4.4 (4.3 to 4.6) | 1 |
| 35-39 | 3.5 (3.3 to 3.7) | 1.00 (0.93 to 1.06) | 3.5 (3.3 to 3.6) | 0.92 (0.86 to 0.98) | 4.3 (4.1 to 4.4) | 0.97 (0.92 to 1.02) |
| 40-49 | 3.1 (2.8 to 3.5) | 0.92 (0.81 to 1.03) | 3.2 (2.8 to 3.5) | 0.86 (0.77 to 0.97) | 4.7 (4.3 to 5.1) | 1.06 (0.96 to 1.17) |
| Calendar period | | | | | | |
| 2000-2004 | 5.7 (5.5 to 5.9) | 1 | 5.8 (5.6 to 6.0) | 1 | 4.4 (4.2 to 4.6) | 1 |
| 2005-2009 | 4.2 (4.0 to 4.3) | 0.71 (0.66 to 0.77) | 4.4 (4.2 to 4.5) | 0.73 (0.69 to 0.78) | 6.0 (5.8 to 6.2) | 1.31 (1.21 to 1.42) |
| 2010-2013 | 3.5 (3.3 to 3.6) | 0.58 (0.53 to 0.63) | 3.4 (3.3 to 3.5) | 0.56 (0.52 to 0.60) | 5.6 (5.4 to 5.8) | 1.21 (1.11 to 1.32) |
| Townsend deprivation index quintile | | | | | | |
| 1 | 3.2 (3.0 to 3.3) | 1 | 3.7 (3.5 to 3.9) | 1 | 4.1 (3.9 to 4.2) | 1 |
| 2 | 3.6 (3.4 to 3.8) | 1.14 (1.05 to 1.22) | 4.1 (3.9 to 4.2) | 1.09 (1.01 to 1.17) | 4.5 (4.3 to 4.7) | 1.07 (1.00 to 1.15) |
| 3 | 4.1 (3.9 to 4.3) | 1.26 (1.16 to 1.36) | 4.3 (4.1 to 4.5) | 1.12 (1.04 to 1.21) | 5.3 (5.1 to 5.5) | 1.19 (1.10 to 1.28) |
| 4 | 5.1 (4.9 to 5.3) | 1.51 (1.38 to 1.64) | 4.8 (4.6 to 5.0) | 1.20 (1.11 to 1.30) | 6.6 (6.4 to 6.9) | 1.42 (1.31 to 1.53) |
| 5 | 6.0 (5.7 to 6.3) | 1.69 (1.53 to 1.87) | 5.3 (5.0 to 5.5) | 1.26 (1.13 to 1.39) | 7.7 (7.4 to 8.0) | 1.56 (1.42 to 1.72) |

index. RR = relative risk.

For peer review only



For peer review only - http://bmjopen.bmj.com/site/about/guidelines.xhtml

Table 2. Rates and relative risk estimates of SSRI prescription and non-pharmacological treatment in the first year after delivery for 206,517 women who gave birth between 2000 and 2013. Adjusted by age group, calendar period, and Townsend deprivation index. RR = relative risk.

| | SSRI prescription | | Non-pharmacological treatment | |
|---|---|---|---|---|
| | Rate per 100 | Adjusted RR | Rate per 100 | Adjusted RR |
| Age group | | | | |
| 15-19 | 18.8 (18.0 to 19.5) | 1.78 (1.68 to 1.88) | 4.9 (4.5 to 5.4) | 1.55 (1.41 to 1.72) |
| 20-24 | 15.9 (15.5 to 16.4) | 1.54 (1.47 to 1.61) | 4.4 (4.1 to 4.6) | 1.38 (1.28 to 1.49) |
| 25-29 | 11.7 (11.5 to 12.0) | 1.18 (1.14 to 1.23) | 3.3 (3.2 to 3.5) | 1.11 (1.04 to 1.18) |
| 30-34 | 9.6 (9.4 to 9.8) | 1 | 2.9 (2.8 to 3.0) | 1 |
| 35-39 | 9.3 (9.1 to 9.6) | 0.99 (0.95 to 1.03) | 2.9 (2.7 to 3.0) | 0.99 (0.92 to 1.07) |
| 40-49 | 9.6 (9.0 to 10.1) | 1.01 (0.94 to 1.07) | 3.1 (2.7 to 3.4) | 1.05 (0.93 to 1.19) |
| Calendar period | | | | |
| 2000-2004 | 11.4 (11.1 to 11.7) | 1 | 2.4 (2.2 to 2.5) | 1 |
| 2005-2009 | 11.3 (11.1 to 11.6) | 0.97 (0.93 to 1.01) | 3.5 (3.3 to 3.6) | 1.43 (1.30 to 1.57) |
| 2010-2013 | 11.5 (11.2 to 11.7) | 0.96 (0.92 to 1.01) | 3.8 (3.6 to 3.9) | 1.54 (1.38 to 1.71) |
| Townsend deprivation index quantile | | | | |
| 1 | 8.9 (8.7 to 9.2) | 1 | 2.7 (2.5 to 2.8) | 1 |
| 2 | 10.0 (9.7 to 10.3) | 1.09 (1.04 to 1.14) | 3.0 (2.9 to 3.2) | 1.10 (1.00 to 1.20) |
| 3 | 11.3 (11.0 to 11.6) | 1.19 (1.14 to 1.25) | 3.3 (3.1 to 3.4) | 1.14 (1.04 to 1.25) |
| 4 | 13.1 (12.7 to 13.4) | 1.33 (1.25 to 1.40) | 3.8 (3.6 to 4.0) | 1.29 (1.17 to 1.42) |
| 5 | 15.2 (14.8 to 15.6) | 1.47 (1.38 to 1.57) | 4.2 (3.9 to 4.4) | 1.36 (1.22 to 1.52) |

For peer review only



For peer review only - http://bmjopen.bmj.com/site/about/guidelines.xhtml

## References


1. Beck CT. Predictors of postpartum depression: an update. Nurs Res. 2001 Oct;50(5):275–85.

2. Gavin NI, Gaynes BN, Lohr KN, Meltzer-Brody S, Gartlehner G, Swinson T. Perinatal depression: A systematic review of prevalence and incidence. Obstet Gynecol. 2005;106(5 I):1071–83.

3. O'hara MW, Swain AM. Rates and risk of postpartum depression—a meta-analysis. Int Rev Psychiatry. 1996 Jan;8(1):37–54.

4. Bauer A, Parsonage M, Knapp M, Iemmi V, Adelaja B. Costs of perinatal mental health problems [Internet]. 2014 [cited 2015 Nov 6]. Available from: http://www.centreformentalhealth.org.uk/

5. Jones I, Shakespeare J. Postnatal depression. The BMJ. 2014 Aug 14;349:g4500.

6. Kingston D, Tough S, Whitfield H. Prenatal and postpartum maternal psychological distress and infant development: a systematic review. Child Psychiatry Hum Dev. 2012 Oct;43(5):683–714.

7. Robertson E, Grace S, Wallington T, Stewart DE. Antenatal risk factors for postpartum depression: a synthesis of recent literature. Gen Hosp Psychiatry. 2004 Aug;26(4):289–95.

8. Howard LM, Megnin-Viggars O, Symington I, Pilling S, On behalf of the Guideline Development Group. Antenatal and postnatal mental health: summary of updated NICE guidance. BMJ. 2014 Dec 18;349(dec18 5):g7394–g7394.

9. Stewart DE, Vigod S. Postpartum Depression. N Engl J Med. 2016 01;375(22):2177–86.

10. Chisholm J. The Read clinical classification. Br Med J. 1990 Apr 28;300(6732):1092.

11. Davé S, Petersen I. Creating medical and drug code lists to identify cases in primary care databases. Pharmacoepidemiol Drug Saf. 2009;18(8):704–7.

12. Townsend, Phillimore, Beattie A. Inequalities in health in the northern region. Newcastle upon Tyne: Northern Regional Health Authority and University of Bristol. University of Bristol; 1986.

13. Lis Y, Mann RD. The VAMP research multi-purpose database in the UK. J Clin Epidemiol. 1995 Mar;48(3):431–43.

14. Blak BT, Thompson M, Dattani H, Bourke A. Generalisability of The Health Improvement Network (THIN) database: demographics, chronic disease prevalence and mortality rates. Inform Prim Care. 2011;19(4):251–5.

15. Williams T, van Staa T, Puri S, Eaton S. Recent advances in the utility and use of the General Practice Research Database as an example of a UK Primary Care Data resource. Ther Adv Drug Saf. 2012 Apr;3(2):89–99.

16. Rasmussen CE, Williams CKI. Gaussian processes for machine learning. Cambridge, Mass: MIT Press; 2006. 248 p. (Adaptive computation and machine learning).




For peer review only - http://bmjopen.bmj.com/site/about/guidelines.xhtml

17. Rait G, Walters K, Griffin M, Buszewicz M, Petersen I, Nazareth I. Recent trends in the incidence of recorded depression in primary care. Br J Psychiatry. 2009 Dec 1;195(6):520–4.

18. Milgrom J, Gemmill AW, Bilszta JL, Hayes B, Barnett B, Brooks J, et al. Antenatal risk factors for postnatal depression: a large prospective study. J Affect Disord. 2008 May;108(1-2):147–57.

19. Petersen I, Gilbert RE, Evans SJW, Man S-L, Nazareth I. Pregnancy as a major determinant for discontinuation of antidepressants: an analysis of data from The Health Improvement Network. J Clin Psychiatry. 2011 Jul;72(7):979–85.

20. Charlton R, Jordan S, Pierini A, Garne E, Neville A, Hansen A, et al. Selective serotonin reuptake inhibitor prescribing before, during and after pregnancy: a population-based study in six European regions. BJOG Int J Obstet Gynaecol. 2015 Jun;122(7):1010–20.

21. Cohen LS. Relapse of Major Depression During Pregnancy in Women Who Maintain or Discontinue Antidepressant Treatment. JAMA. 2006 Feb 1;295(5):499.

22. Troutman BR, Cutrona CE. Nonpsychotic postpartum depression among adolescent mothers. J Abnorm Psychol. 1990 Feb;99(1):69–78.

23. Hobfoll SE, Ritter C, Lavin J, Hulsizer MR, Cameron RP. Depression prevalence and incidence among inner-city pregnant and postpartum women. J Consult Clin Psychol. 1995 Jun;63(3):445–53.

24. McCrea RL, Sammon CJ, Nazareth I, Petersen I. Initiation and duration of selective serotonin reuptake inhibitor prescribing over time: UK cohort study. Br J Psychiatry. 2016 Nov 1;209(5):421–6.

25. Dennis C-L, Chung-Lee L. Postpartum Depression Help-Seeking Barriers and Maternal Treatment Preferences: A Qualitative Systematic Review. Birth. 2006 Dec;33(4):323–31.

26. National Institute for Health and Care Excellence (NICE). Antenatal and postnatal mental health: clinical management and service guidance [Internet]. 2014 Dec. Available from: http://guidance.nice.org.uk/cg185

27. Di Florio A, Putnam K, Altemus M, Apter G, Bergink V, Bilszta J, et al. The impact of education, country, race and ethnicity on the self-report of postpartum depression using the Edinburgh Postnatal Depression Scale. Psychol Med. 2017 Apr;47(5):787–99.

28. O'Connor E, Rossom RC, Henninger M, Groom HC, Burda BU. Primary Care Screening for and Treatment of Depression in Pregnant and Postpartum Women: Evidence Report and Systematic Review for the US Preventive Services Task Force. JAMA. 2016 Jan 26;315(4):388–406.



For peer review only - http://bmjopen.bmj.com/site/about/guidelines.xhtml

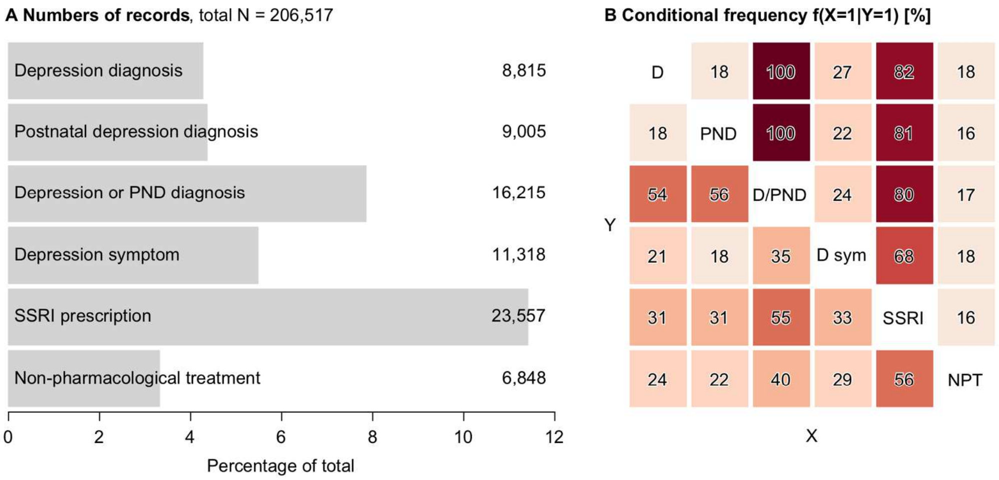


160x76mm (300 x 300 DPI)

For peer review only

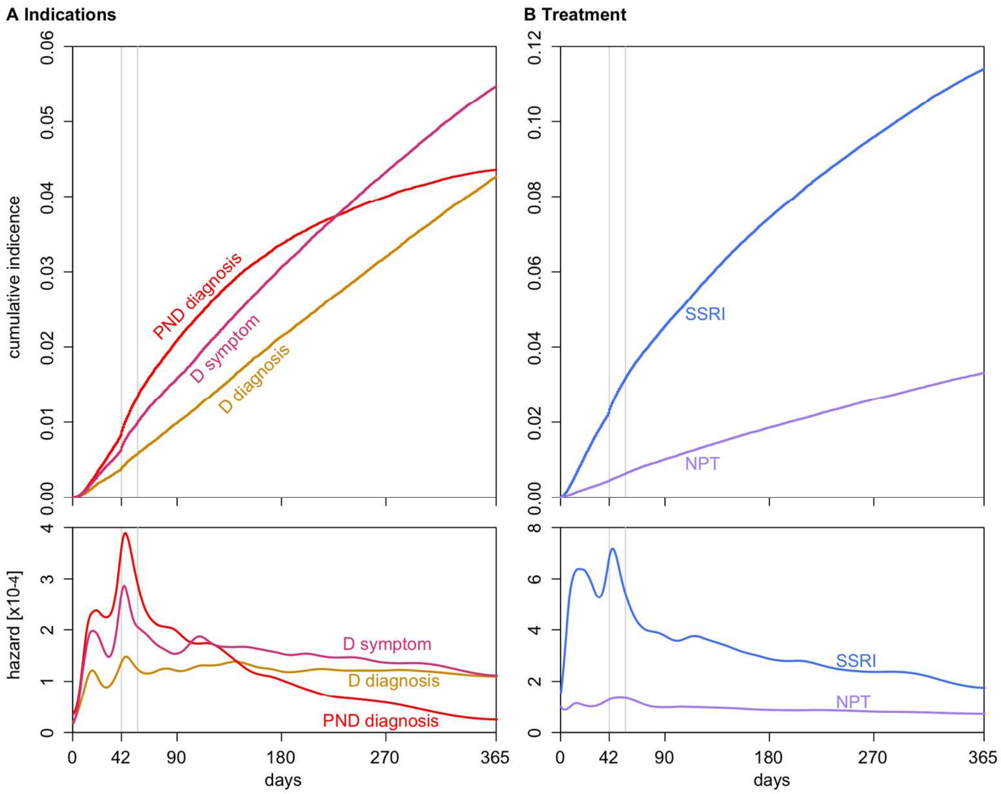


160x127mm (300 x 300 DPI)

For peer review only

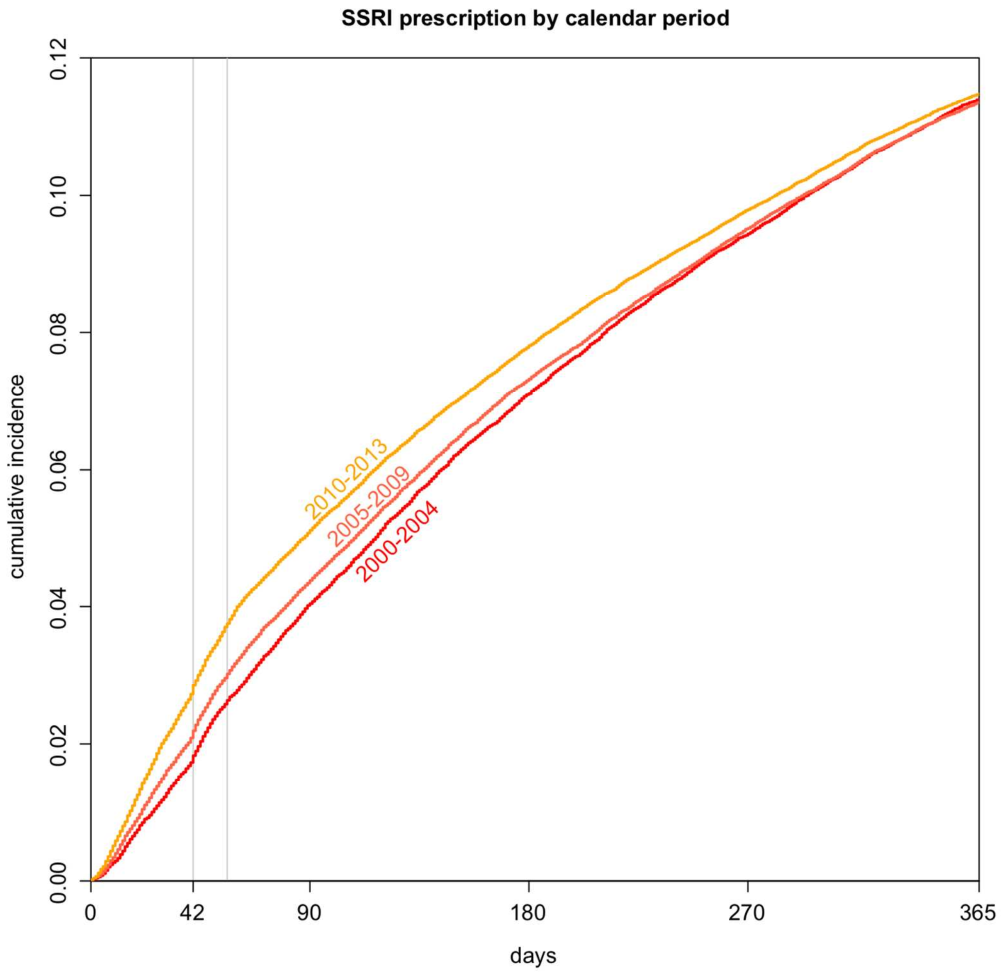


127x123mm (300 x 300 DPI)

STable 1. Rates and relative risk estimates of depression diagnosis, postnatal depression diagnosis and depression symptoms in the first year after delivery for 142,234 women who gave birth between 2000 and 2013 and had no prior record suggestive of depression. Adjusted by age group, calendar period, and Townsend deprivation index. RR = relative risk.

| | Depression diagnosis | | Postnatal depression diagnosis | | Depression symptom | |
|---|---|---|---|---|---|---|
| | Rate per 100 | Adjusted RR | Rate per 100 | Adjusted RR | Rate per 100 | Adjusted RR |
| Age group | | | | | | |
| 15-20 | 4.9 (4.4 to 5.3) | 2.74 (2.40 to 3.13) | 6.6 (6.0 to 7.1) | 2.88 (2.56 to 3.23) | 8.4 (7.8 to 9.0) | 3.35 (3.01 to 3.72) |
| 20-25 | 3.8 (3.5 to 4.0) | 2.21 (1.97 to 2.47) | 4.4 (4.1 to 4.7) | 1.98 (1.80 to 2.17) | 5.2 (4.9 to 5.6) | 2.13 (1.95 to 2.33) |
| 25-30 | 2.2 (2.0 to 2.4) | 1.33 (1.21 to 1.47) | 3.0 (2.8 to 3.2) | 1.33 (1.22 to 1.45) | 3.0 (2.8 to 3.1) | 1.26 (1.16 to 1.37) |
| 30-35 | 1.6 (1.5 to 1.7) | 1 | 2.3 (2.1 to 2.4) | 1 | 2.3 (2.1 to 2.4) | 1 |
| 35-40 | 1.4 (1.3 to 1.6) | 0.90 (0.81 to 1.01) | 2.0 (1.8 to 2.2) | 0.89 (0.80 to 0.98) | 2.0 (1.9 to 2.2) | 0.90 (0.82 to 0.99) |
| 40-50 | 1.2 (0.9 to 1.5) | 0.75 (0.59 to 0.95) | 1.8 (1.5 to 2.1) | 0.81 (0.67 to 0.99) | 1.8 (1.5 to 2.1) | 0.79 (0.64 to 0.96) |
| Calendar period | | | | | | |
| 2000-2004 | 3.0 (2.8 to 3.2) | 1 | 3.9 (3.7 to 4.1) | 1 | 2.6 (2.4 to 2.8) | 1 |
| 2005-2009 | 2.1 (2.0 to 2.2) | 0.67 (0.60 to 0.75) | 2.9 (2.7 to 3.0) | 0.70 (0.64 to 0.77) | 3.4 (3.3 to 3.6) | 1.27 (1.15 to 1.40) |
| 2010-2013 | 1.7 (1.6 to 1.8) | 0.53 (0.47 to 0.60) | 2.1 (2.0 to 2.3) | 0.52 (0.47 to 0.57) | 3.1 (2.9 to 3.2) | 1.13 (1.01 to 1.26) |
| Townsend deprivation index quantile | | | | | | |
| 1 | 1.6 (1.5 to 1.8) | 1 | 2.4 (2.2 to 2.5) | 1 | 2.3 (2.1 to 2.4) | 1 |
| 2 | 1.9 (1.7 to 2.0) | 1.11 (0.98 to 1.26) | 2.6 (2.4 to 2.8) | 1.06 (0.97 to 1.17) | 2.6 (2.4 to 2.7) | 1.05 (0.95 to 1.15) |
| 3 | 2.1 (2.0 to 2.3) | 1.19 (1.05 to 1.34) | 3.0 (2.8 to 3.2) | 1.17 (1.06 to 1.29) | 3.1 (2.9 to 3.3) | 1.18 (1.06 to 1.31) |
| 4 | 2.6 (2.4 to 2.8) | 1.34 (1.18 to 1.51) | 3.3 (3.1 to 3.5) | 1.16 (1.04 to 1.29) | 3.8 (3.6 to 4.0) | 1.28 (1.15 to 1.42) |
| 5 | 3.1 (2.8 to 3.3) | 1.42 (1.24 to 1.64) | 3.5 (3.2 to 3.7) | 1.10 (0.96 to 1.27) | 4.4 (4.1 to 4.7) | 1.32 (1.18 to 1.48) |

For peer review only

STable 2. Rates and relative risk estimates of SSRI prescription and non-pharmacological treatment in the first year after delivery for 142,234 women who gave birth between 2000 and 2013 and had no prior record suggestive of depression. Adjusted by age group, calendar period, and Townsend deprivation index. RR = relative risk.

| | SSRI prescription | | Non-pharmacological treatment | |
|---|---|---|---|---|
| | Rate per 100 | Adjusted RR | Rate per 100 | Adjusted RR |
| Age group | | | | |
| 15-20 | 14.2 (13.4 to 15.0) | 3.10 (2.85 to 3.37) | 3.4 (3.0 to 3.8) | 2.48 (2.12 to 2.90) |
| 20-25 | 9.9 (9.4 to 10.3) | 2.20 (2.06 to 2.36) | 2.2 (2.0 to 2.4) | 1.60 (1.40 to 1.84) |
| 25-30 | 5.8 (5.5 to 6.0) | 1.32 (1.24 to 1.41) | 1.6 (1.5 to 1.8) | 1.21 (1.08 to 1.36) |
| 30-35 | 4.3 (4.1 to 4.5) | 1 | 1.3 (1.2 to 1.4) | 1 |
| 35-40 | 3.7 (3.5 to 3.9) | 0.86 (0.80 to 0.93) | 1.3 (1.1 to 1.4) | 0.94 (0.83 to 1.07) |
| 40-50 | 3.1 (2.7 to 3.5) | 0.73 (0.63 to 0.84) | 1.2 (0.9 to 1.4) | 0.87 (0.69 to 1.11) |
| Calendar period | | | | |
| 2000-2004 | 6.5 (6.3 to 6.8) | 1 | 1.2 (1.1 to 1.3) | 1 |
| 2005-2009 | 5.8 (5.7 to 6.0) | 0.86 (0.81 to 0.91) | 1.8 (1.7 to 1.9) | 1.42 (1.25 to 1.62) |
| 2010-2013 | 5.1 (4.9 to 5.3) | 0.75 (0.70 to 0.80) | 1.7 (1.6 to 1.9) | 1.40 (1.22 to 1.61) |
| Townsend deprivation index quantile | | | | |
| 1 | 4.5 (4.3 to 4.7) | 1 | 1.4 (1.2 to 1.5) | 1 |
| 2 | 4.9 (4.7 to 5.2) | 1.05 (0.98 to 1.13) | 1.5 (1.4 to 1.6) | 1.06 (0.92 to 1.23) |
| 3 | 5.9 (5.6 to 6.1) | 1.15 (1.07 to 1.24) | 1.6 (1.5 to 1.8) | 1.08 (0.93 to 1.24) |
| 4 | 6.7 (6.4 to 7.0) | 1.18 (1.09 to 1.28) | 1.8 (1.7 to 2.0) | 1.12 (0.97 to 1.30) |
| 5 | 8.0 (7.6 to 8.4) | 1.26 (1.13 to 1.40) | 2.0 (1.8 to 2.2) | 1.12 (0.94 to 1.32) |

For peer review only

STable 3. Rates and relative risk estimates of depression diagnosis, postnatal depression diagnosis and depression symptoms in the first year after delivery for 64,283 women who gave birth between 2000 and 2013 and had a prior record suggestive of depression. Adjusted by age group, calendar period, and Townsend deprivation index. RR = relative risk.

| | Depression diagnosis | | Postnatal depression diagnosis | | Depression symptom | |
|---|---|---|---|---|---|---|
| | Rate per 100 | Adjusted RR | Rate per 100 | Adjusted RR | Rate per 100 | Adjusted RR |
| Age group | | | | | | |
| 15-20 | 12.6 (11.3 to 14.0) | 1.48 (1.31 to 1.67) | 11.1 (9.8 to 12.4) | 1.50 (1.32 to 1.71) | 18.3 (16.7 to 19.9) | 1.76 (1.58 to 1.95) |
| 20-25 | 10.6 (10.0 to 11.2) | 1.24 (1.15 to 1.35) | 8.2 (7.7 to 8.7) | 1.11 (1.02 to 1.21) | 13.3 (12.7 to 14.0) | 1.29 (1.22 to 1.38) |
| 25-30 | 9.2 (8.7 to 9.6) | 1.10 (1.03 to 1.17) | 7.9 (7.5 to 8.3) | 1.07 (0.99 to 1.15) | 10.6 (10.1 to 11.1) | 1.06 (0.99 to 1.12) |
| 30-35 | 8.3 (7.9 to 8.7) | 1 | 7.6 (7.2 to 8.0) | 1 | 9.8 (9.3 to 10.2) | 1 |
| 35-40 | 8.0 (7.6 to 8.5) | 0.98 (0.91 to 1.06) | 6.8 (6.3 to 7.2) | 0.90 (0.82 to 0.97) | 9.2 (8.7 to 9.7) | 0.95 (0.88 to 1.01) |
| 40-50 | 6.7 (5.8 to 7.5) | 0.84 (0.73 to 0.97) | 5.7 (4.9 to 6.5) | 0.78 (0.67 to 0.89) | 9.9 (8.9 to 11.0) | 1.02 (0.91 to 1.14) |
| Calendar period | | | | | | |
| 2000-2004 | 12.7 (12.1 to 13.2) | 1 | 10.8 (10.2 to 11.3) | 1 | 9.1 (8.7 to 9.6) | 1 |
| 2005-2009 | 8.8 (8.4 to 9.1) | 0.68 (0.63 to 0.74) | 7.7 (7.3 to 8.0) | 0.71 (0.66 to 0.77) | 11.7 (11.3 to 12.1) | 1.26 (1.16 to 1.38) |
| 2010-2013 | 7.0 (6.7 to 7.3) | 0.54 (0.50 to 0.59) | 5.9 (5.6 to 6.2) | 0.54 (0.50 to 0.59) | 10.6 (10.3 to 11.0) | 1.14 (1.03 to 1.25) |
| Townsend deprivation index quantile | | | | | | |
| 1 | 7.6 (7.2 to 8.1) | 1 | 7.6 (7.1 to 8.0) | 1 | 9.2 (8.7 to 9.7) | 1 |
| 2 | 8.1 (7.6 to 8.6) | 1.06 (0.98 to 1.16) | 7.8 (7.3 to 8.2) | 1.04 (0.95 to 1.13) | 9.5 (9.0 to 10.1) | 1.01 (0.93 to 1.11) |
| 3 | 8.5 (8.0 to 8.9) | 1.10 (1.01 to 1.20) | 7.1 (6.6 to 7.5) | 0.93 (0.85 to 1.03) | 10.0 (9.5 to 10.5) | 1.04 (0.95 to 1.13) |
| 4 | 9.7 (9.2 to 10.2) | 1.25 (1.15 to 1.36) | 7.7 (7.2 to 8.1) | 1.00 (0.92 to 1.09) | 11.9 (11.4 to 12.4) | 1.21 (1.11 to 1.32) |
| 5 | 10.6 (10.0 to 11.1) | 1.33 (1.20 to 1.47) | 8.2 (7.7 to 8.7) | 1.05 (0.94 to 1.16) | 13.0 (12.4 to 13.6) | 1.29 (1.16 to 1.43) |

For peer review only

STable 4. Rates and relative risk estimates of SSRI prescription and non-pharmacological treatment in the first year after delivery for 64,283 women who gave birth between 2000 and 2013 and had a prior record suggestive of depression. Adjusted by age group, calendar period, and Townsend deprivation index. RR = relative risk.

| | SSRI prescription | | Non-pharmacological treatment | |
|---|---|---|---|---|
| | Rate per 100 | Adjusted RR | Rate per 100 | Adjusted RR |
| Age group | | | | |
| 15-20 | 34.3 (32.4 to 36.3) | 1.47 (1.38 to 1.57) | 10.1 (8.8 to 11.3) | 1.42 (1.25 to 1.62) |
| 20-25 | 27.3 (26.4 to 28.1) | 1.18 (1.13 to 1.23) | 8.3 (7.8 to 8.9) | 1.18 (1.09 to 1.29) |
| 25-30 | 23.9 (23.2 to 24.5) | 1.04 (1.01 to 1.08) | 6.8 (6.4 to 7.2) | 0.99 (0.91 to 1.06) |
| 30-35 | 22.6 (22.0 to 23.2) | 1 | 6.8 (6.4 to 7.1) | 1 |
| 35-40 | 22.0 (21.2 to 22.7) | 0.98 (0.94 to 1.02) | 6.5 (6.0 to 6.9) | 0.95 (0.87 to 1.04) |
| 40-50 | 21.2 (19.8 to 22.6) | 0.94 (0.88 to 1.01) | 6.5 (5.7 to 7.4) | 0.95 (0.83 to 1.09) |
| Calendar period | | | | |
| 2000-2004 | 24.0 (23.3 to 24.8) | 1 | 5.4 (5.0 to 5.8) | 1 |
| 2005-2009 | 23.6 (23.1 to 24.1) | 0.97 (0.93 to 1.02) | 7.3 (7.0 to 7.6) | 1.34 (1.21 to 1.49) |
| 2010-2013 | 24.1 (23.6 to 24.6) | 0.99 (0.95 to 1.03) | 7.8 (7.5 to 8.1) | 1.43 (1.27 to 1.61) |
| Townsend deprivation index quantile | | | | |
| 1 | 21.7 (21.0 to 22.4) | 1 | 6.5 (6.0 to 6.9) | 1 |
| 2 | 22.7 (22.0 to 23.5) | 1.04 (0.99 to 1.09) | 6.8 (6.4 to 7.3) | 1.04 (0.93 to 1.16) |
| 3 | 23.2 (22.5 to 23.9) | 1.04 (0.99 to 1.10) | 6.9 (6.5 to 7.3) | 1.02 (0.92 to 1.14) |
| 4 | 25.0 (24.3 to 25.7) | 1.11 (1.06 to 1.16) | 7.5 (7.1 to 8.0) | 1.10 (0.99 to 1.23) |
| 5 | 26.6 (25.8 to 27.4) | 1.16 (1.10 to 1.22) | 7.6 (7.2 to 8.1) | 1.10 (0.97 to 1.25) |

For peer review only

**STROBE 2007 (v4) Statement—Checklist of items that should be included in reports of *cohort studies***

| Section/Topic | Item # | Recommendation | Reported on page # |
|---|---|---|---|
| **Title and abstract** | 1 | (*a*) Indicate the study's design with a commonly used term in the title or the abstract | 1 |
| | | (*b*) Provide in the abstract an informative and balanced summary of what was done and what was found | 2 |
| **Introduction** | | | |
| Background/rationale | 2 | Explain the scientific background and rationale for the investigation being reported | 4 |
| Objectives | 3 | State specific objectives, including any prespecified hypotheses | 4 |
| **Methods** | | | |
| Study design | 4 | Present key elements of study design early in the paper | 5 |
| Setting | 5 | Describe the setting, locations, and relevant dates, including periods of recruitment, exposure, follow-up, and data collection | 5 |
| Participants | 6 | (*a*) Give the eligibility criteria, and the sources and methods of selection of participants. Describe methods of follow-up | 5 |
| | | (*b*) For matched studies, give matching criteria and number of exposed and unexposed | N/A |
| Variables | 7 | Clearly define all outcomes, exposures, predictors, potential confounders, and effect modifiers. Give diagnostic criteria, if applicable | 5 |
| Data sources/ measurement | 8* | For each variable of interest, give sources of data and details of methods of assessment (measurement). Describe comparability of assessment methods if there is more than one group | 5 |
| Bias | 9 | Describe any efforts to address potential sources of bias | 6 |
| Study size | 10 | Explain how the study size was arrived at | 5 |
| Quantitative variables | 11 | Explain how quantitative variables were handled in the analyses. If applicable, describe which groupings were chosen and why | 6 |
| Statistical methods | 12 | (*a*) Describe all statistical methods, including those used to control for confounding | 6 |
| | | (*b*) Describe any methods used to examine subgroups and interactions | 6 |
| | | (*c*) Explain how missing data were addressed | 6 |
| | | (*d*) If applicable, explain how loss to follow-up was addressed | 6 |
| | | (*e*) Describe any sensitivity analyses | 6 |
| **Results** | | | |

For peer review only

| | | | |
|---|---|---|---|
| Participants | 13* | (a) Report numbers of individuals at each stage of study—eg numbers potentially eligible, examined for eligibility, confirmed eligible, included in the study, completing follow-up, and analysed | 7 |
| | | (b) Give reasons for non-participation at each stage | N/A |
| | | (c) Consider use of a flow diagram | N/A |
| Descriptive data | 14* | (a) Give characteristics of study participants (eg demographic, clinical, social) and information on exposures and potential confounders | 7 |
| | | (b) Indicate number of participants with missing data for each variable of interest | N/A |
| | | (c) Summarise follow-up time (eg, average and total amount) | 7 |
| Outcome data | 15* | Report numbers of outcome events or summary measures over time | 7 |
| Main results | 16 | (*a*) Give unadjusted estimates and, if applicable, confounder-adjusted estimates and their precision (eg, 95% confidence interval). Make clear which confounders were adjusted for and why they were included | 8 |
| | | (*b*) Report category boundaries when continuous variables were categorized | 8 |
| | | (*c*) If relevant, consider translating estimates of relative risk into absolute risk for a meaningful time period | 8 |
| Other analyses | 17 | Report other analyses done—eg analyses of subgroups and interactions, and sensitivity analyses | 8-9 |
| **Discussion** | | | |
| Key results | 18 | Summarise key results with reference to study objectives | 9 |
| **Limitations** | | | |
| Interpretation | 20 | Give a cautious overall interpretation of results considering objectives, limitations, multiplicity of analyses, results from similar studies, and other relevant evidence | 10 |
| Generalisability | 21 | Discuss the generalisability (external validity) of the study results | 10 |
| **Other information** | | | |
| Funding | 22 | Give the source of funding and the role of the funders for the present study and, if applicable, for the original study on which the present article is based | 12 |

*Give information separately for cases and controls in case-control studies and, if applicable, for exposed and unexposed groups in cohort and cross-sectional studies.

**Note:** An Explanation and Elaboration article discusses each checklist item and gives methodological background and published examples of transparent reporting. The STROBE checklist is best used in conjunction with this article (freely available on the Web sites of PLoS Medicine at http://www.plosmedicine.org/, Annals of Internal Medicine at http://www.annals.org/, and Epidemiology at http://www.epidem.com/). Information on the STROBE Initiative is available at www.strobe-statement.org.

For peer review only

BMJ Open

# Depression, depressive symptoms and treatments in women who have recently given birth: UK cohort study

| | |
|---|---|
| Journal: | *BMJ Open* |
| Manuscript ID | bmjopen-2018-022152.R1 |
| Article Type: | Research |
| Date Submitted by the Author: | 06-Jun-2018 |
| Complete List of Authors: | Petersen, Irene; University College London Research Department of Primary Care and Population Health<br>Peltola, Tomi; Aalto University, Helsinki Institute for Information Technology HIIT<br>Kaski, Samuel; Aalto University, Helsinki Institute for Information Technology HIIT<br>Walters, Kate; University College London Research Department of Primary Care and Population Health<br>Hardoon, Sarah; University College London Research Department of Primary Care and Population Health |
| <b>Primary Subject Heading</b>: | Mental health |
| Secondary Subject Heading: | General practice / Family practice, Patient-centred medicine |
| Keywords: | postnatal depression, SSRI treatment, non-pharmacological treatment, PRIMARY CARE, Depression & mood disorders < PSYCHIATRY |
| | |

only

SCHOLARONE™
Manuscripts

# Depression, depressive symptoms and treatments in women who have recently given birth: UK cohort study

Irene Petersen, PhD (1, 2), Tomi Peltola, PhD (1,3), Samuel Kaski, PhD (3), Kate Walters, PhD (1) and Sarah Hardoon, PhD (1)

Affiliations:

1) Department of Primary Care and Population Health, University College London, Rowland Hill St, London NW3 2PF, UK

2) Department of Clinical Epidemiology, Aarhus University, Olof Palmes Alle 43 – 45, 8200 Aarhus N, Denmark

3) Helsinki Institute for Information Technology HIIT, Department of Computer Science, Aalto University, Konemiehentie 2, 02150 Espoo, Finland

Corresponding author: Irene Petersen, e-mail: i.petersen@ucl.ac.uk, Department of Primary Care and Population Health, UCL, Rowland Hill St, London NW3 2PF, UK.

Word count: 3387

For peer review only

## Abstract

Objectives: To investigate how depression is recognised in the year after child birth and treatment given in clinical practice.

Design: Cohort study

Setting: Primary Care

Participants: Women who have given live birth between 2000 and 2013.

Outcomes: Prevalence of postnatal depression, depression, depressive symptoms, antidepressant and non-pharmacological treatment within a year after birth.

Results: Of 206,517 women 23,623 (11%) had a record of depressive diagnosis or symptoms in the year after delivery and more than 1 in 8 women received antidepressant treatment. Recording and treatment peaked 6 to 8 weeks after delivery. Initiation of SSRI treatment has become earlier in the more recent years. Thus, the initiation rate of SSRI treatment per 100 pregnancies (95% CI) at 8 weeks were 2.6 (2.5 to 2.8) in 2000-2004, increasing to 3.0 (2.9 to 3.1) in 2005-2009, and 3.8 (3.6 to 3.9) in 2010-2013. The overall rate of initiation of SSRI within the year after delivery, however, has not changed noticeably. A third of the women had at least one record suggestive of depression at any time prior to delivery and of these 1 in 4 received SSRI treatment in the year after delivery.

Younger women were most likely to have records of depression and depressive symptoms. Relative Risk for postnatal depression: Age 15 – 19: 1.92 (1.76 to 2.10), Age 20 – 24: 1.49 (1.39 to 1.59) versus Age 30 – 34)). The risk of depression, postnatal depression and depressive symptoms increased with increasing social deprivation.

Conclusions: More than 1 in 10 women had electronic health records indicating depression or depressive symptoms within a year after delivery and more than 1 in 8 women received antidepressant treatment in this period. Women aged below 30 and from the most deprived areas were at highest risk depression and most likely to receive antidepressant treatment.


## Summary

Strength and limitations of this study



For peer review only - http://bmjopen.bmj.com/site/about/guidelines.xhtml

- A major strength of this study is that we have access to a very large sample of primary care electronic health records of women who gave live birth.
- These records reflect clinical practice in UK primary care and were made prospectively.
- We considered a broad definition of depression on clinical evaluation in the year after delivery as there are no specific guidelines to how it should be recorded.
- This study may overestimate the number of women with postnatal depression compared to estimates based on a diagnostic interview and specific diagnostic instruments.
- Non-pharmacological treatment may not be well recorded in primary care electronic health records.

For peer review only



For peer review only - http://bmjopen.bmj.com/site/about/guidelines.xhtml

## Introduction

Many women experience depression in the year after they have given birth. Postnatal depression affects an estimated 10 – 19% of women, although the estimates vary substantially between countries and settings. (1–4) Depression may have severe consequences for the mother and, in turn, have physical, cognitive, and emotional effects on their children's development, continuing into later life. (5–8) A report published by the London School of Economics estimated that perinatal depression, anxiety and psychosis carry a total long-term cost to society of about £6.6 billion for each one-year cohort of births in the UK. (5) This is equivalent to a cost of just under £10,000 for every single birth in the country. Nearly three-quarters (72%) of this cost relates to adverse impacts on the child rather than the mother. (5)

Guidelines in both US and UK on antenatal and postnatal mental health recommend that health care professionals should consider asking simple screening questions about current and past histories of depression, anxiety, alcohol and illicit drug use as part of a general discussion about mental health and wellbeing in pregnancy and the perinatal period. (9,10) However, very limited information is available on when depression is recognised and how it is treated in clinical practice in the year after women have given birth. For most women who experience depression in this period primary care physicians would be a first point of contact. In this study, we sought to obtain an overview of actual clinical practice in UK primary care by examining electronic health records on more than 200,000 women who have given life birth between 2000 and 2013. We followed the women for a year after delivery and our aim was to examine how and when depression and depressive symptoms were recorded and treatment provided in general practice and the interrelation between antidepressant and non-pharmacological treatment.

For peer review only

# Methods

## Data source

We used data from The Health Improvement Network (THIN). This is a large primary care database that provides anonymised longitudinal general practice (family practice) data on patients' clinical and prescribing records and includes data from around 6% of the United Kingdom population. Diagnoses and symptoms are recorded by practice staff using Read codes, which is a hierarchical coding system including more than 100,000 codes. (11,12) The Read code system can be mapped to ICD-10, but in addition the Read codes include a number of symptoms and administrative codes. (12) Prescriptions are issued electronically and directly recorded on the general practice computer systems. In addition, the database holds individual patient level information about year of birth, date of registration, date of death and transfer out of the practice and information about social deprivation (quintiles of Townsend deprivation scores). The Townsend scores is based on census data (2011) for car ownership, owner-occupation, overcrowding and unemployment in a patient's postcode. (13)

Over 98% of the UK population are registered with a general practitioner (GP) (14) and the UK primary care databases are broadly representative of the United Kingdom population. (15,16) While perinatal care is often shared between general practice staff and midwives, the GP remains responsible for women's general medical care including continued prescribing of medicines such as antidepressants. Some women may also receive care from local National Health Service (NHS) mental health trusts, but trusts have limited prescribing budgets and for most women prescribing of psychotropic medication remains with the GP. Furthermore, after a few weeks after delivery the care by the midwife ends and general practitioners are the first point of contact. Typically, women will consult their general practitioner for a postnatal maternal check-up at 6 to 8 weeks after delivery.

## Study population

We utilised data from women who have given live birth between 1st January 2000 and 31st December 2013 and who were permanently registered with the same general practice for at least one year after delivery. As some women had more than one pregnancy and the risk of postnatal depression may be strongly correlated within women we randomly selected one pregnancy per woman for our analyses.

## Variables

We identified women with one or more records entered as a Read code in their primary care electronic health records which suggested they had depression, postnatal depression or symptoms of depression



For peer review only - http://bmjopen.bmj.com/site/about/guidelines.xhtml

as well women on antidepressant and non-pharmacological treatment (referral to counselling and psychotherapy) in the year after they have given birth. Antidepressant treatment was classified as selective serotonin reuptake inhibitors (SSRI), TCA and other antidepressants. For TCA we only considered treatment that was prescribed above treatment threshold for depression, as lower doses may be prescribed for other reasons such as chronic pain. In addition, we included information on calendar year of delivery, age at delivery and social deprivation.

## Data analysis

First, we estimated the prevalence of any records directly suggestive of depression (postnatal depression, depression diagnoses, depressive symptoms) as well as separate estimates for postnatal depression, depression diagnoses, depressive symptoms, antidepressant or non-pharmacological treatments within a year after giving birth. We then estimated how the records were interrelated. Interrelations were reported as conditional frequencies, that is, the frequency of having a record of X given that one has a record of Y. These frequencies are reported in Figure 1B. For example, the figure illustrates that 82% of those who had a diagnosis of depression also had a prescription of a SSRI. On the other hand, 31% of those who had a prescription of SSRI had a diagnosis of depression.

We estimated the timing of the recording within the follow-up year and report cumulative incidence curves (as one minus the Kaplan-Meier estimate). We also estimated smoothed daily hazards using a Gaussian process model (17) to visualize the daily changes in the timing of recording.

For each of the three depression outcomes (depression diagnosis, postnatal depression diagnosis and depression symptoms) and for SSRI and non-pharmacological treatments, we used Poisson regression to model relative risks of having a record associated with age, calendar time and social deprivation (Townsend scores). Age was split into six age groups (15-19, 20-24, 25-29, 30-34, 35-39, 40-49) and calendar time into three periods (2000-2004, 2005-2009, 2010-2013). 95% CI were computed using modified Poisson regression accounting for the clustering of women in general practices. We conducted supportive analyses stratified 1) on whether women had any record suggestive of depression or treatment prior to delivery. 2) on whether the women had early or late records of depression or treatment. In the latter analyses we categorised women into two groups; women who had a record of depression or treatments before 42 days after delivery were considered as having an *early* record and women who had a record of depression or treatments after 42 days of delivery were considered as having a *late* record. We investigated whether this was associated with age, social deprivation, calendar time and any record suggestive of depression or treatment prior to delivery using logistic regression.



For peer review only - http://bmjopen.bmj.com/site/about/guidelines.xhtml

## Ethics

The scheme for THIN to obtain and provide anonymous patient data to researchers was approved by the National Health Service South-East Multicenter Research Ethics Committee (MREC) in 2002 and scientific approval for this study was obtained from IMS Scientific Review Committee.

## Patient and Public Involvement

Charlotte Walker, who is a mental health service user, has been involved with the original design of the study proposal and provided feedback on this manuscript and thus helped to shape the discussion of the paper from a service user's perspective.

For peer review only

## Results

In total, 206,517 women were included in the study and there were 23,623 (11%) with at least one record directly suggestive of depression (depression, postnatal depression or symptoms of depression) in the year after delivery.  Of these women, there were 4% with a record of depression, 4% with a record of postnatal depression and 5% with symptoms of depression (Figure 1A). Of those women with a depression diagnosis, 2,349/8,815 (27%) also had depressive symptoms (Figure 1B), and of those with postnatal depression diagnosis, 2,005/9,005(22%) also had depressive symptoms (Figure 1B). In contrast, there were 7,408/11,318 (65%) women with a record of depressive symptoms *without* either a depression diagnosis or postnatal depression diagnosis.

The number of women with a record suggestive of depression continued to rise throughout the first year after delivery (Figure 2). However, the recording of postnatal depression levelled off after the first 3-4 months (Figure 2A). For all types of records, there were some clear peaks in recording immediately after delivery and in the period between 6 to 8 weeks after delivery coinciding with the time of postnatal maternal check-up consultation (Figure 2A).

There were 25,691 (12%) women with a record of antidepressant treatment. Women were predominantly prescribed SSRI (23,557 (92%)) with TCA (1,857 (7%)) and other (2,290 (8%)) prescriptions being much less common. Of the women who had an SSRI prescription, there were 31% who had a record of depression (Figure 1B), 31% who had a record of postnatal depression (Figure 1B), and 33% who had depression symptoms (Figure 1B). There were  6,270 (27%) women with SSRI prescription *without* a record of either the depression diagnoses or symptom within a year after delivery. However, 4,818 of these women had a record suggestive of depression or treatment *prior* to delivery leaving 1,452 (6%) on SSRI treatment without a record suggestive of depression.

There were 6,848 (3%) women with a record of referral for non-pharmacological treatment (Figure 1A). Of the women receiving non-pharmacological treatment, there were 24% who had a record of depression (Figure 1B), 22% who had a record of postnatal depression (Figure 1B), and 29% who had depression symptoms (Figure 2B), but 3,064 (45%) with no records indicating depression, postnatal depression or depressive symptoms. However, 2,041 of the the latter group of women had a record suggestive of depression or treatment *prior* to delivery leaving 1,023 (15%) with a referral for non-pharmacological treatment, but without a record of depression. Of those with non-pharmacological



For peer review only - http://bmjopen.bmj.com/site/about/guidelines.xhtml

treatment referral, 56% had SSRI prescription (Figure 1B), whereas conversely only 16% with a SSRI prescription had a record of non-pharmacological treatment referral (Figure 1B).

After the initial peak, the hazard for recording of postnatal depression and SSRI prescription show a markedly decreasing trend, while the other records show a relatively stable rate or slower decline (Figure 2).

There were 64,283 (31%) women who had at least one record suggestive of depression or treatment at any time prior to delivery. The prevalence of depression and SSRI treatment *after* delivery was high among these women. Thus, there were 9,666 (15%) with a record of depression or postnatal depression and 15,348 (24%) received SSRI treatment in the year after delivery. The figures were similar for women who have received SSRI treatment (n = 40,178, 19%) at any time prior delivery. Thus, there were 6,940 (17%) with a record of depression or postnatal depression and 11,595 (29%) received SSRI treatment in the year after delivery.

## Age, social deprivation and time

Younger women were much more likely to have a record of depressive diagnoses or symptoms compared to women aged 30 years or older. For example, women aged 15 – 19 years were nearly twice as likely to have a record of postnatal depression (RR, adjusted for social deprivation: 1.92 (1.76 to 2.10)) compared to women aged 30 – 34 years (Table 1). There were no marked differences for women above the age of 30 (Table 1). The pattern of SSRI treatment followed the same trends with nearly 1 in 5 women aged 15 – 19 receiving SSRI treatment in the first year after delivery (Table 2) while for those aged above 30 it was 1 in 10 (Table 2). Younger women were also more likely to receive non-pharmacological treatment than women aged 30 years or above (Table 2).

The time to the initiation of SSRI treatment after the delivery has become earlier in the more recent years (Figure 3). Thus, the initiation rate of SSRI treatment per 100 pregnancies (95% CI) at 8 weeks were 2.6 (2.5 to 2.8) in 2000-2004, increasing to 3.0 (2.9 to 3.1) in 2005-2009, and 3.8 (3.6 to 3.9) in 2010-2013. The overall rate of initiation of SSRI within the year after delivery, however, has not changed noticeably (Table 2). The rates of non-pharmacological treatment have increased from 2.4 (2.2 to 2.5) per 100 pregnancies in 2000-2004 to 3.8 (3.6 to 3.9) in 2010 – 2013 (Table 2). The recording of both depression diagnosis and postnatal diagnosis has decreased substantially over time while the recording of symptoms increased in the earlier time period, but have remained relatively constant since 2005 (Table 1).



For peer review only - http://bmjopen.bmj.com/site/about/guidelines.xhtml

The risk of having a record of depression, postnatal depression and depressive symptoms increased with increasing social deprivation (Table 1) and similar patterns were observed for both SSRI treatment and non-pharmacological treatment (Table 2). Thus, nearly 1 in 7 women from the most deprived areas received SSRI treatment within the first year after delivery in contrast to 1 in 11 women from the least deprived areas (Table 2). Supportive analyses suggest that the effect of age is, in general, stronger among the women *without* records suggestive of depression or treatment prior to delivery than among women *with* prior records (Appendix 1). However, the effect of social deprivation and calendar time was similar in women with and without prior records of depression or treatment (Appendix 1).

The women with early records (before 42 days after delivery) of depression, postnatal depression and depressive symptoms were more likely to have a prior record of depression or treatment (adjusted odds ratio estimates of 2.43 (2.02 to 2.94), 1.58 (1.41 to 1.77), and 1.55 (1.37 to 1.76), respectively) (Appendix 1 STable 5) and have delivered more recently (especially for postnatal depression and depressive symptoms; respective adjusted odds ratio estimates of 1.06 (0.87 to 1.28), 1.24 (1.08 to 1.42), and 1.65 (1.38 to 1.97) for the three records for the 2010-2013 calendar period against the baseline 2000-2005 period). The results were similar for women who had early records of SSRI treatment and non-pharmacological treatment (adjusted odds ratio estimates of 3.02 (2.78 to 3.29) and 1.91 (1.62 to 2.27) for the prior record, respectively, and of 1.59 (1.46 to 1.74) and 1.36 (1.11 to 1.68) for the recent time period).(Appendix 1 STable 6) No clear trends were observed in the effect of social deprivation or age group, except an indication of the youngest age group having a higher proportion of early recording for postnatal depression diagnosis (adjusted odds ratio estimate of 1.43 (1.17 to 1.75). (Appendix 1 STable 5).

## Discussion

We found that 11% of women who have given live birth had a record suggestive of depression in their primary care electronic health records within the first year after delivery. There were some peaks in recording of depressive diagnoses and symptoms and initiation of SSRI treatment soon after delivery (6 to 8 weeks), coinciding with the time of postnatal maternal check-up consultations although they continued to be recorded throughout the first year after delivery. The time to the initiation of SSRI treatment after the delivery has become earlier in the more recent years although the overall rate of initiation of SSRI within the year after delivery has not changed. Women with records suggestive of depression or SSRI treatment *prior* to delivery were more likely to have a subsequent records and/or treatment *after* delivery. Likewise, of women with records of depression and treatment after delivery



For peer review only - http://bmjopen.bmj.com/site/about/guidelines.xhtml

those with an *early* record (before 42 days after delivery) were more likely to have prior records of depression or treatments than women with *later* records (after 42 days after delivery).

Younger women were more likely to have a record suggestive of depression compared to women aged 30 years or older and the pattern of SSRI initiation followed the same trend with nearly 1 in 5 women aged between 15 – 19 years receiving SSRI treatment in the first year after delivery. The risk of depression increased with increasing social deprivation and similar patterns were observed for both SSRI treatment and non-pharmacological treatment.

## Strengths and limitations

A major strength of this study is that we have access to a very large sample of primary care electronic health records of women who gave live birth. These records reflect clinical practice in UK primary care and were made prospectively and therefore are not subject to recall bias. We considered a broad definition of depression based on clinical evaluation in the year after delivery as there are no specific guidelines to how it should be recorded in this period in primary care. Thus, we included women who had a specific diagnosis of postnatal depression as well women with records of depression diagnosis and symptoms, which may overestimate the number of women with postnatal depression compared to estimates based on a diagnostic interview and specific diagnostic instruments.

We are also aware that the indications for SSRI prescribing are broader than depression and some women in our study may have received SSRI for treatment for other indications for example anxiety. Yet, there is often an overlap between depression and anxiety (18) and we chose, therefore, to include initiation of all SSRI prescriptions in our study. Our estimates of referral for non-pharmacological treatment were relatively low. This may reflect a limited accessibility to non-pharmacological treatment, but it is also important to be aware that often in clinical practice the booking system for referrals is not directly linked to electronic health records and general practice staff will need to enter these referrals separately in the patient records. Furthermore, it is increasingly possible for women to self-refer themselves to psychological therapies through the 'Improving Access to Psychological Therapies' (IAPT) scheme in the UK (https://www.england.nhs.uk/mentalhealth/adults/iapt/). Therefore, it is likely that our study underestimates the actual referral rates for non-pharmacological treatments.

## Comparisons to existing evidence

Our summary estimate of postnatal depression, depression and symptoms of depression in the year after delivery (11%) was within the lower end of the range of previous prevalence estimates (10 – 19%). (2–4) Gavin et al estimated point prevalence of minor and major depression was highest in the third



For peer review only - http://bmjopen.bmj.com/site/about/guidelines.xhtml

month after delivery at 12.9%, although the confidence intervals were wide. (2) The results of our study suggest a peak in depression records and antidepressant treatment within 6 to 8 weeks after delivery, coinciding with the time of postnatal check-up consultations.

Our findings of increase in the use of symptoms codes as opposed to diagnostic codes for recording of depression reflect previous findings on recording of depression in primary care in general. (19) Rait et al suggest general practitioners' coding may be linked to the perceived severity of depression, with symptom codes being used for milder depression. Alternatively, this move towards recording of symptoms and less specific terms may be perceived as less stigmatising for individuals. (19)

Nearly 1 out 5 women in our study had a record suggestive of depression and/or SSRI treatment records prior to delivery. Of these women, 17% had additional records of depression and more than a quarter received SSRI treatment in the year after delivery. Prior depression has long been recognised as one of the strongest risk factors for depression in the year after delivery. (1–3,20) We also found that women who sought help early (before 42 days after delivery) were more likely to have had a prior record of depression or treatment. They might be better at recognising the symptoms earlier on than women without prior experience. Thus, a qualitative systematic review of help-seeking barriers by Dennis and Chung-Lee concluded that lack of knowledge about postpartum depression or the acceptance of myths was a significant help-seeking barrier and rendered mothers unable to recognise the symptoms of depression.(21)

Many women discontinue antidepressant treatment in pregnancy (22,23). A few studies suggest that these women are at higher risk of relapse (24) , but it is difficult to judge in observational settings and further research is needed to understand the role of antidepressant treatment in prevention of depression in the year after delivery.

Increased risk of postnatal depression among teenage mothers is well recognised with prevalence estimates as high as 26%. (25) Our study demonstrated that the level of recording of depressive diagnoses and symptoms continued to be higher for women right up to the age of 30, whereas no marked difference was found for women above the age of 30. Previous meta-analyses of postnatal depression have failed to recognise this 'L-shaped' difference in risk postnatal depression with age. (1,3) In contrast to our findings, a recent Canadian study on women aged 20 to 44 years based on the Canadian Community Health Survey suggests that there is a 'U-shaped' relationship with age and postnatal depression. Thus, they found that the prevalence of depression in women who had recently



For peer review only - http://bmjopen.bmj.com/site/about/guidelines.xhtml

delivered was significantly higher in women aged 40 to 44 years than in women aged 30 to 35 years (adjusted OR 3.72; 95% CI 2.15 to 6.41).(26)

There is some evidence that socioeconomic status is associated with prevalence of postnatal depression.(2,3,8,27) The results of a meta-regression analysis suggest that the prevalence of major depression is similar among socioeconomic status groups, but that minor depression may be more prevalent among lower socioeconomic status groups. (2) While we were unable to distinguish directly between diagnosis of major and minor depression we observed a clear gradient with increasing level of deprivation across all measures of depression and treatments. An even stronger socio-economic gradient in SSRI treatment was found among general population of adult women in UK. Hence, women from the most deprived areas were 64% more likely to have been initiated on SSRI treatment compared to women from the least deprived areas. (28)

Our study reflects women's primary care electronic health records. For women to have records of depression it requires that they have consulted their general practitioner. However, some women may be reluctant to seek help and unwilling to disclose or discuss their problem because of fear of stigma, negative perceptions of them as a mother or fear that their baby might be taken into care. (6,21,29) Investigators and clinicians should also be aware of the potential differences in the way women express postpartum depression and that it may differ for women of different educational backgrounds.(30) Likewise, some healthcare professionals may miss or misdiagnose postnatal depression in the period soon after birth (6) and estimates based on primary care health records may underestimate the 'true' prevalence of postnatal depression. Our study clearly shows that for many women depression and depressive symptoms were 'picked up' and treatment initiated at the time of the maternal check-up consultation in accordance to guidelines on antenatal and postnatal mental health care. (29) Yet, our results also revealed that depression is not limited to the immediate period after delivery and emphasises the need for health care professionals to be alert to signs and symptoms of depression throughout the first year after delivery. Indeed, a recent systematic review suggested that screening postpartum women for depression may reduce depressive symptoms in women with depression and reduce the prevalence of depression. (31)

## Conclusions

More than 1 in 10 women had electronic health records indicating depression or depressive symptoms within a year after delivery and more than 1 in 8 women received antidepressant treatment in this



For peer review only - http://bmjopen.bmj.com/site/about/guidelines.xhtml

period. Women aged below 30 and from the most deprived areas were at highest risk depression and most likely to receive antidepressant treatment.

## Author contributions

IP, TP, SH, KW and SK conceived the study, TP conducted the statistical analyses together with IP. IP and TP drafted the manuscript. All authors contributed to preparing the manuscript and have agreed to submit the final version of the manuscript. IP is the guarantor.

## Funding for this study

SH, IP and KW received funding from National Institute for Health Research (NIHR) school of primary care (grant 325). The views expressed in this publication are those of the author(s) and not necessarily those of the NHS, the National Institute for Health Research or the Department of Health. TP was funded by Academy of Finland (Finnish Centre of Excellence in Computational Inference Research Grant number 284642).

## Competing interest

None of the authors had competing interests.

## Data sharing

As the data for this study was bought under a licence no data are available for data sharing.

For peer review only

Figure legends

Figure 1. A. Numbers of records of depressive diagnoses and symptoms as well as treatment. B. Conditional frequency of records: given that one has the condition on the y-axis, what is the frequency of having the condition on x-axis. For example, the figure illustrates that 82% of those who had a diagnosis of depression also had a prescription of a SSRI. On the other hand, 31% of those who had a prescription of SSRI had a diagnosis of depression.D=Depression diagnosis, PND=Postnatal depression diagnosis, D/PND=either or both, D sym=Depression symptom, SSRI=SSRI prescription, NPT=Non-pharmacological treatment.

Figure 2. Cumulative incidences and smoothed hazards for the records. Six and eight weeks (6x7 and 8x7 days) are marked with a vertical grey line. Note the different y-axis scale for panels A and B. D=Depression, PND=Postnatal depression, SSRI=SSRI prescription, NPT=Non-pharmacological treatment.

Figure 3. Cumulative incidence of SSRI in three calendar periods. Six and eight weeks (6x7 and 8x7 days) are marked with a vertical grey line.

For peer review only



For peer review only - http://bmjopen.bmj.com/site/about/guidelines.xhtml

Table 1. Rates and Relative risk estimates of depression diagnosis, postnatal depression diagnosis and depression symptoms in the first year after delivery for 206,517 women who gave birth between 2000 and 2013. Adjusted by age group, calendar period, and Townsend deprivation

| | Depression diagnosis | | Postnatal depression diagnosis | | Depression symptom | |
|---|---|---|---|---|---|---|
| | Rate per 100 | Adjusted RR | Rate per 100 | Adjusted RR | Rate per 100 | Adjusted RR |
| Age group | | | | | | |
| 15-19 | 6.6 (6.1 to 7.1) | 1.64 (1.50 to 1.81) | 7.6 (7.1 to 8.1) | 1.92 (1.76 to 2.10) | 10.6 (10.0 to 11.2) | 2.10 (1.95 to 2.27) |
| 20-24 | 6.1 (5.9 to 6.4) | 1.59 (1.47 to 1.71) | 5.8 (5.5 to 6.0) | 1.49 (1.39 to 1.59) | 8.1 (7.7 to 8.4) | 1.63 (1.54 to 1.73) |
| 25-29 | 4.5 (4.3 to 4.7) | 1.22 (1.15 to 1.30) | 4.6 (4.4 to 4.8) | 1.21 (1.14 to 1.29) | 5.5 (5.3 to 5.7) | 1.18 (1.12 to 1.24) |
| 30-34 | 3.6 (3.4 to 3.7) | 1 | 3.8 (3.6 to 3.9) | 1 | 4.4 (4.3 to 4.6) | 1 |
| 35-39 | 3.5 (3.3 to 3.7) | 1.00 (0.93 to 1.06) | 3.5 (3.3 to 3.6) | 0.92 (0.86 to 0.98) | 4.3 (4.1 to 4.4) | 0.97 (0.92 to 1.02) |
| 40-49 | 3.1 (2.8 to 3.5) | 0.92 (0.81 to 1.03) | 3.2 (2.8 to 3.5) | 0.86 (0.77 to 0.97) | 4.7 (4.3 to 5.1) | 1.06 (0.96 to 1.17) |
| Calendar period | | | | | | |
| 2000-2004 | 5.7 (5.5 to 5.9) | 1 | 5.8 (5.6 to 6.0) | 1 | 4.4 (4.2 to 4.6) | 1 |
| 2005-2009 | 4.2 (4.0 to 4.3) | 0.71 (0.66 to 0.77) | 4.4 (4.2 to 4.5) | 0.73 (0.69 to 0.78) | 6.0 (5.8 to 6.2) | 1.31 (1.21 to 1.42) |
| 2010-2013 | 3.5 (3.3 to 3.6) | 0.58 (0.53 to 0.63) | 3.4 (3.3 to 3.5) | 0.56 (0.52 to 0.60) | 5.6 (5.4 to 5.8) | 1.21 (1.11 to 1.32) |
| Townsend deprivation index quintile | | | | | | |
| 1 | 3.2 (3.0 to 3.3) | 1 | 3.7 (3.5 to 3.9) | 1 | 4.1 (3.9 to 4.2) | 1 |
| 2 | 3.6 (3.4 to 3.8) | 1.14 (1.05 to 1.22) | 4.1 (3.9 to 4.2) | 1.09 (1.01 to 1.17) | 4.5 (4.3 to 4.7) | 1.07 (1.00 to 1.15) |
| 3 | 4.1 (3.9 to 4.3) | 1.26 (1.16 to 1.36) | 4.3 (4.1 to 4.5) | 1.12 (1.04 to 1.21) | 5.3 (5.1 to 5.5) | 1.19 (1.10 to 1.28) |
| 4 | 5.1 (4.9 to 5.3) | 1.51 (1.38 to 1.64) | 4.8 (4.6 to 5.0) | 1.20 (1.11 to 1.30) | 6.6 (6.4 to 6.9) | 1.42 (1.31 to 1.53) |
| 5 | 6.0 (5.7 to 6.3) | 1.69 (1.53 to 1.87) | 5.3 (5.0 to 5.5) | 1.26 (1.13 to 1.39) | 7.7 (7.4 to 8.0) | 1.56 (1.42 to 1.72) |

index. RR = relative risk.



For peer review only - http://bmjopen.bmj.com/site/about/guidelines.xhtml

Table 2. Rates and relative risk estimates of SSRI prescription and non-pharmacological treatment in the first year after delivery for 206,517

| | SSRI prescription | | Non-pharmacological treatment | |
|---|---|---|---|---|
| | Rate per 100 | Adjusted RR | Rate per 100 | Adjusted RR |
| Age group | | | | |
| 15-19 | 18.8 (18.0 to 19.5) | 1.78 (1.68 to 1.88) | 4.9 (4.5 to 5.4) | 1.55 (1.41 to 1.72) |
| 20-24 | 15.9 (15.5 to 16.4) | 1.54 (1.47 to 1.61) | 4.4 (4.1 to 4.6) | 1.38 (1.28 to 1.49) |
| 25-29 | 11.7 (11.5 to 12.0) | 1.18 (1.14 to 1.23) | 3.3 (3.2 to 3.5) | 1.11 (1.04 to 1.18) |
| 30-34 | 9.6 (9.4 to 9.8) | 1 | 2.9 (2.8 to 3.0) | 1 |
| 35-39 | 9.3 (9.1 to 9.6) | 0.99 (0.95 to 1.03) | 2.9 (2.7 to 3.0) | 0.99 (0.92 to 1.07) |
| 40-49 | 9.6 (9.0 to 10.1) | 1.01 (0.94 to 1.07) | 3.1 (2.7 to 3.4) | 1.05 (0.93 to 1.19) |
| Calendar period | | | | |
| 2000-2004 | 11.4 (11.1 to 11.7) | 1 | 2.4 (2.2 to 2.5) | 1 |
| 2005-2009 | 11.3 (11.1 to 11.6) | 0.97 (0.93 to 1.01) | 3.5 (3.3 to 3.6) | 1.43 (1.30 to 1.57) |
| 2010-2013 | 11.5 (11.2 to 11.7) | 0.96 (0.92 to 1.01) | 3.8 (3.6 to 3.9) | 1.54 (1.38 to 1.71) |
| Townsend deprivation index quantile | | | | |
| 1 | 8.9 (8.7 to 9.2) | 1 | 2.7 (2.5 to 2.8) | 1 |
| 2 | 10.0 (9.7 to 10.3) | 1.09 (1.04 to 1.14) | 3.0 (2.9 to 3.2) | 1.10 (1.00 to 1.20) |
| 3 | 11.3 (11.0 to 11.6) | 1.19 (1.14 to 1.25) | 3.3 (3.1 to 3.4) | 1.14 (1.04 to 1.25) |
| 4 | 13.1 (12.7 to 13.4) | 1.33 (1.25 to 1.40) | 3.8 (3.6 to 4.0) | 1.29 (1.17 to 1.42) |
| 5 | 15.2 (14.8 to 15.6) | 1.47 (1.38 to 1.57) | 4.2 (3.9 to 4.4) | 1.36 (1.22 to 1.52) |

women who gave birth between 2000 and 2013. Adjusted by age group, calendar period, and Townsend deprivation index. RR = relative risk.



For peer review only - http://bmjopen.bmj.com/site/about/guidelines.xhtml

## References

1. Beck CT. Predictors of postpartum depression: an update. Nurs Res. 2001 Oct;50(5):275–85.

2. Gavin NI, Gaynes BN, Lohr KN, Meltzer-Brody S, Gartlehner G, Swinson T. Perinatal depression: A systematic review of prevalence and incidence. Obstet Gynecol. 2005;106(5 I):1071–83.

3. O'hara MW, Swain AM. Rates and risk of postpartum depression—a meta-analysis. Int Rev Psychiatry. 1996 Jan;8(1):37–54.

4. Hahn-Holbrook J, Cornwell-Hinrichs T, Anaya I. Economic and Health Predictors of National Postpartum Depression Prevalence: A Systematic Review, Meta-analysis, and Meta-Regression of 291 Studies from 56 Countries. Front Psychiatry. 2017;8:248.

5. Bauer A, Parsonage M, Knapp M, Iemmi V, Adelaja B. Costs of perinatal mental health problems [Internet]. 2014 [cited 2015 Nov 6]. Available from: http://www.centreformentalhealth.org.uk/

6. Jones I, Shakespeare J. Postnatal depression. The BMJ. 2014 Aug 14;349:g4500.

7. Kingston D, Tough S, Whitfield H. Prenatal and postpartum maternal psychological distress and infant development: a systematic review. Child Psychiatry Hum Dev. 2012 Oct;43(5):683–714.

8. Robertson E, Grace S, Wallington T, Stewart DE. Antenatal risk factors for postpartum depression: a synthesis of recent literature. Gen Hosp Psychiatry. 2004 Aug;26(4):289–95.

9. Howard LM, Megnin-Viggars O, Symington I, Pilling S, On behalf of the Guideline Development Group. Antenatal and postnatal mental health: summary of updated NICE guidance. BMJ. 2014 Dec 18;349(dec18 5):g7394–g7394.

10. Stewart DE, Vigod S. Postpartum Depression. N Engl J Med. 2016 01;375(22):2177–86.

11. Chisholm J. The Read clinical classification. Br Med J. 1990 Apr 28;300(6732):1092.

12. Davé S, Petersen I. Creating medical and drug code lists to identify cases in primary care databases. Pharmacoepidemiol Drug Saf. 2009;18(8):704–7.

13. Townsend, Phillimore, Beattie A. Inequalities in health in the northern region. Newcastle upon Tyne: Northern Regional Health Authority and University of Bristol. University of Bristol; 1986.

14. Lis Y, Mann RD. The VAMP research multi-purpose database in the UK. J Clin Epidemiol. 1995 Mar;48(3):431–43.

For peer review only

15. Blak BT, Thompson M, Dattani H, Bourke A. Generalisability of The Health Improvement Network (THIN) database: demographics, chronic disease prevalence and mortality rates. Inform Prim Care. 2011;19(4):251–5.

16. Williams T, van Staa T, Puri S, Eaton S. Recent advances in the utility and use of the General Practice Research Database as an example of a UK Primary Care Data resource. Ther Adv Drug Saf. 2012 Apr;3(2):89–99.

17. Rasmussen CE, Williams CKI. Gaussian processes for machine learning. Cambridge, Mass: MIT Press; 2006. 248 p. (Adaptive computation and machine learning).

18. Falah-Hassani K, Shiri R, Dennis C-L. The prevalence of antenatal and postnatal co-morbid anxiety and depression: a meta-analysis. Psychol Med. 2017 Sep;47(12):2041–53.

19. Rait G, Walters K, Griffin M, Buszewicz M, Petersen I, Nazareth I. Recent trends in the incidence of recorded depression in primary care. Br J Psychiatry. 2009 Dec 1;195(6):520–4.

20. Milgrom J, Gemmill AW, Bilszta JL, Hayes B, Barnett B, Brooks J, et al. Antenatal risk factors for postnatal depression: a large prospective study. J Affect Disord. 2008 May;108(1-2):147–57.

21. Dennis C-L, Chung-Lee L. Postpartum Depression Help-Seeking Barriers and Maternal Treatment Preferences: A Qualitative Systematic Review. Birth. 2006 Dec;33(4):323–31.

22. Petersen I, Gilbert RE, Evans SJW, Man S-L, Nazareth I. Pregnancy as a major determinant for discontinuation of antidepressants: an analysis of data from The Health Improvement Network. J Clin Psychiatry. 2011 Jul;72(7):979–85.

23. Charlton R, Jordan S, Pierini A, Garne E, Neville A, Hansen A, et al. Selective serotonin reuptake inhibitor prescribing before, during and after pregnancy: a population-based study in six European regions. BJOG Int J Obstet Gynaecol. 2015 Jun;122(7):1010–20.

24. Cohen LS. Relapse of Major Depression During Pregnancy in Women Who Maintain or Discontinue Antidepressant Treatment. JAMA. 2006 Feb 1;295(5):499.

25. Troutman BR, Cutrona CE. Nonpsychotic postpartum depression among adolescent mothers. J Abnorm Psychol. 1990 Feb;99(1):69–78.

26. Muraca GM, Joseph KS. The association between maternal age and depression. J Obstet Gynaecol Can JOGC J Obstet Gynecol Can JOGC. 2014 Sep;36(9):803–10.

27. Hobfoll SE, Ritter C, Lavin J, Hulsizer MR, Cameron RP. Depression prevalence and incidence among inner-city pregnant and postpartum women. J Consult Clin Psychol. 1995 Jun;63(3):445–53.

For peer review only

28. McCrea RL, Sammon CJ, Nazareth I, Petersen I. Initiation and duration of selective serotonin reuptake inhibitor prescribing over time: UK cohort study. Br J Psychiatry. 2016 Nov 1;209(5):421–6.

29. National Institute for Health and Care Excellence (NICE). Antenatal and postnatal mental health: clinical management and service guidance [Internet]. 2014 Dec. Available from: http://guidance.nice.org.uk/cg185

30. Di Florio A, Putnam K, Altemus M, Apter G, Bergink V, Bilszta J, et al. The impact of education, country, race and ethnicity on the self-report of postpartum depression using the Edinburgh Postnatal Depression Scale. Psychol Med. 2017 Apr;47(5):787–99.

31. O'Connor E, Rossom RC, Henninger M, Groom HC, Burda BU. Primary Care Screening for and Treatment of Depression in Pregnant and Postpartum Women: Evidence Report and Systematic Review for the US Preventive Services Task Force. JAMA. 2016 Jan 26;315(4):388–406.

For peer review only



For peer review only - http://bmjopen.bmj.com/site/about/guidelines.xhtml

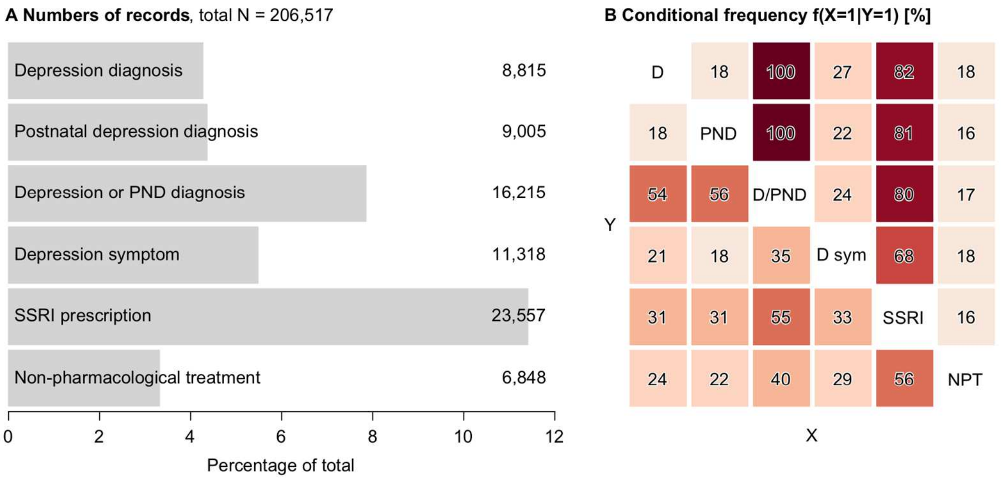


160x76mm (300 x 300 DPI)

For peer review only

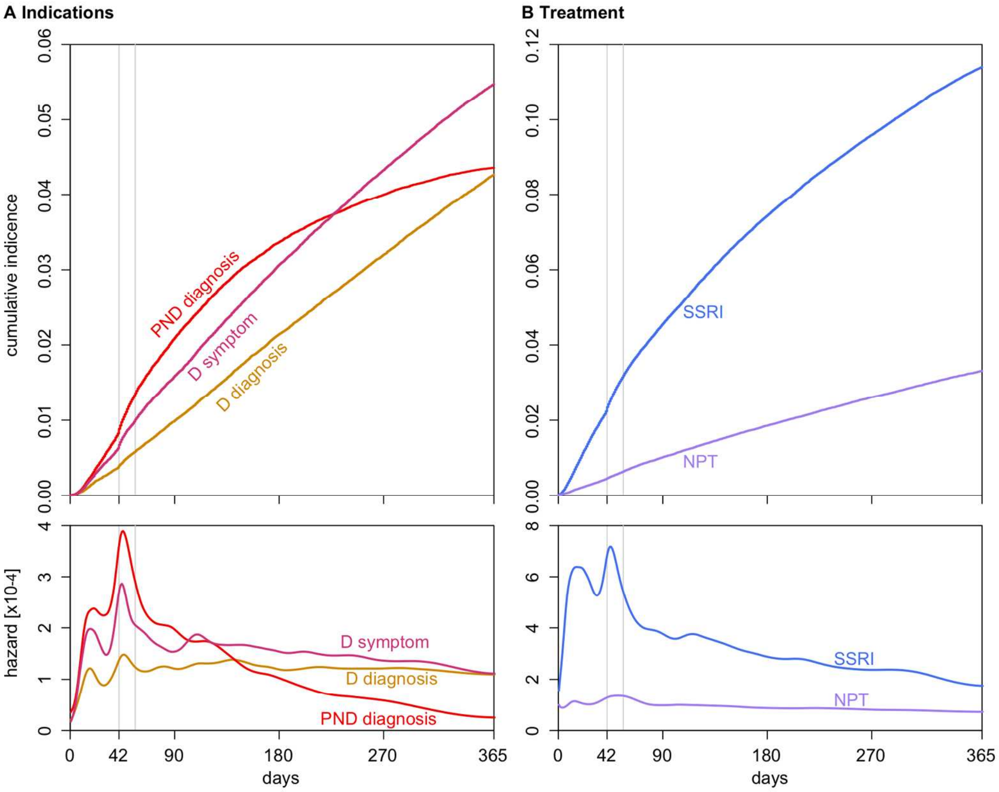


160x127mm (300 x 300 DPI)

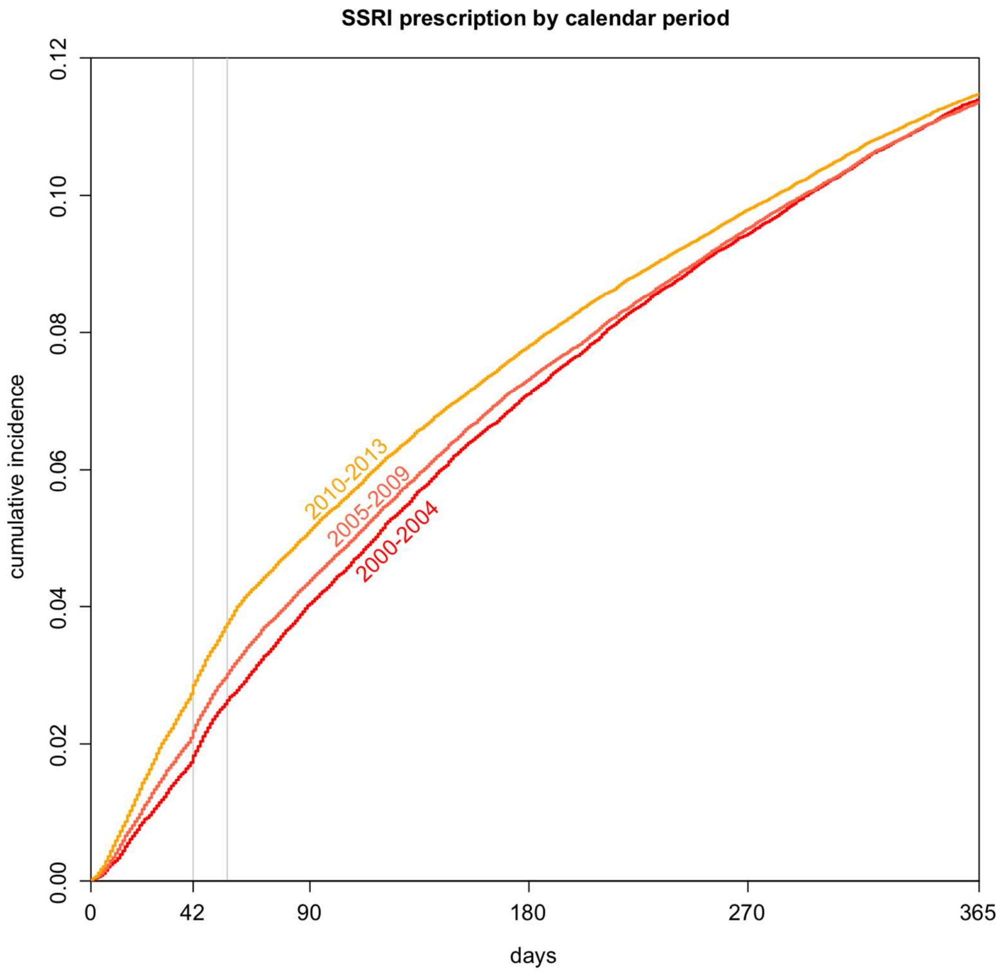


127x123mm (300 x 300 DPI)

STable 1. Rates and relative risk estimates of depression diagnosis, postnatal depression diagnosis and depression symptoms in the first year after delivery for 142,234 women who gave birth between 2000 and 2013 and had no prior record suggestive of depression. Adjusted by age group, calendar period, and Townsend deprivation index. RR = relative risk.

| | Depression diagnosis | | Postnatal depression diagnosis | | Depression symptom | |
|---|---|---|---|---|---|---|
| | Rate per 100 | Adjusted RR | Rate per 100 | Adjusted RR | Rate per 100 | Adjusted RR |
| Age group | | | | | | |
| 15-20 | 4.9 (4.4 to 5.3) | 2.74 (2.40 to 3.13) | 6.6 (6.0 to 7.1) | 2.88 (2.56 to 3.23) | 8.4 (7.8 to 9.0) | 3.35 (3.01 to 3.72) |
| 20-25 | 3.8 (3.5 to 4.0) | 2.21 (1.97 to 2.47) | 4.4 (4.1 to 4.7) | 1.98 (1.80 to 2.17) | 5.2 (4.9 to 5.6) | 2.13 (1.95 to 2.33) |
| 25-30 | 2.2 (2.0 to 2.4) | 1.33 (1.21 to 1.47) | 3.0 (2.8 to 3.2) | 1.33 (1.22 to 1.45) | 3.0 (2.8 to 3.1) | 1.26 (1.16 to 1.37) |
| 30-35 | 1.6 (1.5 to 1.7) | 1 | 2.3 (2.1 to 2.4) | 1 | 2.3 (2.1 to 2.4) | 1 |
| 35-40 | 1.4 (1.3 to 1.6) | 0.90 (0.81 to 1.01) | 2.0 (1.8 to 2.2) | 0.89 (0.80 to 0.98) | 2.0 (1.9 to 2.2) | 0.90 (0.82 to 0.99) |
| 40-50 | 1.2 (0.9 to 1.5) | 0.75 (0.59 to 0.95) | 1.8 (1.5 to 2.1) | 0.81 (0.67 to 0.99) | 1.8 (1.5 to 2.1) | 0.79 (0.64 to 0.96) |
| Calendar period | | | | | | |
| 2000-2004 | 3.0 (2.8 to 3.2) | 1 | 3.9 (3.7 to 4.1) | 1 | 2.6 (2.4 to 2.8) | 1 |
| 2005-2009 | 2.1 (2.0 to 2.2) | 0.67 (0.60 to 0.75) | 2.9 (2.7 to 3.0) | 0.70 (0.64 to 0.77) | 3.4 (3.3 to 3.6) | 1.27 (1.15 to 1.40) |
| 2010-2013 | 1.7 (1.6 to 1.8) | 0.53 (0.47 to 0.60) | 2.1 (2.0 to 2.3) | 0.52 (0.47 to 0.57) | 3.1 (2.9 to 3.2) | 1.13 (1.01 to 1.26) |
| Townsend deprivation index quantile | | | | | | |
| 1 | 1.6 (1.5 to 1.8) | 1 | 2.4 (2.2 to 2.5) | 1 | 2.3 (2.1 to 2.4) | 1 |
| 2 | 1.9 (1.7 to 2.0) | 1.11 (0.98 to 1.26) | 2.6 (2.4 to 2.8) | 1.06 (0.97 to 1.17) | 2.6 (2.4 to 2.7) | 1.05 (0.95 to 1.15) |
| 3 | 2.1 (2.0 to 2.3) | 1.19 (1.05 to 1.34) | 3.0 (2.8 to 3.2) | 1.17 (1.06 to 1.29) | 3.1 (2.9 to 3.3) | 1.18 (1.06 to 1.31) |
| 4 | 2.6 (2.4 to 2.8) | 1.34 (1.18 to 1.51) | 3.3 (3.1 to 3.5) | 1.16 (1.04 to 1.29) | 3.8 (3.6 to 4.0) | 1.28 (1.15 to 1.42) |
| 5 | 3.1 (2.8 to 3.3) | 1.42 (1.24 to 1.64) | 3.5 (3.2 to 3.7) | 1.10 (0.96 to 1.27) | 4.4 (4.1 to 4.7) | 1.32 (1.18 to 1.48) |

For peer review only

STable 2. Rates and relative risk estimates of SSRI prescription and non-pharmacological treatment in the first year after delivery for 142,234 women who gave birth between 2000 and 2013 and had no prior record suggestive of depression. Adjusted by age group, calendar period, and Townsend deprivation index. RR = relative risk.

| | SSRI prescription | | Non-pharmacological treatment | |
|---|---|---|---|---|
| | Rate per 100 | Adjusted RR | Rate per 100 | Adjusted RR |
| Age group | | | | |
| 15-20 | 14.2 (13.4 to 15.0) | 3.10 (2.85 to 3.37) | 3.4 (3.0 to 3.8) | 2.48 (2.12 to 2.90) |
| 20-25 | 9.9 (9.4 to 10.3) | 2.20 (2.06 to 2.36) | 2.2 (2.0 to 2.4) | 1.60 (1.40 to 1.84) |
| 25-30 | 5.8 (5.5 to 6.0) | 1.32 (1.24 to 1.41) | 1.6 (1.5 to 1.8) | 1.21 (1.08 to 1.36) |
| 30-35 | 4.3 (4.1 to 4.5) | 1 | 1.3 (1.2 to 1.4) | 1 |
| 35-40 | 3.7 (3.5 to 3.9) | 0.86 (0.80 to 0.93) | 1.3 (1.1 to 1.4) | 0.94 (0.83 to 1.07) |
| 40-50 | 3.1 (2.7 to 3.5) | 0.73 (0.63 to 0.84) | 1.2 (0.9 to 1.4) | 0.87 (0.69 to 1.11) |
| Calendar period | | | | |
| 2000-2004 | 6.5 (6.3 to 6.8) | 1 | 1.2 (1.1 to 1.3) | 1 |
| 2005-2009 | 5.8 (5.7 to 6.0) | 0.86 (0.81 to 0.91) | 1.8 (1.7 to 1.9) | 1.42 (1.25 to 1.62) |
| 2010-2013 | 5.1 (4.9 to 5.3) | 0.75 (0.70 to 0.80) | 1.7 (1.6 to 1.9) | 1.40 (1.22 to 1.61) |
| Townsend deprivation index quantile | | | | |
| 1 | 4.5 (4.3 to 4.7) | 1 | 1.4 (1.2 to 1.5) | 1 |
| 2 | 4.9 (4.7 to 5.2) | 1.05 (0.98 to 1.13) | 1.5 (1.4 to 1.6) | 1.06 (0.92 to 1.23) |
| 3 | 5.9 (5.6 to 6.1) | 1.15 (1.07 to 1.24) | 1.6 (1.5 to 1.8) | 1.08 (0.93 to 1.24) |
| 4 | 6.7 (6.4 to 7.0) | 1.18 (1.09 to 1.28) | 1.8 (1.7 to 2.0) | 1.12 (0.97 to 1.30) |
| 5 | 8.0 (7.6 to 8.4) | 1.26 (1.13 to 1.40) | 2.0 (1.8 to 2.2) | 1.12 (0.94 to 1.32) |

For peer review only

STable 3. Rates and relative risk estimates of depression diagnosis, postnatal depression diagnosis and depression symptoms in the first year after delivery for 64,283 women who gave birth between 2000 and 2013 and had a prior record suggestive of depression. Adjusted by age group, calendar period, and Townsend deprivation index. RR = relative risk.

| | Depression diagnosis | | Postnatal depression diagnosis | | Depression symptom | |
|---|---|---|---|---|---|---|
| | Rate per 100 | Adjusted RR | Rate per 100 | Adjusted RR | Rate per 100 | Adjusted RR |
| Age group | | | | | | |
| 15-20 | 12.6 (11.3 to 14.0) | 1.48 (1.31 to 1.67) | 11.1 (9.8 to 12.4) | 1.50 (1.32 to 1.71) | 18.3 (16.7 to 19.9) | 1.76 (1.58 to 1.95) |
| 20-25 | 10.6 (10.0 to 11.2) | 1.24 (1.15 to 1.35) | 8.2 (7.7 to 8.7) | 1.11 (1.02 to 1.21) | 13.3 (12.7 to 14.0) | 1.29 (1.22 to 1.38) |
| 25-30 | 9.2 (8.7 to 9.6) | 1.10 (1.03 to 1.17) | 7.9 (7.5 to 8.3) | 1.07 (0.99 to 1.15) | 10.6 (10.1 to 11.1) | 1.06 (0.99 to 1.12) |
| 30-35 | 8.3 (7.9 to 8.7) | 1 | 7.6 (7.2 to 8.0) | 1 | 9.8 (9.3 to 10.2) | 1 |
| 35-40 | 8.0 (7.6 to 8.5) | 0.98 (0.91 to 1.06) | 6.8 (6.3 to 7.2) | 0.90 (0.82 to 0.97) | 9.2 (8.7 to 9.7) | 0.95 (0.88 to 1.01) |
| 40-50 | 6.7 (5.8 to 7.5) | 0.84 (0.73 to 0.97) | 5.7 (4.9 to 6.5) | 0.78 (0.67 to 0.89) | 9.9 (8.9 to 11.0) | 1.02 (0.91 to 1.14) |
| Calendar period | | | | | | |
| 2000-2004 | 12.7 (12.1 to 13.2) | 1 | 10.8 (10.2 to 11.3) | 1 | 9.1 (8.7 to 9.6) | 1 |
| 2005-2009 | 8.8 (8.4 to 9.1) | 0.68 (0.63 to 0.74) | 7.7 (7.3 to 8.0) | 0.71 (0.66 to 0.77) | 11.7 (11.3 to 12.1) | 1.26 (1.16 to 1.38) |
| 2010-2013 | 7.0 (6.7 to 7.3) | 0.54 (0.50 to 0.59) | 5.9 (5.6 to 6.2) | 0.54 (0.50 to 0.59) | 10.6 (10.3 to 11.0) | 1.14 (1.03 to 1.25) |
| Townsend deprivation index quantile | | | | | | |
| 1 | 7.6 (7.2 to 8.1) | 1 | 7.6 (7.1 to 8.0) | 1 | 9.2 (8.7 to 9.7) | 1 |
| 2 | 8.1 (7.6 to 8.6) | 1.06 (0.98 to 1.16) | 7.8 (7.3 to 8.2) | 1.04 (0.95 to 1.13) | 9.5 (9.0 to 10.1) | 1.01 (0.93 to 1.11) |
| 3 | 8.5 (8.0 to 8.9) | 1.10 (1.01 to 1.20) | 7.1 (6.6 to 7.5) | 0.93 (0.85 to 1.03) | 10.0 (9.5 to 10.5) | 1.04 (0.95 to 1.13) |
| 4 | 9.7 (9.2 to 10.2) | 1.25 (1.15 to 1.36) | 7.7 (7.2 to 8.1) | 1.00 (0.92 to 1.09) | 11.9 (11.4 to 12.4) | 1.21 (1.11 to 1.32) |
| 5 | 10.6 (10.0 to 11.1) | 1.33 (1.20 to 1.47) | 8.2 (7.7 to 8.7) | 1.05 (0.94 to 1.16) | 13.0 (12.4 to 13.6) | 1.29 (1.16 to 1.43) |

For peer review only

STable 4. Rates and relative risk estimates of SSRI prescription and non-pharmacological treatment in the first year after delivery for 64,283 women who gave birth between 2000 and 2013 and had a prior record suggestive of depression. Adjusted by age group, calendar period, and Townsend deprivation index. RR = relative risk.

| | SSRI prescription | | Non-pharmacological treatment | |
|---|---|---|---|---|
| | Rate per 100 | Adjusted RR | Rate per 100 | Adjusted RR |
| Age group | | | | |
| 15-20 | 34.3 (32.4 to 36.3) | 1.47 (1.38 to 1.57) | 10.1 (8.8 to 11.3) | 1.42 (1.25 to 1.62) |
| 20-25 | 27.3 (26.4 to 28.1) | 1.18 (1.13 to 1.23) | 8.3 (7.8 to 8.9) | 1.18 (1.09 to 1.29) |
| 25-30 | 23.9 (23.2 to 24.5) | 1.04 (1.01 to 1.08) | 6.8 (6.4 to 7.2) | 0.99 (0.91 to 1.06) |
| 30-35 | 22.6 (22.0 to 23.2) | 1 | 6.8 (6.4 to 7.1) | 1 |
| 35-40 | 22.0 (21.2 to 22.7) | 0.98 (0.94 to 1.02) | 6.5 (6.0 to 6.9) | 0.95 (0.87 to 1.04) |
| 40-50 | 21.2 (19.8 to 22.6) | 0.94 (0.88 to 1.01) | 6.5 (5.7 to 7.4) | 0.95 (0.83 to 1.09) |
| | | | | |
| Calendar period | | | | |
| 2000-2004 | 24.0 (23.3 to 24.8) | 1 | 5.4 (5.0 to 5.8) | 1 |
| 2005-2009 | 23.6 (23.1 to 24.1) | 0.97 (0.93 to 1.02) | 7.3 (7.0 to 7.6) | 1.34 (1.21 to 1.49) |
| 2010-2013 | 24.1 (23.6 to 24.6) | 0.99 (0.95 to 1.03) | 7.8 (7.5 to 8.1) | 1.43 (1.27 to 1.61) |
| | | | | |
| Townsend deprivation index quantile | | | | |
| 1 | 21.7 (21.0 to 22.4) | 1 | 6.5 (6.0 to 6.9) | 1 |
| 2 | 22.7 (22.0 to 23.5) | 1.04 (0.99 to 1.09) | 6.8 (6.4 to 7.3) | 1.04 (0.93 to 1.16) |
| 3 | 23.2 (22.5 to 23.9) | 1.04 (0.99 to 1.10) | 6.9 (6.5 to 7.3) | 1.02 (0.92 to 1.14) |
| 4 | 25.0 (24.3 to 25.7) | 1.11 (1.06 to 1.16) | 7.5 (7.1 to 8.0) | 1.10 (0.99 to 1.23) |
| 5 | 26.6 (25.8 to 27.4) | 1.16 (1.10 to 1.22) | 7.6 (7.2 to 8.1) | 1.10 (0.97 to 1.25) |

For peer review only

STable 5. Rates of early recording (recording date within 42 days of delivery date) and odds ratio estimates of early vs. late recording (at or after 42 days) for depression diagnosis, postnatal depression diagnosis and depression symptoms in the first year after delivery for women who gave birth between 2000 and 2013. The values are calculated in datasets that only contain the women with the corresponding record within one year after delivery (number of women are given in the headers). Adjusted by age group, calendar period, Townsend deprivation index, and a prior record suggestive of depression. OR = odds ratio.

| | Depression diagnosis (N=8,815) | | Postnatal depression diagnosis (N=9,005) | | Depression symptom (N=11,318) | |
|---|---|---|---|---|---|---|
| | Rate per 100 | Adjusted OR | Rate per 100 | Adjusted OR | Rate per 100 | Adjusted OR |
| Age group | | | | | | |
| 15-20 | 8.6 (6.4 to 10.7) | 1.23 (0.89 to 1.67) | 23.3 (20.3 to 26.3) | 1.43 (1.17 to 1.75) | 12.5 (10.5 to 14.5) | 1.23 (0.98 to 1.53) |
| 20-25 | 8.1 (6.8 to 9.4) | 1.00 (0.80 to 1.26) | 19.2 (17.3 to 21.1) | 1.04 (0.88 to 1.22) | 12.9 (11.6 to 14.3) | 1.19 (1.00 to 1.41) |
| 25-30 | 8.6 (7.4 to 9.7) | 1.00 (0.82 to 1.24) | 17.6 (16.0 to 19.1) | 0.91 (0.79 to 1.06) | 10.8 (9.6 to 11.9) | 0.96 (0.81 to 1.14) |
| 30-35 | 8.5 (7.4 to 9.7) | 1 | 18.7 (17.2 to 20.3) | 1 | 10.9 (9.7 to 12.0) | 1 |
| 35-40 | 9.8 (8.3 to 11.4) | 1.14 (0.91 to 1.43) | 18.2 (16.2 to 20.2) | 0.95 (0.80 to 1.12) | 10.4 (9.0 to 11.8) | 0.95 (0.78 to 1.16) |
| 40-50 | 10.0 (6.6 to 13.4) | 1.12 (0.73 to 1.66) | 19.5 (15.0 to 23.9) | 1.03 (0.76 to 1.39) | 12.5 (9.4 to 15.6) | 1.08 (0.79 to 1.46) |
| Calendar period | | | | | | |
| 2000-2004 | 8.6 (7.5 to 9.6) | 1 | 17.6 (16.2 to 19.0) | 1 | 8.4 (7.2 to 9.5) | 1 |
| 2005-2009 | 8.4 (7.4 to 9.3) | 0.94 (0.79 to 1.13) | 18.0 (16.7 to 19.2) | 0.99 (0.87 to 1.13) | 10.9 (10.1 to 11.8) | 1.31 (1.10 to 1.57) |
| 2010-2013 | 9.4 (8.3 to 10.5) | 1.06 (0.87 to 1.28) | 21.6 (20.0 to 23.2) | 1.24 (1.08 to 1.42) | 13.6 (12.6 to 14.6) | 1.65 (1.38 to 1.97) |
| Townsend deprivation index quantile | | | | | | |
| 1 | 8.7 (7.2 to 10.1) | 1 | 19.3 (17.5 to 21.1) | 1 | 9.5 (8.2 to 10.8) | 1 |
| 2 | 9.0 (7.6 to 10.5) | 1.04 (0.81 to 1.34) | 16.2 (14.5 to 18.0) | 0.79 (0.66 to 0.94) | 10.6 (9.2 to 12.0) | 1.10 (0.89 to 1.36) |
| 3 | 8.3 (7.0 to 9.6) | 0.94 (0.74 to 1.21) | 18.2 (16.5 to 19.9) | 0.91 (0.77 to 1.07) | 12.5 (11.2 to 13.8) | 1.30 (1.07 to 1.59) |
| 4 | 8.7 (7.5 to 9.9) | 0.97 (0.77 to 1.24) | 21.2 (19.4 to 23.0) | 1.07 (0.91 to 1.26) | 11.9 (10.7 to 13.1) | 1.20 (0.99 to 1.46) |
| 5 | 9.0 (7.7 to 10.3) | 0.99 (0.78 to 1.27) | 19.0 (17.1 to 20.9) | 0.89 (0.75 to 1.06) | 12.0 (10.7 to 13.3) | 1.19 (0.98 to 1.46) |
| Any prior record suggestive of depression | | | | | | |
| No | 4.8 (4.0 to 5.5) | 1 | 15.4 (14.2 to 16.5) | 1 | 8.9 (8.0 to 9.7) | 1 |
| Yes | 10.8 (10.0 to 11.6) | 2.43 (2.02 to 2.94) | 21.8 (20.6 to 22.9) | 1.58 (1.41 to 1.77) | 13.0 (12.2 to 13.8) | 1.55 (1.37 to 1.76) |

For peer review only

STable 6. Rates of early recording (recording date within 42 days of delivery date) and odds ratio estimates of early vs. late recording (at or after 42 days) for SSRI prescription and non-pharmacological treatment in the first year after delivery for women who gave birth between 2000 and 2013. The values are calculated in datasets that only contain the women with the corresponding record within one year after delivery (number of women are given in the headers). Adjusted by age group, calendar period, Townsend deprivation index, and a prior record suggestive of depression. OR = odds ratio.

| | SSRI prescription (N=23,557) | | Non-pharmacological treatment (N=6,848) | |
|---|---|---|---|---|
| | Rate per 100 | Adjusted OR | Rate per 100 | Adjusted OR |
| Age group | | | | |
| 15-20 | 17.4 (15.7 to 19.1) | 0.99 (0.86 to 1.14) | 12.5 (9.6 to 15.4) | 1.01 (0.74 to 1.36) |
| 20-25 | 17.6 (16.5 to 18.7) | 0.83 (0.75 to 0.92) | 11.7 (9.9 to 13.4) | 0.82 (0.66 to 1.03) |
| 25-30 | 18.6 (17.7 to 19.6) | 0.85 (0.77 to 0.93) | 13.4 (11.7 to 15.0) | 0.96 (0.79 to 1.17) |
| 30-35 | 21.0 (20.0 to 22.0) | 1 | 13.8 (12.2 to 15.3) | 1 |
| 35-40 | 21.0 (19.7 to 22.3) | 0.96 (0.87 to 1.06) | 14.3 (12.3 to 16.3) | 1.03 (0.84 to 1.27) |
| 40-50 | 24.3 (21.5 to 27.1) | 1.07 (0.90 to 1.26) | 16.0 (11.8 to 20.2) | 1.13 (0.79 to 1.57) |
| | | | | |
| Calendar period | | | | |
| 2000-2004 | 15.2 (14.2 to 16.1) | 1 | 12.0 (10.1 to 13.8) | 1 |
| 2005-2009 | 18.4 (17.6 to 19.2) | 1.20 (1.09 to 1.31) | 11.4 (10.3 to 12.6) | 0.95 (0.77 to 1.17) |
| 2010-2013 | 23.7 (22.8 to 24.6) | 1.59 (1.46 to 1.74) | 16.0 (14.6 to 17.4) | 1.36 (1.11 to 1.68) |
| | | | | |
| Townsend deprivation index quantile | | | | |
| 1 | 18.7 (17.6 to 19.9) | 1 | 13.3 (11.5 to 15.2) | 1 |
| 2 | 19.1 (17.9 to 20.3) | 1.00 (0.90 to 1.12) | 12.8 (10.9 to 14.6) | 0.94 (0.74 to 1.19) |
| 3 | 19.3 (18.2 to 20.4) | 1.02 (0.92 to 1.14) | 14.0 (12.2 to 15.8) | 1.05 (0.84 to 1.31) |
| 4 | 20.4 (19.4 to 21.5) | 1.09 (0.98 to 1.21) | 13.4 (11.7 to 15.1) | 0.98 (0.79 to 1.23) |
| 5 | 20.0 (18.9 to 21.2) | 1.06 (0.95 to 1.18) | 13.3 (11.4 to 15.1) | 0.97 (0.77 to 1.23) |
| | | | | |
| Any prior record suggestive of depression | | | | |
| No | 9.6 (8.9 to 10.2) | 1 | 8.8 (7.6 to 9.9) | 1 |
| Yes | 24.9 (24.2 to 25.6) | 3.02 (2.78 to 3.29) | 15.7 (14.7 to 16.8) | 1.91 (1.62 to 2.27) |

For peer review only

**STROBE 2007 (v4) Statement—Checklist of items that should be included in reports of *cohort studies***

| Section/Topic | Item # | Recommendation | Reported on page # |
|---|---|---|---|
| **Title and abstract** | 1 | (*a*) Indicate the study's design with a commonly used term in the title or the abstract | 1 |
| | | (*b*) Provide in the abstract an informative and balanced summary of what was done and what was found | 2 |
| **Introduction** | | | |
| Background/rationale | 2 | Explain the scientific background and rationale for the investigation being reported | 4 |
| Objectives | 3 | State specific objectives, including any prespecified hypotheses | 4 |
| **Methods** | | | |
| Study design | 4 | Present key elements of study design early in the paper | 5 |
| Setting | 5 | Describe the setting, locations, and relevant dates, including periods of recruitment, exposure, follow-up, and data collection | 5 |
| Participants | 6 | (*a*) Give the eligibility criteria, and the sources and methods of selection of participants. Describe methods of follow-up | 5 |
| | | (*b*) For matched studies, give matching criteria and number of exposed and unexposed | N/A |
| Variables | 7 | Clearly define all outcomes, exposures, predictors, potential confounders, and effect modifiers. Give diagnostic criteria, if applicable | 5 |
| Data sources/ measurement | 8* | For each variable of interest, give sources of data and details of methods of assessment (measurement). Describe comparability of assessment methods if there is more than one group | 5 |
| Bias | 9 | Describe any efforts to address potential sources of bias | 6 |
| Study size | 10 | Explain how the study size was arrived at | 5 |
| Quantitative variables | 11 | Explain how quantitative variables were handled in the analyses. If applicable, describe which groupings were chosen and why | 6 |
| Statistical methods | 12 | (*a*) Describe all statistical methods, including those used to control for confounding | 6 |
| | | (*b*) Describe any methods used to examine subgroups and interactions | 6 |
| | | (*c*) Explain how missing data were addressed | 6 |
| | | (*d*) If applicable, explain how loss to follow-up was addressed | 6 |
| | | (*e*) Describe any sensitivity analyses | 6 |
| **Results** | | | |

For peer review only

| | | | |
|---|---|---|---|
| Participants | 13* | (a) Report numbers of individuals at each stage of study—eg numbers potentially eligible, examined for eligibility, confirmed eligible, included in the study, completing follow-up, and analysed | 7 |
| | | (b) Give reasons for non-participation at each stage | N/A |
| | | (c) Consider use of a flow diagram | N/A |
| Descriptive data | 14* | (a) Give characteristics of study participants (eg demographic, clinical, social) and information on exposures and potential confounders | 7 |
| | | (b) Indicate number of participants with missing data for each variable of interest | N/A |
| | | (c) Summarise follow-up time (eg, average and total amount) | 7 |
| Outcome data | 15* | Report numbers of outcome events or summary measures over time | 7 |
| Main results | 16 | (*a*) Give unadjusted estimates and, if applicable, confounder-adjusted estimates and their precision (eg, 95% confidence interval). Make clear which confounders were adjusted for and why they were included | 8 |
| | | (*b*) Report category boundaries when continuous variables were categorized | 8 |
| | | (*c*) If relevant, consider translating estimates of relative risk into absolute risk for a meaningful time period | 8 |
| Other analyses | 17 | Report other analyses done—eg analyses of subgroups and interactions, and sensitivity analyses | 8-9 |
| **Discussion** | | | |
| Key results | 18 | Summarise key results with reference to study objectives | 9 |
| **Limitations** | | | |
| Interpretation | 20 | Give a cautious overall interpretation of results considering objectives, limitations, multiplicity of analyses, results from similar studies, and other relevant evidence | 10 |
| Generalisability | 21 | Discuss the generalisability (external validity) of the study results | 10 |
| **Other information** | | | |
| Funding | 22 | Give the source of funding and the role of the funders for the present study and, if applicable, for the original study on which the present article is based | 12 |

*Give information separately for cases and controls in case-control studies and, if applicable, for exposed and unexposed groups in cohort and cross-sectional studies.

**Note:** An Explanation and Elaboration article discusses each checklist item and gives methodological background and published examples of transparent reporting. The STROBE checklist is best used in conjunction with this article (freely available on the Web sites of PLoS Medicine at http://www.plosmedicine.org/, Annals of Internal Medicine at http://www.annals.org/, and Epidemiology at http://www.epidem.com/). Information on the STROBE Initiative is available at www.strobe-statement.org.

For peer review only

BMJ Open

# Depression, depressive symptoms and treatments in women who have recently given birth: UK cohort study

| | |
|---|---|
| Journal: | *BMJ Open* |
| Manuscript ID | bmjopen-2018-022152.R2 |
| Article Type: | Research |
| Date Submitted by the Author: | 29-Aug-2018 |
| Complete List of Authors: | Petersen, Irene; University College London Research Department of Primary Care and Population Health<br>Peltola, Tomi; Aalto University, Helsinki Institute for Information Technology HIIT<br>Kaski, Samuel; Aalto University, Helsinki Institute for Information Technology HIIT<br>Walters, Kate; University College London Research Department of Primary Care and Population Health<br>Hardoon, Sarah; University College London Research Department of Primary Care and Population Health |
| <b>Primary Subject Heading</b>: | Mental health |
| Secondary Subject Heading: | General practice / Family practice, Patient-centred medicine |
| Keywords: | postnatal depression, SSRI treatment, non-pharmacological treatment, PRIMARY CARE, Depression & mood disorders < PSYCHIATRY |
| | |

SCHOLARONE™
Manuscripts

# Depression, depressive symptoms and treatments in women who have recently given birth: UK cohort study

Irene Petersen, PhD (1, 2), Tomi Peltola, PhD (1,3), Samuel Kaski, PhD (3), Kate Walters, PhD (1) and Sarah Hardoon, PhD (1)

Affiliations:

1) Department of Primary Care and Population Health, University College London, Rowland Hill St, London NW3 2PF, UK

2) Department of Clinical Epidemiology, Aarhus University, Olof Palmes Alle 43 – 45, 8200 Aarhus N, Denmark

3) Helsinki Institute for Information Technology HIIT, Department of Computer Science, Aalto University, Konemiehentie 2, 02150 Espoo, Finland

Corresponding author: Irene Petersen, e-mail: i.petersen@ucl.ac.uk, Department of Primary Care and Population Health, UCL, Rowland Hill St, London NW3 2PF, UK.

Word count: 3387

For peer review only



For peer review only - http://bmjopen.bmj.com/site/about/guidelines.xhtml

## Abstract

Objectives: To investigate how depression is recognised in the year after child birth and treatment given in clinical practice.

Design: Cohort study based on UK primary care electronic health records.

Setting: Primary Care

Participants: Women who have given live birth between 2000 and 2013.

Outcomes: Prevalence of postnatal depression, depression diagnoses, depressive symptoms, antidepressant and non-pharmacological treatment within a year after birth.

Results: Of 206,517 women 23,623 (11%) had a record of depressive diagnosis or symptoms in the year after delivery and more than 1 in 8 women received antidepressant treatment. Recording and treatment peaked 6 to 8 weeks after delivery. Initiation of SSRI treatment has become earlier in the more recent years. Thus, the initiation rate of SSRI treatment per 100 pregnancies (95% CI) at 8 weeks were 2.6 (2.5 to 2.8) in 2000-2004, increasing to 3.0 (2.9 to 3.1) in 2005-2009, and 3.8 (3.6 to 3.9) in 2010-2013. The overall rate of initiation of SSRI within the year after delivery, however, has not changed noticeably. A third of the women had at least one record suggestive of depression at any time prior to delivery and of these 1 in 4 received SSRI treatment in the year after delivery.

Younger women were most likely to have records of depression and depressive symptoms. Relative Risk for postnatal depression: Age 15 – 19: 1.92 (1.76 to 2.10), Age 20 – 24: 1.49 (1.39 to 1.59) versus Age 30 – 34)). The risk of depression, postnatal depression and depressive symptoms increased with increasing social deprivation.

Conclusions: More than 1 in 10 women had electronic health records indicating depression diagnoses or depressive symptoms within a year after delivery and more than 1 in 8 women received antidepressant treatment in this period. Women aged below 30 and from the most deprived areas were at highest risk depression and most likely to receive antidepressant treatment.

## Summary

Strength and limitations of this study



For peer review only - http://bmjopen.bmj.com/site/about/guidelines.xhtml

- A major strength of this study is that we have access to a very large sample of primary care electronic health records of women who gave live birth.
- These records reflect clinical practice in UK primary care and were made prospectively.
- We considered a broad definition of depression on clinical evaluation in the year after delivery as there are no specific guidelines to how it should be recorded.
- This study may overestimate the number of women with postnatal depression compared to estimates based on a diagnostic interview and specific diagnostic instruments.
- Non-pharmacological treatment may not be well recorded in primary care electronic health records.

For peer review only



For peer review only - http://bmjopen.bmj.com/site/about/guidelines.xhtml

# Introduction

Many women experience depression in the year after they have given birth. Postnatal depression affects an estimated 10 – 19% of women, although the estimates vary substantially between countries and settings. (1–4) Depression may have severe consequences for the mother and, in turn, have physical, cognitive, and emotional effects on their children's development, continuing into later life. (5–8) A report published by the London School of Economics estimated that perinatal depression, anxiety and psychosis carry a total long-term cost to society of about £6.6 billion for each one-year cohort of births in the UK. (5) This is equivalent to a cost of just under £10,000 for every single birth in the country. Nearly three-quarters (72%) of this cost relates to adverse impacts on the child rather than the mother. (5)

Guidelines in both US and UK on antenatal and postnatal mental health recommend that health care professionals should consider asking simple screening questions about current and past histories of depression, anxiety, alcohol and illicit drug use as part of a general discussion about mental health and wellbeing in pregnancy and the perinatal period. (9,10) However, very limited information is available on when depression is recognised and how it is treated in clinical practice in the year after women have given birth. For most women who experience depression in this period primary care physicians would be a first point of contact. In this study, we sought to obtain an overview of actual clinical practice in UK primary care by examining electronic health records on more than 200,000 women who have given life birth between 2000 and 2013. We followed the women for a year after delivery and our aim was to examine how and when depression and depressive symptoms were recorded and treatment provided in general practice and the interrelation between antidepressant and non-pharmacological treatment.



For peer review only - http://bmjopen.bmj.com/site/about/guidelines.xhtml

# Methods

## Data source

We used data from The Health Improvement Network (THIN). This is a large primary care database that provides anonymised longitudinal general practice (family practice) data on patients' clinical and prescribing records and includes data from around 6% of the United Kingdom population. Diagnoses and symptoms are recorded by practice staff using Read codes, which is a hierarchical coding system including more than 100,000 codes. (11,12) The Read code system can be mapped to ICD-10, but in addition the Read codes include a number of symptoms and administrative codes. (12) Prescriptions are issued electronically and directly recorded on the general practice computer systems. In addition, the database holds individual patient level information about year of birth, date of registration, date of death and transfer out of the practice and information about social deprivation (quintiles of Townsend deprivation scores). The Townsend scores is based on census data (2011) for car ownership, owner-occupation, overcrowding and unemployment in a patient's postcode. (13)

Over 98% of the UK population are registered with a general practitioner (GP) (14) and the UK primary care databases are broadly representative of the United Kingdom population. (15,16) While perinatal care is often shared between general practice staff and midwives, the GP remains responsible for women's general medical care including continued prescribing of medicines such as antidepressants. Some women may also receive care from local National Health Service (NHS) mental health trusts, but trusts have limited prescribing budgets and for most women prescribing of psychotropic medication remains with the GP. Furthermore, after a few weeks after delivery the care by the midwife ends and general practitioners are the first point of contact. Typically, women will consult their general practitioner for a postnatal maternal check-up at 6 to 8 weeks after delivery.

## Study population

We utilised data from women who have given live birth between 1st January 2000 and 31st December 2013 and who were permanently registered with the same general practice for at least one year after delivery. As some women had more than one pregnancy and the risk of postnatal depression may be strongly correlated within women we randomly selected one pregnancy per woman for our analyses.

## Variables

We identified women with one or more records entered as a Read code in their primary care electronic health records which suggested they had depression, postnatal depression or symptoms of depression



For peer review only - http://bmjopen.bmj.com/site/about/guidelines.xhtml

as well women on antidepressant and non-pharmacological treatment (referral to counselling and psychotherapy) in the year after they have given birth. Antidepressant treatment was classified as selective serotonin reuptake inhibitors (SSRI), TCA and other antidepressants. For TCA we only considered treatment that was prescribed above treatment threshold for depression, as lower doses may be prescribed for other reasons such as chronic pain. In addition, we included information on calendar year of delivery, age at delivery and social deprivation.

## Data analysis

First, we estimated the prevalence of any records directly suggestive of depression (postnatal depression, depression diagnoses, depressive symptoms) as well as separate estimates for postnatal depression, depression diagnoses, depressive symptoms, antidepressant or non-pharmacological treatments within a year after giving birth. These estimates are reported in Figure 1A. We then estimated how the records were interrelated. Interrelations were reported as conditional frequencies, that is, the frequency of having a record of X given that one has a record of Y. These frequencies are reported in Figure 1B. For example, the figure illustrates that 82% of those who had a diagnosis of depression also had a prescription of a SSRI. On the other hand, 31% of those who had a prescription of SSRI had a diagnosis of depression.

We estimated the timing of the recording within the follow-up year and report cumulative incidence curves (as one minus the Kaplan-Meier estimate). We also estimated smoothed daily hazards using a Gaussian process model (17) to visualize the daily changes in the timing of recording.

For each of the three depression outcomes (depression diagnosis, postnatal depression diagnosis and depression symptoms) and for SSRI and non-pharmacological treatments, we used Poisson regression to model relative risks of having a record associated with age, calendar time and social deprivation (Townsend scores). Age was split into six age groups (15-19, 20-24, 25-29, 30-34, 35-39, 40-49) and calendar time into three periods (2000-2004, 2005-2009, 2010-2013). 95% CI were computed using modified Poisson regression accounting for the clustering of women in general practices. We conducted supportive analyses stratified 1) on whether women had any record suggestive of depression or treatment prior to delivery. 2) on whether the women had early or late records of depression or treatment. In the latter analyses we categorised women into two groups; women who had a record of depression or treatments before 42 days after delivery were considered as having an *early* record and women who had a record of depression or treatments after 42 days of delivery were considered as



For peer review only - http://bmjopen.bmj.com/site/about/guidelines.xhtml

having a *late* record. We investigated whether this was associated with age, social deprivation, calendar time and any record suggestive of depression or treatment prior to delivery using logistic regression.

## Ethics

The scheme for THIN to obtain and provide anonymous patient data to researchers was approved by the National Health Service South-East Multicenter Research Ethics Committee (MREC) in 2002 and scientific approval for this study was obtained from IMS Scientific Review Committee.

## Patient and Public Involvement

Charlotte Walker, who is a mental health service user, has been involved with the original design of the study proposal and provided feedback on this manuscript and thus helped to shape the discussion of the paper from a service user's perspective.

For peer review only



For peer review only - http://bmjopen.bmj.com/site/about/guidelines.xhtml

## Results

In total, 206,517 women were included in the study and there were 23,623 (11%) with at least one record directly suggestive of depression (depression, postnatal depression or symptoms of depression) in the year after delivery. Of these women, there were 4% with a record of depression, 4% with a record of postnatal depression and 5% with symptoms of depression (Figure 1A). Of those women with a depression diagnosis, 2,349/8,815 (27%) also had depressive symptoms (Figure 1B), and of those with postnatal depression diagnosis, 2,005/9,005(22%) also had depressive symptoms (Figure 1B). In contrast, there were 7,408/11,318 (65%) women with a record of depressive symptoms *without* either a depression diagnosis or postnatal depression diagnosis.

The number of women with a record suggestive of depression continued to rise throughout the first year after delivery (Figure 2). However, the recording of postnatal depression levelled off after the first 3-4 months (Figure 2A). For all types of records, there were some clear peaks in recording immediately after delivery and in the period between 6 to 8 weeks after delivery coinciding with the time of postnatal maternal check-up consultation (Figure 2A).

There were 25,691 (12%) women with a record of antidepressant treatment. Women were predominantly prescribed SSRI (23,557 (92%)) with TCA (1,857 (7%)) and other (2,290 (8%)) prescriptions being much less common. Of the women who had an SSRI prescription, there were 31% who had a record of depression (Figure 1B), 31% who had a record of postnatal depression (Figure 1B), and 33% who had depression symptoms (Figure 1B). There were 6,270 (27%) women with SSRI prescription *without* a record of either the depression diagnoses or symptom within a year after delivery. However, 4,818 of these women had a record suggestive of depression or treatment *prior* to delivery leaving 1,452 (6%) on SSRI treatment without a record suggestive of depression.

There were 6,848 (3%) women with a record of referral for non-pharmacological treatment (Figure 1A). Of the women receiving non-pharmacological treatment, there were 24% who had a record of depression (Figure 1B), 22% who had a record of postnatal depression (Figure 1B), and 29% who had depression symptoms (Figure 2B), but 3,064 (45%) with no records indicating depression, postnatal depression or depressive symptoms. However, 2,041 of the the latter group of women had a record suggestive of depression or treatment *prior* to delivery leaving 1,023 (15%) with a referral for non-pharmacological treatment, but without a record of depression. Of those with non-pharmacological



For peer review only - http://bmjopen.bmj.com/site/about/guidelines.xhtml

treatment referral, 56% had SSRI prescription (Figure 1B), whereas conversely only 16% with a SSRI prescription had a record of non-pharmacological treatment referral (Figure 1B).

After the initial peak, the hazard for recording of postnatal depression and SSRI prescription show a markedly decreasing trend, while the other records show a relatively stable rate or slower decline (Figure 2).

There were 64,283 (31%) women who had at least one record suggestive of depression or treatment at any time prior to delivery. The prevalence of depression and SSRI treatment *after* delivery was high among these women. Thus, there were 9,666 (15%) with a record of depression or postnatal depression and 15,348 (24%) received SSRI treatment in the year after delivery. The figures were similar for women who have received SSRI treatment (n = 40,178, 19%) at any time prior delivery. Thus, there were 6,940 (17%) with a record of depression or postnatal depression and 11,595 (29%) received SSRI treatment in the year after delivery.

## Age, social deprivation and time

Younger women were much more likely to have a record of depressive diagnoses or symptoms compared to women aged 30 years or older. For example, women aged 15 – 19 years were nearly twice as likely to have a record of postnatal depression (RR, adjusted for social deprivation: 1.92 (1.76 to 2.10)) compared to women aged 30 – 34 years (Table 1). There were no marked differences for women above the age of 30 (Table 1). The pattern of SSRI treatment followed the same trends with nearly 1 in 5 women aged 15 – 19 receiving SSRI treatment in the first year after delivery (Table 2) while for those aged above 30 it was 1 in 10 (Table 2). Younger women were also more likely to receive non-pharmacological treatment than women aged 30 years or above (Table 2).

The time to the initiation of SSRI treatment after the delivery has become earlier in the more recent years (Figure 3). Thus, the initiation rate of SSRI treatment per 100 pregnancies (95% CI) at 8 weeks were 2.6 (2.5 to 2.8) in 2000-2004, increasing to 3.0 (2.9 to 3.1) in 2005-2009, and 3.8 (3.6 to 3.9) in 2010-2013. The overall rate of initiation of SSRI within the year after delivery, however, has not changed noticeably (Table 2). The rates of non-pharmacological treatment have increased from 2.4 (2.2 to 2.5) per 100 pregnancies in 2000-2004 to 3.8 (3.6 to 3.9) in 2010 – 2013 (Table 2). The recording of both depression diagnosis and postnatal diagnosis has decreased substantially over time while the recording of symptoms increased in the earlier time period, but have remained relatively constant since 2005 (Table 1).



For peer review only - http://bmjopen.bmj.com/site/about/guidelines.xhtml

The risk of having a record of depression, postnatal depression and depressive symptoms increased with increasing social deprivation (Table 1) and similar patterns were observed for both SSRI treatment and non-pharmacological treatment (Table 2). Thus, nearly 1 in 7 women from the most deprived areas received SSRI treatment within the first year after delivery in contrast to 1 in 11 women from the least deprived areas (Table 2). Supportive analyses suggest that the effect of age is, in general, stronger among the women *without* records suggestive of depression or treatment prior to delivery than among women *with* prior records (Appendix 1 STable 1-4 ). However, the effect of social deprivation and calendar time was similar in women with and without prior records of depression or treatment (Appendix 1 STable 1-4).

The women with early records (before 42 days after delivery) of depression, postnatal depression and depressive symptoms were more likely to have a prior record of depression or treatment (adjusted odds ratio estimates of 2.43 (2.02 to 2.94), 1.58 (1.41 to 1.77), and 1.55 (1.37 to 1.76), respectively) (Appendix 1 STable 5) and have delivered more recently (especially for postnatal depression and depressive symptoms; respective adjusted odds ratio estimates of 1.06 (0.87 to 1.28), 1.24 (1.08 to 1.42), and 1.65 (1.38 to 1.97) for the three records for the 2010-2013 calendar period against the baseline 2000-2005 period). The results were similar for women who had early records of SSRI treatment and non-pharmacological treatment (adjusted odds ratio estimates of 3.02 (2.78 to 3.29) and 1.91 (1.62 to 2.27) for the prior record, respectively, and of 1.59 (1.46 to 1.74) and 1.36 (1.11 to 1.68) for the recent time period).(Appendix 1 STable 6) No clear trends were observed in the effect of social deprivation or age group, except an indication of the youngest age group having a higher proportion of early recording for postnatal depression diagnosis (adjusted odds ratio estimate of 1.43 (1.17 to 1.75). (Appendix 1 STable 5).

## Discussion

We found that 11% of women who have given live birth had a record suggestive of depression in their primary care electronic health records within the first year after delivery. There were some peaks in recording of depressive diagnoses and symptoms and initiation of SSRI treatment soon after delivery (6 to 8 weeks), coinciding with the time of postnatal maternal check-up consultations although they continued to be recorded throughout the first year after delivery. The time to the initiation of SSRI treatment after the delivery has become earlier in the more recent years although the overall rate of initiation of SSRI within the year after delivery has not changed. Women with records suggestive of depression or SSRI treatment *prior* to delivery were more likely to have a subsequent records and/or



For peer review only - http://bmjopen.bmj.com/site/about/guidelines.xhtml

treatment *after* delivery. Likewise, of women with records of depression and treatment after delivery those with an *early* record (before 42 days after delivery) were more likely to have prior records of depression or treatments than women with *later* records (after 42 days after delivery).

Younger women were more likely to have a record suggestive of depression compared to women aged 30 years or older and the pattern of SSRI initiation followed the same trend with nearly 1 in 5 women aged between 15 – 19 years receiving SSRI treatment in the first year after delivery. The risk of depression increased with increasing social deprivation and similar patterns were observed for both SSRI treatment and non-pharmacological treatment.

## Strengths and limitations

A major strength of this study is that we have access to a very large sample of primary care electronic health records of women who gave live birth. These records reflect clinical practice in UK primary care and were made prospectively and therefore are not subject to recall bias. We considered a broad definition of depression based on clinical evaluation in the year after delivery as there are no specific guidelines to how it should be recorded in this period in primary care. Thus, we included women who had a specific diagnosis of postnatal depression as well women with records of depression diagnosis and symptoms, which may overestimate the number of women with postnatal depression compared to estimates based on a diagnostic interview and specific diagnostic instruments.

We are also aware that the indications for SSRI prescribing are broader than depression and some women in our study may have received SSRI for treatment for other indications for example anxiety. Yet, there is often an overlap between depression and anxiety (18) and we chose, therefore, to include initiation of all SSRI prescriptions in our study. Our estimates of referral for non-pharmacological treatment were relatively low. This may reflect a limited accessibility to non-pharmacological treatment, but it is also important to be aware that often in clinical practice the booking system for referrals is not directly linked to electronic health records and general practice staff will need to enter these referrals separately in the patient records. Furthermore, it is increasingly possible for women to self-refer themselves to psychological therapies through the 'Improving Access to Psychological Therapies' (IAPT) scheme in the UK (https://www.england.nhs.uk/mentalhealth/adults/iapt/). Therefore, it is likely that our study underestimates the actual referral rates for non-pharmacological treatments.

## Comparisons to existing evidence

Our summary estimate of postnatal depression, depression and symptoms of depression in the year after delivery (11%) was within the lower end of the range of previous prevalence estimates (10 – 19%).



For peer review only - http://bmjopen.bmj.com/site/about/guidelines.xhtml

(2–4) Gavin et al estimated point prevalence of minor and major depression was highest in the third month after delivery at 12.9%, although the confidence intervals were wide. (2) The results of our study suggest a peak in depression records and antidepressant treatment within 6 to 8 weeks after delivery, coinciding with the time of postnatal check-up consultations.

Our findings of increase in the use of symptoms codes as opposed to diagnostic codes for recording of depression reflect previous findings on recording of depression in primary care in general. (19) Rait et al suggest general practitioners' coding may be linked to the perceived severity of depression, with symptom codes being used for milder depression. Alternatively, this move towards recording of symptoms and less specific terms may be perceived as less stigmatising for individuals. (19)

Nearly 1 out 5 women in our study had a record suggestive of depression and/or SSRI treatment records prior to delivery. Of these women, 17% had additional records of depression and more than a quarter received SSRI treatment in the year after delivery. Prior depression has long been recognised as one of the strongest risk factors for depression in the year after delivery. (1–3,20) We also found that women who sought help early (before 42 days after delivery) were more likely to have had a prior record of depression or treatment. They might be better at recognising the symptoms earlier on than women without prior experience. Thus, a qualitative systematic review of help-seeking barriers by Dennis and Chung-Lee concluded that lack of knowledge about postpartum depression or the acceptance of myths was a significant help-seeking barrier and rendered mothers unable to recognise the symptoms of depression.(21)

Many women discontinue antidepressant treatment in pregnancy (22,23). A few studies suggest that these women are at higher risk of relapse (24) , but it is difficult to judge in observational settings and further research is needed to understand the role of antidepressant treatment in prevention of depression in the year after delivery.

Increased risk of postnatal depression among teenage mothers is well recognised with prevalence estimates as high as 26%. (25) Our study demonstrated that the level of recording of depressive diagnoses and symptoms continued to be higher for women right up to the age of 30, whereas no marked difference was found for women above the age of 30. Previous meta-analyses of postnatal depression have failed to recognise this 'L-shaped' difference in risk postnatal depression with age. (1,3) In contrast to our findings, a recent Canadian study on women aged 20 to 44 years based on the Canadian Community Health Survey suggests that there is a 'U-shaped' relationship with age and



For peer review only - http://bmjopen.bmj.com/site/about/guidelines.xhtml

postnatal depression. Thus, they found that the prevalence of depression in women who had recently delivered was significantly higher in women aged 40 to 44 years than in women aged 30 to 35 years (adjusted OR 3.72; 95% CI 2.15 to 6.41).(26)

There is some evidence that socioeconomic status is associated with prevalence of postnatal depression. (2,3,8,27) The results of a meta-regression analysis suggest that the prevalence of major depression is similar among socioeconomic status groups, but that minor depression may be more prevalent among lower socioeconomic status groups. (2) While we were unable to distinguish directly between diagnosis of major and minor depression we observed a clear gradient with increasing level of deprivation across all measures of depression and treatments. An even stronger socio-economic gradient in SSRI treatment was found among general population of adult women in UK. Hence, women from the most deprived areas were 64% more likely to have been initiated on SSRI treatment compared to women from the least deprived areas. (28)

Our study reflects women's primary care electronic health records. For women to have records of depression it requires that they have consulted their general practitioner. However, some women may be reluctant to seek help and unwilling to disclose or discuss their problem because of fear of stigma, negative perceptions of them as a mother or fear that their baby might be taken into care. (6,21,29) Investigators and clinicians should also be aware of the potential differences in the way women express postpartum depression and that it may differ for women of different educational backgrounds.(30) Likewise, some healthcare professionals may miss or misdiagnose postnatal depression in the period soon after birth (6) and estimates based on primary care health records may underestimate the 'true' prevalence of postnatal depression. Our study clearly shows that for many women depression and depressive symptoms were 'picked up' and treatment initiated at the time of the maternal check-up consultation in accordance to guidelines on antenatal and postnatal mental health care. (29) Yet, our results also revealed that depression is not limited to the immediate period after delivery and emphasises the need for health care professionals to be alert to signs and symptoms of depression throughout the first year after delivery. Indeed, a recent systematic review suggested that screening postpartum women for depression may reduce depressive symptoms in women with depression and reduce the prevalence of depression. (31)

## Conclusions

More than 1 in 10 women had electronic health records indicating depression or depressive symptoms within a year after delivery and more than 1 in 8 women received antidepressant treatment in this



For peer review only - http://bmjopen.bmj.com/site/about/guidelines.xhtml

period. Women aged below 30 and from the most deprived areas were at highest risk depression and most likely to receive antidepressant treatment.

## Author contributions

IP, TP, SH, KW and SK conceived the study, TP conducted the statistical analyses together with IP. IP and TP drafted the manuscript. All authors contributed to preparing the manuscript and have agreed to submit the final version of the manuscript. IP is the guarantor.

## Funding for this study

SH, IP and KW received funding from National Institute for Health Research (NIHR) school of primary care (grant 325). The views expressed in this publication are those of the author(s) and not necessarily those of the NHS, the National Institute for Health Research or the Department of Health. TP was funded by Academy of Finland (Finnish Centre of Excellence in Computational Inference Research Grant number 284642).

## Competing interest

None of the authors had competing interests.

## Data sharing

As the data for this study was bought under a licence no data are available for data sharing.

For peer review only

Figure legends

Figure 1. A. Numbers of records of depressive diagnoses and symptoms as well as treatment. B. Conditional frequency of records: given that one has the condition on the y-axis, what is the frequency of having the condition on x-axis. For example, the figure illustrates that 82% of those who had a diagnosis of depression also had a prescription of a SSRI. On the other hand, 31% of those who had a prescription of SSRI had a diagnosis of depression. D=Depression diagnosis, PND=Postnatal depression diagnosis, D/PND=either or both, D sym=Depression symptom, SSRI=SSRI prescription, NPT=Non-pharmacological treatment.

Figure 2. Cumulative incidences and smoothed hazards for the records. Six and eight weeks (6x7 and 8x7 days) are marked with a vertical grey line. Note the different y-axis scale for panels A and B. D=Depression, PND=Postnatal depression, SSRI=SSRI prescription, NPT=Non-pharmacological treatment.

Figure 3. Cumulative incidence of SSRI in three calendar periods. Six and eight weeks (6x7 and 8x7 days) are marked with a vertical grey line.

For peer review only



For peer review only - http://bmjopen.bmj.com/site/about/guidelines.xhtml

Table 1. Rates and Relative risk estimates of depression diagnosis, postnatal depression diagnosis and depression symptoms in the first year after delivery for 206,517 women who gave birth between 2000 and 2013. Adjusted by age group, calendar period, and Townsend deprivation

| | Depression diagnosis | | Postnatal depression diagnosis | | Depression symptom | |
|---|---|---|---|---|---|---|
| | Rate per 100 | Adjusted RR | Rate per 100 | Adjusted RR | Rate per 100 | Adjusted RR |
| Age group | | | | | | |
| 15-19 | 6.6 (6.1 to 7.1) | 1.64 (1.50 to 1.81) | 7.6 (7.1 to 8.1) | 1.92 (1.76 to 2.10) | 10.6 (10.0 to 11.2) | 2.10 (1.95 to 2.27) |
| 20-24 | 6.1 (5.9 to 6.4) | 1.59 (1.47 to 1.71) | 5.8 (5.5 to 6.0) | 1.49 (1.39 to 1.59) | 8.1 (7.7 to 8.4) | 1.63 (1.54 to 1.73) |
| 25-29 | 4.5 (4.3 to 4.7) | 1.22 (1.15 to 1.30) | 4.6 (4.4 to 4.8) | 1.21 (1.14 to 1.29) | 5.5 (5.3 to 5.7) | 1.18 (1.12 to 1.24) |
| 30-34 | 3.6 (3.4 to 3.7) | 1 | 3.8 (3.6 to 3.9) | 1 | 4.4 (4.3 to 4.6) | 1 |
| 35-39 | 3.5 (3.3 to 3.7) | 1.00 (0.93 to 1.06) | 3.5 (3.3 to 3.6) | 0.92 (0.86 to 0.98) | 4.3 (4.1 to 4.4) | 0.97 (0.92 to 1.02) |
| 40-49 | 3.1 (2.8 to 3.5) | 0.92 (0.81 to 1.03) | 3.2 (2.8 to 3.5) | 0.86 (0.77 to 0.97) | 4.7 (4.3 to 5.1) | 1.06 (0.96 to 1.17) |
| Calendar period | | | | | | |
| 2000-2004 | 5.7 (5.5 to 5.9) | 1 | 5.8 (5.6 to 6.0) | 1 | 4.4 (4.2 to 4.6) | 1 |
| 2005-2009 | 4.2 (4.0 to 4.3) | 0.71 (0.66 to 0.77) | 4.4 (4.2 to 4.5) | 0.73 (0.69 to 0.78) | 6.0 (5.8 to 6.2) | 1.31 (1.21 to 1.42) |
| 2010-2013 | 3.5 (3.3 to 3.6) | 0.58 (0.53 to 0.63) | 3.4 (3.3 to 3.5) | 0.56 (0.52 to 0.60) | 5.6 (5.4 to 5.8) | 1.21 (1.11 to 1.32) |
| Townsend deprivation index quintile | | | | | | |
| 1 | 3.2 (3.0 to 3.3) | 1 | 3.7 (3.5 to 3.9) | 1 | 4.1 (3.9 to 4.2) | 1 |
| 2 | 3.6 (3.4 to 3.8) | 1.14 (1.05 to 1.22) | 4.1 (3.9 to 4.2) | 1.09 (1.01 to 1.17) | 4.5 (4.3 to 4.7) | 1.07 (1.00 to 1.15) |
| 3 | 4.1 (3.9 to 4.3) | 1.26 (1.16 to 1.36) | 4.3 (4.1 to 4.5) | 1.12 (1.04 to 1.21) | 5.3 (5.1 to 5.5) | 1.19 (1.10 to 1.28) |
| 4 | 5.1 (4.9 to 5.3) | 1.51 (1.38 to 1.64) | 4.8 (4.6 to 5.0) | 1.20 (1.11 to 1.30) | 6.6 (6.4 to 6.9) | 1.42 (1.31 to 1.53) |
| 5 | 6.0 (5.7 to 6.3) | 1.69 (1.53 to 1.87) | 5.3 (5.0 to 5.5) | 1.26 (1.13 to 1.39) | 7.7 (7.4 to 8.0) | 1.56 (1.42 to 1.72) |

index. RR = relative risk.



For peer review only - http://bmjopen.bmj.com/site/about/guidelines.xhtml

Table 2. Rates and relative risk estimates of SSRI prescription and non-pharmacological treatment in the first year after delivery for 206,517 women who gave birth between 2000 and 2013. Adjusted by age group, calendar period, and Townsend deprivation index. RR = relative risk.

| | SSRI prescription | | Non-pharmacological treatment | |
|---|---|---|---|---|
| | Rate per 100 | Adjusted RR | Rate per 100 | Adjusted RR |
| Age group | | | | |
| 15-19 | 18.8 (18.0 to 19.5) | 1.78 (1.68 to 1.88) | 4.9 (4.5 to 5.4) | 1.55 (1.41 to 1.72) |
| 20-24 | 15.9 (15.5 to 16.4) | 1.54 (1.47 to 1.61) | 4.4 (4.1 to 4.6) | 1.38 (1.28 to 1.49) |
| 25-29 | 11.7 (11.5 to 12.0) | 1.18 (1.14 to 1.23) | 3.3 (3.2 to 3.5) | 1.11 (1.04 to 1.18) |
| 30-34 | 9.6 (9.4 to 9.8) | 1 | 2.9 (2.8 to 3.0) | 1 |
| 35-39 | 9.3 (9.1 to 9.6) | 0.99 (0.95 to 1.03) | 2.9 (2.7 to 3.0) | 0.99 (0.92 to 1.07) |
| 40-49 | 9.6 (9.0 to 10.1) | 1.01 (0.94 to 1.07) | 3.1 (2.7 to 3.4) | 1.05 (0.93 to 1.19) |
| Calendar period | | | | |
| 2000-2004 | 11.4 (11.1 to 11.7) | 1 | 2.4 (2.2 to 2.5) | 1 |
| 2005-2009 | 11.3 (11.1 to 11.6) | 0.97 (0.93 to 1.01) | 3.5 (3.3 to 3.6) | 1.43 (1.30 to 1.57) |
| 2010-2013 | 11.5 (11.2 to 11.7) | 0.96 (0.92 to 1.01) | 3.8 (3.6 to 3.9) | 1.54 (1.38 to 1.71) |
| Townsend deprivation index quantile | | | | |
| 1 | 8.9 (8.7 to 9.2) | 1 | 2.7 (2.5 to 2.8) | 1 |
| 2 | 10.0 (9.7 to 10.3) | 1.09 (1.04 to 1.14) | 3.0 (2.9 to 3.2) | 1.10 (1.00 to 1.20) |
| 3 | 11.3 (11.0 to 11.6) | 1.19 (1.14 to 1.25) | 3.3 (3.1 to 3.4) | 1.14 (1.04 to 1.25) |
| 4 | 13.1 (12.7 to 13.4) | 1.33 (1.25 to 1.40) | 3.8 (3.6 to 4.0) | 1.29 (1.17 to 1.42) |
| 5 | 15.2 (14.8 to 15.6) | 1.47 (1.38 to 1.57) | 4.2 (3.9 to 4.4) | 1.36 (1.22 to 1.52) |

For peer review only

## References

1. Beck CT. Predictors of postpartum depression: an update. Nurs Res. 2001 Oct;50(5):275–85.

2. Gavin NI, Gaynes BN, Lohr KN, Meltzer-Brody S, Gartlehner G, Swinson T. Perinatal depression: A systematic review of prevalence and incidence. Obstet Gynecol. 2005;106(5 I):1071–83.

3. O’hara MW, Swain AM. Rates and risk of postpartum depression—a meta-analysis. Int Rev Psychiatry. 1996 Jan;8(1):37–54.

4. Hahn-Holbrook J, Cornwell-Hinrichs T, Anaya I. Economic and Health Predictors of National Postpartum Depression Prevalence: A Systematic Review, Meta-analysis, and Meta-Regression of 291 Studies from 56 Countries. Front Psychiatry. 2017;8:248.

5. Bauer A, Parsonage M, Knapp M, Iemmi V, Adelaja B. Costs of perinatal mental health problems [Internet]. 2014 [cited 2015 Nov 6]. Available from: http://www.centreformentalhealth.org.uk/

6. Jones I, Shakespeare J. Postnatal depression. The BMJ. 2014 Aug 14;349:g4500.

7. Kingston D, Tough S, Whitfield H. Prenatal and postpartum maternal psychological distress and infant development: a systematic review. Child Psychiatry Hum Dev. 2012 Oct;43(5):683–714.

8. Robertson E, Grace S, Wallington T, Stewart DE. Antenatal risk factors for postpartum depression: a synthesis of recent literature. Gen Hosp Psychiatry. 2004 Aug;26(4):289–95.

9. Howard LM, Megnin-Viggars O, Symington I, Pilling S, On behalf of the Guideline Development Group. Antenatal and postnatal mental health: summary of updated NICE guidance. BMJ. 2014 Dec 18;349(dec18 5):g7394–g7394.

10. Stewart DE, Vigod S. Postpartum Depression. N Engl J Med. 2016 01;375(22):2177–86.

11. Chisholm J. The Read clinical classification. Br Med J. 1990 Apr 28;300(6732):1092.

12. Davé S, Petersen I. Creating medical and drug code lists to identify cases in primary care databases. Pharmacoepidemiol Drug Saf. 2009;18(8):704–7.

13. Townsend, Phillimore, Beattie A. Inequalities in health in the northern region. Newcastle upon Tyne: Northern Regional Health Authority and University of Bristol. University of Bristol; 1986.

14. Lis Y, Mann RD. The VAMP research multi-purpose database in the UK. J Clin Epidemiol. 1995 Mar;48(3):431–43.



For peer review only - http://bmjopen.bmj.com/site/about/guidelines.xhtml

15. Blak BT, Thompson M, Dattani H, Bourke A. Generalisability of The Health Improvement Network (THIN) database: demographics, chronic disease prevalence and mortality rates. Inform Prim Care. 2011;19(4):251–5.

16. Williams T, van Staa T, Puri S, Eaton S. Recent advances in the utility and use of the General Practice Research Database as an example of a UK Primary Care Data resource. Ther Adv Drug Saf. 2012 Apr;3(2):89–99.

17. Rasmussen CE, Williams CKI. Gaussian processes for machine learning. Cambridge, Mass: MIT Press; 2006. 248 p. (Adaptive computation and machine learning).

18. Falah-Hassani K, Shiri R, Dennis C-L. The prevalence of antenatal and postnatal co-morbid anxiety and depression: a meta-analysis. Psychol Med. 2017 Sep;47(12):2041–53.

19. Rait G, Walters K, Griffin M, Buszewicz M, Petersen I, Nazareth I. Recent trends in the incidence of recorded depression in primary care. Br J Psychiatry. 2009 Dec 1;195(6):520–4.

20. Milgrom J, Gemmill AW, Bilszta JL, Hayes B, Barnett B, Brooks J, et al. Antenatal risk factors for postnatal depression: a large prospective study. J Affect Disord. 2008 May;108(1-2):147–57.

21. Dennis C-L, Chung-Lee L. Postpartum Depression Help-Seeking Barriers and Maternal Treatment Preferences: A Qualitative Systematic Review. Birth. 2006 Dec;33(4):323–31.

22. Petersen I, Gilbert RE, Evans SJW, Man S-L, Nazareth I. Pregnancy as a major determinant for discontinuation of antidepressants: an analysis of data from The Health Improvement Network. J Clin Psychiatry. 2011 Jul;72(7):979–85.

23. Charlton R, Jordan S, Pierini A, Garne E, Neville A, Hansen A, et al. Selective serotonin reuptake inhibitor prescribing before, during and after pregnancy: a population-based study in six European regions. BJOG Int J Obstet Gynaecol. 2015 Jun;122(7):1010–20.

24. Cohen LS. Relapse of Major Depression During Pregnancy in Women Who Maintain or Discontinue Antidepressant Treatment. JAMA. 2006 Feb 1;295(5):499.

25. Troutman BR, Cutrona CE. Nonpsychotic postpartum depression among adolescent mothers. J Abnorm Psychol. 1990 Feb;99(1):69–78.

26. Muraca GM, Joseph KS. The association between maternal age and depression. J Obstet Gynaecol Can JOGC J Obstet Gynecol Can JOGC. 2014 Sep;36(9):803–10.

27. Hobfoll SE, Ritter C, Lavin J, Hulsizer MR, Cameron RP. Depression prevalence and incidence among inner-city pregnant and postpartum women. J Consult Clin Psychol. 1995 Jun;63(3):445–53.



For peer review only - http://bmjopen.bmj.com/site/about/guidelines.xhtml

28. McCrea RL, Sammon CJ, Nazareth I, Petersen I. Initiation and duration of selective serotonin reuptake inhibitor prescribing over time: UK cohort study. Br J Psychiatry. 2016 Nov 1;209(5):421–6.

29. National Institute for Health and Care Excellence (NICE). Antenatal and postnatal mental health: clinical management and service guidance [Internet]. 2014 Dec. Available from: http://guidance.nice.org.uk/cg185

30. Di Florio A, Putnam K, Altemus M, Apter G, Bergink V, Bilszta J, et al. The impact of education, country, race and ethnicity on the self-report of postpartum depression using the Edinburgh Postnatal Depression Scale. Psychol Med. 2017 Apr;47(5):787–99.

31. O'Connor E, Rossom RC, Henninger M, Groom HC, Burda BU. Primary Care Screening for and Treatment of Depression in Pregnant and Postpartum Women: Evidence Report and Systematic Review for the US Preventive Services Task Force. JAMA. 2016 Jan 26;315(4):388–406.

For peer review only

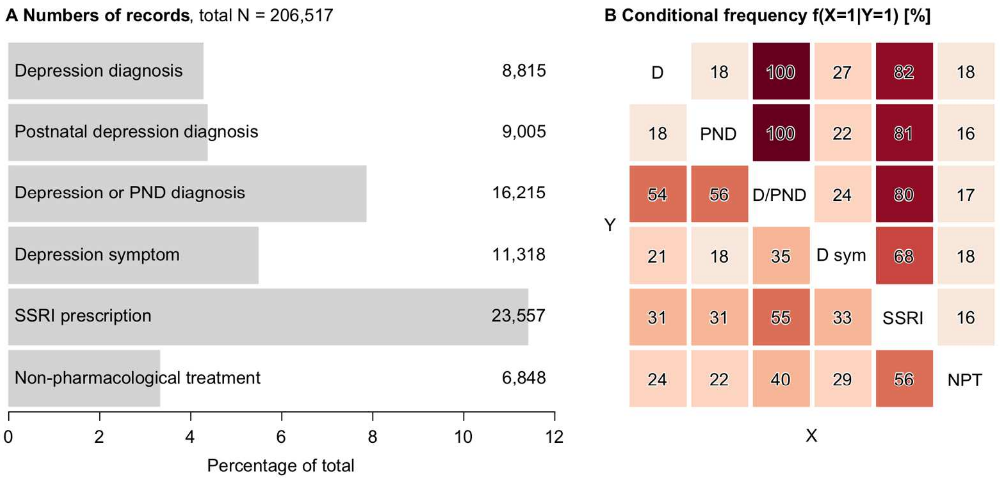


160x76mm (300 x 300 DPI)

er review only

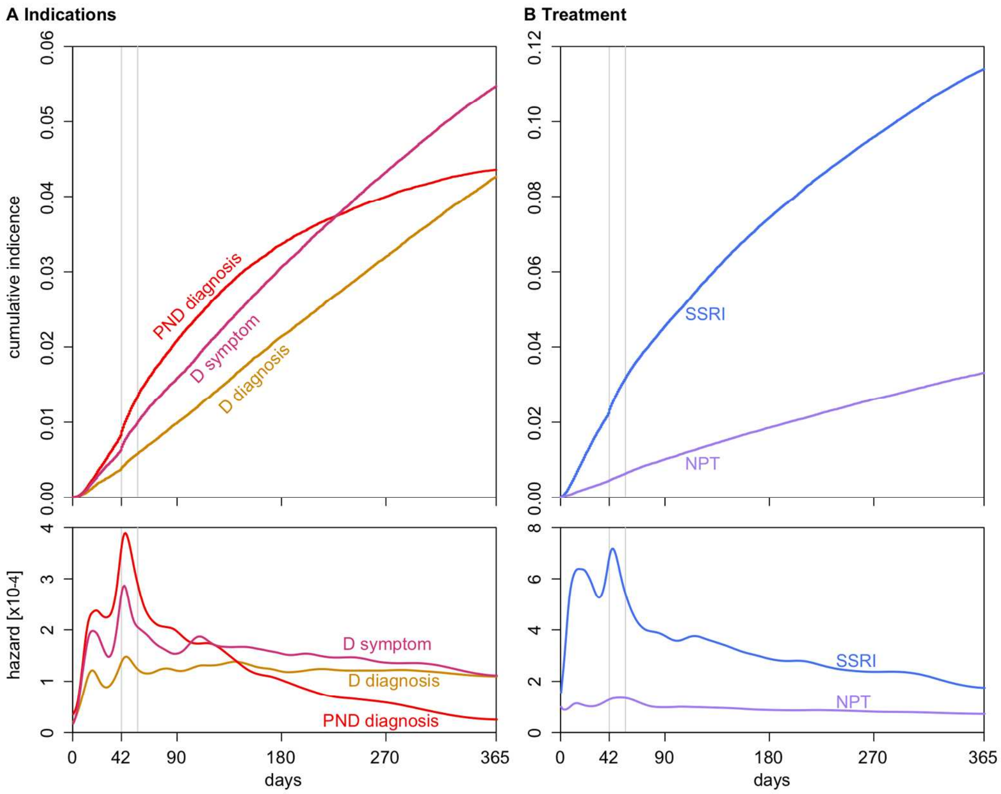


160x127mm (300 x 300 DPI)

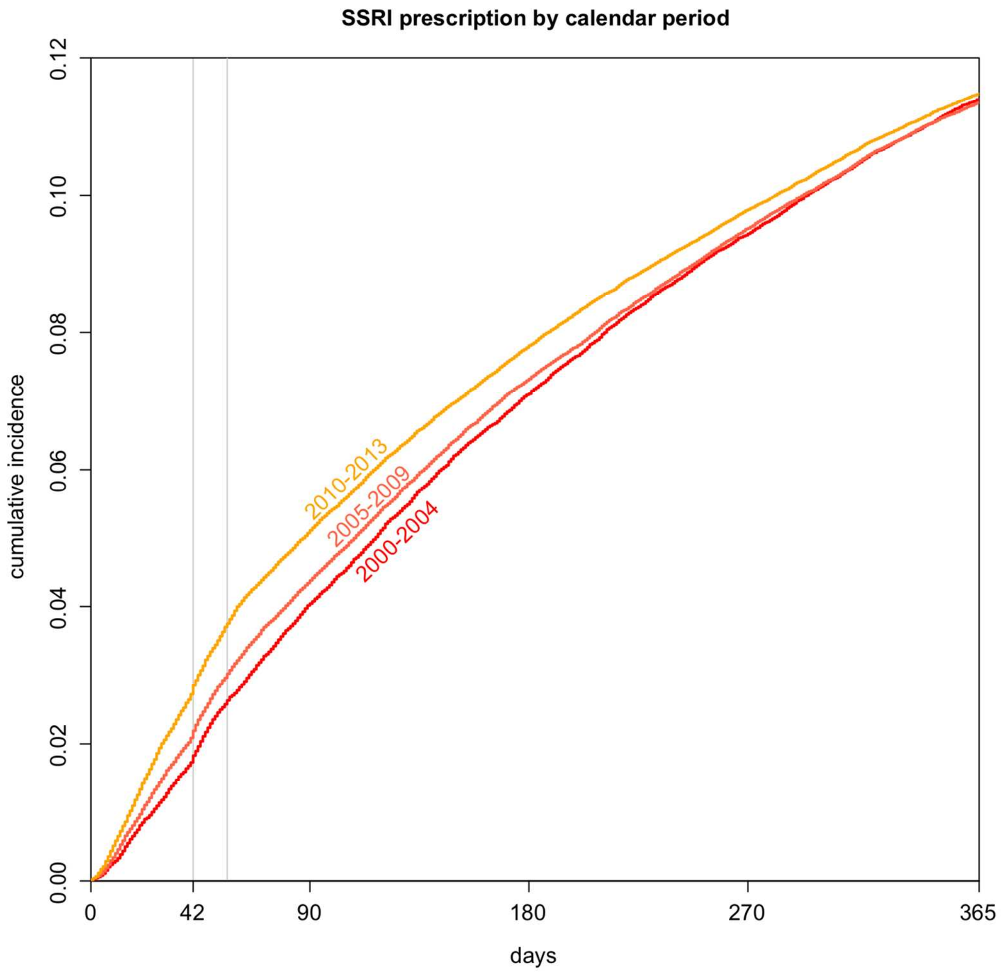


127x123mm (300 x 300 DPI)

STable 1. Rates and relative risk estimates of depression diagnosis, postnatal depression diagnosis and depression symptoms in the first year after delivery for 142,234 women who gave birth between 2000 and 2013 and had no prior record suggestive of depression. Adjusted by age group, calendar period, and Townsend deprivation index. RR = relative risk.

| | Depression diagnosis | | Postnatal depression diagnosis | | Depression symptom | |
|---|---|---|---|---|---|---|
| | Rate per 100 | Adjusted RR | Rate per 100 | Adjusted RR | Rate per 100 | Adjusted RR |
| Age group | | | | | | |
| 15-20 | 4.9 (4.4 to 5.3) | 2.74 (2.40 to 3.13) | 6.6 (6.0 to 7.1) | 2.88 (2.56 to 3.23) | 8.4 (7.8 to 9.0) | 3.35 (3.01 to 3.72) |
| 20-25 | 3.8 (3.5 to 4.0) | 2.21 (1.97 to 2.47) | 4.4 (4.1 to 4.7) | 1.98 (1.80 to 2.17) | 5.2 (4.9 to 5.6) | 2.13 (1.95 to 2.33) |
| 25-30 | 2.2 (2.0 to 2.4) | 1.33 (1.21 to 1.47) | 3.0 (2.8 to 3.2) | 1.33 (1.22 to 1.45) | 3.0 (2.8 to 3.1) | 1.26 (1.16 to 1.37) |
| 30-35 | 1.6 (1.5 to 1.7) | 1 | 2.3 (2.1 to 2.4) | 1 | 2.3 (2.1 to 2.4) | 1 |
| 35-40 | 1.4 (1.3 to 1.6) | 0.90 (0.81 to 1.01) | 2.0 (1.8 to 2.2) | 0.89 (0.80 to 0.98) | 2.0 (1.9 to 2.2) | 0.90 (0.82 to 0.99) |
| 40-50 | 1.2 (0.9 to 1.5) | 0.75 (0.59 to 0.95) | 1.8 (1.5 to 2.1) | 0.81 (0.67 to 0.99) | 1.8 (1.5 to 2.1) | 0.79 (0.64 to 0.96) |
| Calendar period | | | | | | |
| 2000-2004 | 3.0 (2.8 to 3.2) | 1 | 3.9 (3.7 to 4.1) | 1 | 2.6 (2.4 to 2.8) | 1 |
| 2005-2009 | 2.1 (2.0 to 2.2) | 0.67 (0.60 to 0.75) | 2.9 (2.7 to 3.0) | 0.70 (0.64 to 0.77) | 3.4 (3.3 to 3.6) | 1.27 (1.15 to 1.40) |
| 2010-2013 | 1.7 (1.6 to 1.8) | 0.53 (0.47 to 0.60) | 2.1 (2.0 to 2.3) | 0.52 (0.47 to 0.57) | 3.1 (2.9 to 3.2) | 1.13 (1.01 to 1.26) |
| Townsend deprivation index quantile | | | | | | |
| 1 | 1.6 (1.5 to 1.8) | 1 | 2.4 (2.2 to 2.5) | 1 | 2.3 (2.1 to 2.4) | 1 |
| 2 | 1.9 (1.7 to 2.0) | 1.11 (0.98 to 1.26) | 2.6 (2.4 to 2.8) | 1.06 (0.97 to 1.17) | 2.6 (2.4 to 2.7) | 1.05 (0.95 to 1.15) |
| 3 | 2.1 (2.0 to 2.3) | 1.19 (1.05 to 1.34) | 3.0 (2.8 to 3.2) | 1.17 (1.06 to 1.29) | 3.1 (2.9 to 3.3) | 1.18 (1.06 to 1.31) |
| 4 | 2.6 (2.4 to 2.8) | 1.34 (1.18 to 1.51) | 3.3 (3.1 to 3.5) | 1.16 (1.04 to 1.29) | 3.8 (3.6 to 4.0) | 1.28 (1.15 to 1.42) |
| 5 | 3.1 (2.8 to 3.3) | 1.42 (1.24 to 1.64) | 3.5 (3.2 to 3.7) | 1.10 (0.96 to 1.27) | 4.4 (4.1 to 4.7) | 1.32 (1.18 to 1.48) |

For peer review only

STable 2. Rates and relative risk estimates of SSRI prescription and non-pharmacological treatment in the first year after delivery for 142,234 women who gave birth between 2000 and 2013 and had no prior record suggestive of depression. Adjusted by age group, calendar period, and Townsend deprivation index. RR = relative risk.

| | SSRI prescription | | Non-pharmacological treatment | |
|---|---|---|---|---|
| | Rate per 100 | Adjusted RR | Rate per 100 | Adjusted RR |
| Age group | | | | |
| 15-20 | 14.2 (13.4 to 15.0) | 3.10 (2.85 to 3.37) | 3.4 (3.0 to 3.8) | 2.48 (2.12 to 2.90) |
| 20-25 | 9.9 (9.4 to 10.3) | 2.20 (2.06 to 2.36) | 2.2 (2.0 to 2.4) | 1.60 (1.40 to 1.84) |
| 25-30 | 5.8 (5.5 to 6.0) | 1.32 (1.24 to 1.41) | 1.6 (1.5 to 1.8) | 1.21 (1.08 to 1.36) |
| 30-35 | 4.3 (4.1 to 4.5) | 1 | 1.3 (1.2 to 1.4) | 1 |
| 35-40 | 3.7 (3.5 to 3.9) | 0.86 (0.80 to 0.93) | 1.3 (1.1 to 1.4) | 0.94 (0.83 to 1.07) |
| 40-50 | 3.1 (2.7 to 3.5) | 0.73 (0.63 to 0.84) | 1.2 (0.9 to 1.4) | 0.87 (0.69 to 1.11) |
| | | | | |
| Calendar period | | | | |
| 2000-2004 | 6.5 (6.3 to 6.8) | 1 | 1.2 (1.1 to 1.3) | 1 |
| 2005-2009 | 5.8 (5.7 to 6.0) | 0.86 (0.81 to 0.91) | 1.8 (1.7 to 1.9) | 1.42 (1.25 to 1.62) |
| 2010-2013 | 5.1 (4.9 to 5.3) | 0.75 (0.70 to 0.80) | 1.7 (1.6 to 1.9) | 1.40 (1.22 to 1.61) |
| | | | | |
| Townsend deprivation index quantile | | | | |
| 1 | 4.5 (4.3 to 4.7) | 1 | 1.4 (1.2 to 1.5) | 1 |
| 2 | 4.9 (4.7 to 5.2) | 1.05 (0.98 to 1.13) | 1.5 (1.4 to 1.6) | 1.06 (0.92 to 1.23) |
| 3 | 5.9 (5.6 to 6.1) | 1.15 (1.07 to 1.24) | 1.6 (1.5 to 1.8) | 1.08 (0.93 to 1.24) |
| 4 | 6.7 (6.4 to 7.0) | 1.18 (1.09 to 1.28) | 1.8 (1.7 to 2.0) | 1.12 (0.97 to 1.30) |
| 5 | 8.0 (7.6 to 8.4) | 1.26 (1.13 to 1.40) | 2.0 (1.8 to 2.2) | 1.12 (0.94 to 1.32) |

For peer review only

STable 3. Rates and relative risk estimates of depression diagnosis, postnatal depression diagnosis and depression symptoms in the first year after delivery for 64,283 women who gave birth between 2000 and 2013 and had a prior record suggestive of depression. Adjusted by age group, calendar period, and Townsend deprivation index. RR = relative risk.

| | Depression diagnosis | | Postnatal depression diagnosis | | Depression symptom | |
|---|---|---|---|---|---|---|
| | Rate per 100 | Adjusted RR | Rate per 100 | Adjusted RR | Rate per 100 | Adjusted RR |
| Age group | | | | | | |
| 15-20 | 12.6 (11.3 to 14.0) | 1.48 (1.31 to 1.67) | 11.1 (9.8 to 12.4) | 1.50 (1.32 to 1.71) | 18.3 (16.7 to 19.9) | 1.76 (1.58 to 1.95) |
| 20-25 | 10.6 (10.0 to 11.2) | 1.24 (1.15 to 1.35) | 8.2 (7.7 to 8.7) | 1.11 (1.02 to 1.21) | 13.3 (12.7 to 14.0) | 1.29 (1.22 to 1.38) |
| 25-30 | 9.2 (8.7 to 9.6) | 1.10 (1.03 to 1.17) | 7.9 (7.5 to 8.3) | 1.07 (0.99 to 1.15) | 10.6 (10.1 to 11.1) | 1.06 (0.99 to 1.12) |
| 30-35 | 8.3 (7.9 to 8.7) | 1 | 7.6 (7.2 to 8.0) | 1 | 9.8 (9.3 to 10.2) | 1 |
| 35-40 | 8.0 (7.6 to 8.5) | 0.98 (0.91 to 1.06) | 6.8 (6.3 to 7.2) | 0.90 (0.82 to 0.97) | 9.2 (8.7 to 9.7) | 0.95 (0.88 to 1.01) |
| 40-50 | 6.7 (5.8 to 7.5) | 0.84 (0.73 to 0.97) | 5.7 (4.9 to 6.5) | 0.78 (0.67 to 0.89) | 9.9 (8.9 to 11.0) | 1.02 (0.91 to 1.14) |
| Calendar period | | | | | | |
| 2000-2004 | 12.7 (12.1 to 13.2) | 1 | 10.8 (10.2 to 11.3) | 1 | 9.1 (8.7 to 9.6) | 1 |
| 2005-2009 | 8.8 (8.4 to 9.1) | 0.68 (0.63 to 0.74) | 7.7 (7.3 to 8.0) | 0.71 (0.66 to 0.77) | 11.7 (11.3 to 12.1) | 1.26 (1.16 to 1.38) |
| 2010-2013 | 7.0 (6.7 to 7.3) | 0.54 (0.50 to 0.59) | 5.9 (5.6 to 6.2) | 0.54 (0.50 to 0.59) | 10.6 (10.3 to 11.0) | 1.14 (1.03 to 1.25) |
| Townsend deprivation index quantile | | | | | | |
| 1 | 7.6 (7.2 to 8.1) | 1 | 7.6 (7.1 to 8.0) | 1 | 9.2 (8.7 to 9.7) | 1 |
| 2 | 8.1 (7.6 to 8.6) | 1.06 (0.98 to 1.16) | 7.8 (7.3 to 8.2) | 1.04 (0.95 to 1.13) | 9.5 (9.0 to 10.1) | 1.01 (0.93 to 1.11) |
| 3 | 8.5 (8.0 to 8.9) | 1.10 (1.01 to 1.20) | 7.1 (6.6 to 7.5) | 0.93 (0.85 to 1.03) | 10.0 (9.5 to 10.5) | 1.04 (0.95 to 1.13) |
| 4 | 9.7 (9.2 to 10.2) | 1.25 (1.15 to 1.36) | 7.7 (7.2 to 8.1) | 1.00 (0.92 to 1.09) | 11.9 (11.4 to 12.4) | 1.21 (1.11 to 1.32) |
| 5 | 10.6 (10.0 to 11.1) | 1.33 (1.20 to 1.47) | 8.2 (7.7 to 8.7) | 1.05 (0.94 to 1.16) | 13.0 (12.4 to 13.6) | 1.29 (1.16 to 1.43) |

For peer review only

STable 4. Rates and relative risk estimates of SSRI prescription and non-pharmacological treatment in the first year after delivery for 64,283 women who gave birth between 2000 and 2013 and had a prior record suggestive of depression. Adjusted by age group, calendar period, and Townsend deprivation index. RR = relative risk.

| | SSRI prescription | | Non-pharmacological treatment | |
|---|---|---|---|---|
| | Rate per 100 | Adjusted RR | Rate per 100 | Adjusted RR |
| Age group | | | | |
| 15-20 | 34.3 (32.4 to 36.3) | 1.47 (1.38 to 1.57) | 10.1 (8.8 to 11.3) | 1.42 (1.25 to 1.62) |
| 20-25 | 27.3 (26.4 to 28.1) | 1.18 (1.13 to 1.23) | 8.3 (7.8 to 8.9) | 1.18 (1.09 to 1.29) |
| 25-30 | 23.9 (23.2 to 24.5) | 1.04 (1.01 to 1.08) | 6.8 (6.4 to 7.2) | 0.99 (0.91 to 1.06) |
| 30-35 | 22.6 (22.0 to 23.2) | 1 | 6.8 (6.4 to 7.1) | 1 |
| 35-40 | 22.0 (21.2 to 22.7) | 0.98 (0.94 to 1.02) | 6.5 (6.0 to 6.9) | 0.95 (0.87 to 1.04) |
| 40-50 | 21.2 (19.8 to 22.6) | 0.94 (0.88 to 1.01) | 6.5 (5.7 to 7.4) | 0.95 (0.83 to 1.09) |
| Calendar period | | | | |
| 2000-2004 | 24.0 (23.3 to 24.8) | 1 | 5.4 (5.0 to 5.8) | 1 |
| 2005-2009 | 23.6 (23.1 to 24.1) | 0.97 (0.93 to 1.02) | 7.3 (7.0 to 7.6) | 1.34 (1.21 to 1.49) |
| 2010-2013 | 24.1 (23.6 to 24.6) | 0.99 (0.95 to 1.03) | 7.8 (7.5 to 8.1) | 1.43 (1.27 to 1.61) |
| Townsend deprivation index quantile | | | | |
| 1 | 21.7 (21.0 to 22.4) | 1 | 6.5 (6.0 to 6.9) | 1 |
| 2 | 22.7 (22.0 to 23.5) | 1.04 (0.99 to 1.09) | 6.8 (6.4 to 7.3) | 1.04 (0.93 to 1.16) |
| 3 | 23.2 (22.5 to 23.9) | 1.04 (0.99 to 1.10) | 6.9 (6.5 to 7.3) | 1.02 (0.92 to 1.14) |
| 4 | 25.0 (24.3 to 25.7) | 1.11 (1.06 to 1.16) | 7.5 (7.1 to 8.0) | 1.10 (0.99 to 1.23) |
| 5 | 26.6 (25.8 to 27.4) | 1.16 (1.10 to 1.22) | 7.6 (7.2 to 8.1) | 1.10 (0.97 to 1.25) |

For peer review only

STable 5. Rates of early recording (recording date within 42 days of delivery date) and odds ratio estimates of early vs. late recording (at or after 42 days) for depression diagnosis, postnatal depression diagnosis and depression symptoms in the first year after delivery for women who gave birth between 2000 and 2013. The values are calculated in datasets that only contain the women with the corresponding record within one year after delivery (number of women are given in the headers). Adjusted by age group, calendar period, Townsend deprivation index, and a prior record suggestive of depression. OR = odds ratio.

| | Depression diagnosis (N=8,815) | | Postnatal depression diagnosis (N=9,005) | | Depression symptom (N=11,318) | |
|---|---|---|---|---|---|---|
| | Rate per 100 | Adjusted OR | Rate per 100 | Adjusted OR | Rate per 100 | Adjusted OR |
| Age group | | | | | | |
| 15-20 | 8.6 (6.4 to 10.7) | 1.23 (0.89 to 1.67) | 23.3 (20.3 to 26.3) | 1.43 (1.17 to 1.75) | 12.5 (10.5 to 14.5) | 1.23 (0.98 to 1.53) |
| 20-25 | 8.1 (6.8 to 9.4) | 1.00 (0.80 to 1.26) | 19.2 (17.3 to 21.1) | 1.04 (0.88 to 1.22) | 12.9 (11.6 to 14.3) | 1.19 (1.00 to 1.41) |
| 25-30 | 8.6 (7.4 to 9.7) | 1.00 (0.82 to 1.24) | 17.6 (16.0 to 19.1) | 0.91 (0.79 to 1.06) | 10.8 (9.6 to 11.9) | 0.96 (0.81 to 1.14) |
| 30-35 | 8.5 (7.4 to 9.7) | 1 | 18.7 (17.2 to 20.3) | 1 | 10.9 (9.7 to 12.0) | 1 |
| 35-40 | 9.8 (8.3 to 11.4) | 1.14 (0.91 to 1.43) | 18.2 (16.2 to 20.2) | 0.95 (0.80 to 1.12) | 10.4 (9.0 to 11.8) | 0.95 (0.78 to 1.16) |
| 40-50 | 10.0 (6.6 to 13.4) | 1.12 (0.73 to 1.66) | 19.5 (15.0 to 23.9) | 1.03 (0.76 to 1.39) | 12.5 (9.4 to 15.6) | 1.08 (0.79 to 1.46) |
| Calendar period | | | | | | |
| 2000-2004 | 8.6 (7.5 to 9.6) | 1 | 17.6 (16.2 to 19.0) | 1 | 8.4 (7.2 to 9.5) | 1 |
| 2005-2009 | 8.4 (7.4 to 9.3) | 0.94 (0.79 to 1.13) | 18.0 (16.7 to 19.2) | 0.99 (0.87 to 1.13) | 10.9 (10.1 to 11.8) | 1.31 (1.10 to 1.57) |
| 2010-2013 | 9.4 (8.3 to 10.5) | 1.06 (0.87 to 1.28) | 21.6 (20.0 to 23.2) | 1.24 (1.08 to 1.42) | 13.6 (12.6 to 14.6) | 1.65 (1.38 to 1.97) |
| Townsend deprivation index quantile | | | | | | |
| 1 | 8.7 (7.2 to 10.1) | 1 | 19.3 (17.5 to 21.1) | 1 | 9.5 (8.2 to 10.8) | 1 |
| 2 | 9.0 (7.6 to 10.5) | 1.04 (0.81 to 1.34) | 16.2 (14.5 to 18.0) | 0.79 (0.66 to 0.94) | 10.6 (9.2 to 12.0) | 1.10 (0.89 to 1.36) |
| 3 | 8.3 (7.0 to 9.6) | 0.94 (0.74 to 1.21) | 18.2 (16.5 to 19.9) | 0.91 (0.77 to 1.07) | 12.5 (11.2 to 13.8) | 1.30 (1.07 to 1.59) |
| 4 | 8.7 (7.5 to 9.9) | 0.97 (0.77 to 1.24) | 21.2 (19.4 to 23.0) | 1.07 (0.91 to 1.26) | 11.9 (10.7 to 13.1) | 1.20 (0.99 to 1.46) |
| 5 | 9.0 (7.7 to 10.3) | 0.99 (0.78 to 1.27) | 19.0 (17.1 to 20.9) | 0.89 (0.75 to 1.06) | 12.0 (10.7 to 13.3) | 1.19 (0.98 to 1.46) |
| Any prior record suggestive of depression | | | | | | |
| No | 4.8 (4.0 to 5.5) | 1 | 15.4 (14.2 to 16.5) | 1 | 8.9 (8.0 to 9.7) | 1 |
| Yes | 10.8 (10.0 to 11.6) | 2.43 (2.02 to 2.94) | 21.8 (20.6 to 22.9) | 1.58 (1.41 to 1.77) | 13.0 (12.2 to 13.8) | 1.55 (1.37 to 1.76) |

For peer review only

STable 6. Rates of early recording (recording date within 42 days of delivery date) and odds ratio estimates of early vs. late recording (at or after 42 days) for SSRI prescription and non-pharmacological treatment in the first year after delivery for women who gave birth between 2000 and 2013. The values are calculated in datasets that only contain the women with the corresponding record within one year after delivery (number of women are given in the headers). Adjusted by age group, calendar period, Townsend deprivation index, and a prior record suggestive of depression. OR = odds ratio.

| | SSRI prescription (N=23,557) | | Non-pharmacological treatment (N=6,848) | |
|---|---|---|---|---|
| | Rate per 100 | Adjusted OR | Rate per 100 | Adjusted OR |
| Age group | | | | |
| 15-20 | 17.4 (15.7 to 19.1) | 0.99 (0.86 to 1.14) | 12.5 (9.6 to 15.4) | 1.01 (0.74 to 1.36) |
| 20-25 | 17.6 (16.5 to 18.7) | 0.83 (0.75 to 0.92) | 11.7 (9.9 to 13.4) | 0.82 (0.66 to 1.03) |
| 25-30 | 18.6 (17.7 to 19.6) | 0.85 (0.77 to 0.93) | 13.4 (11.7 to 15.0) | 0.96 (0.79 to 1.17) |
| 30-35 | 21.0 (20.0 to 22.0) | 1 | 13.8 (12.2 to 15.3) | 1 |
| 35-40 | 21.0 (19.7 to 22.3) | 0.96 (0.87 to 1.06) | 14.3 (12.3 to 16.3) | 1.03 (0.84 to 1.27) |
| 40-50 | 24.3 (21.5 to 27.1) | 1.07 (0.90 to 1.26) | 16.0 (11.8 to 20.2) | 1.13 (0.79 to 1.57) |
| Calendar period | | | | |
| 2000-2004 | 15.2 (14.2 to 16.1) | 1 | 12.0 (10.1 to 13.8) | 1 |
| 2005-2009 | 18.4 (17.6 to 19.2) | 1.20 (1.09 to 1.31) | 11.4 (10.3 to 12.6) | 0.95 (0.77 to 1.17) |
| 2010-2013 | 23.7 (22.8 to 24.6) | 1.59 (1.46 to 1.74) | 16.0 (14.6 to 17.4) | 1.36 (1.11 to 1.68) |
| Townsend deprivation index quantile | | | | |
| 1 | 18.7 (17.6 to 19.9) | 1 | 13.3 (11.5 to 15.2) | 1 |
| 2 | 19.1 (17.9 to 20.3) | 1.00 (0.90 to 1.12) | 12.8 (10.9 to 14.6) | 0.94 (0.74 to 1.19) |
| 3 | 19.3 (18.2 to 20.4) | 1.02 (0.92 to 1.14) | 14.0 (12.2 to 15.8) | 1.05 (0.84 to 1.31) |
| 4 | 20.4 (19.4 to 21.5) | 1.09 (0.98 to 1.21) | 13.4 (11.7 to 15.1) | 0.98 (0.79 to 1.23) |
| 5 | 20.0 (18.9 to 21.2) | 1.06 (0.95 to 1.18) | 13.3 (11.4 to 15.1) | 0.97 (0.77 to 1.23) |
| Any prior record suggestive of depression | | | | |
| No | 9.6 (8.9 to 10.2) | 1 | 8.8 (7.6 to 9.9) | 1 |
| Yes | 24.9 (24.2 to 25.6) | 3.02 (2.78 to 3.29) | 15.7 (14.7 to 16.8) | 1.91 (1.62 to 2.27) |

For peer review only

**STROBE 2007 (v4) Statement—Checklist of items that should be included in reports of *cohort studies***

| Section/Topic | Item # | Recommendation | Reported on page # |
|---|---|---|---|
| **Title and abstract** | 1 | (*a*) Indicate the study's design with a commonly used term in the title or the abstract | 1 |
| | | (*b*) Provide in the abstract an informative and balanced summary of what was done and what was found | 2 |
| **Introduction** | | | |
| Background/rationale | 2 | Explain the scientific background and rationale for the investigation being reported | 4 |
| Objectives | 3 | State specific objectives, including any prespecified hypotheses | 4 |
| **Methods** | | | |
| Study design | 4 | Present key elements of study design early in the paper | 5 |
| Setting | 5 | Describe the setting, locations, and relevant dates, including periods of recruitment, exposure, follow-up, and data collection | 5 |
| Participants | 6 | (*a*) Give the eligibility criteria, and the sources and methods of selection of participants. Describe methods of follow-up | 5 |
| | | (*b*) For matched studies, give matching criteria and number of exposed and unexposed | N/A |
| Variables | 7 | Clearly define all outcomes, exposures, predictors, potential confounders, and effect modifiers. Give diagnostic criteria, if applicable | 5 |
| Data sources/ measurement | 8* | For each variable of interest, give sources of data and details of methods of assessment (measurement). Describe comparability of assessment methods if there is more than one group | 5 |
| Bias | 9 | Describe any efforts to address potential sources of bias | 6 |
| Study size | 10 | Explain how the study size was arrived at | 5 |
| Quantitative variables | 11 | Explain how quantitative variables were handled in the analyses. If applicable, describe which groupings were chosen and why | 6 |
| Statistical methods | 12 | (*a*) Describe all statistical methods, including those used to control for confounding | 6 |
| | | (*b*) Describe any methods used to examine subgroups and interactions | 6 |
| | | (*c*) Explain how missing data were addressed | 6 |
| | | (*d*) If applicable, explain how loss to follow-up was addressed | 6 |
| | | (*e*) Describe any sensitivity analyses | 6 |
| **Results** | | | |

For peer review only

| | | | |
|---|---|---|---|
| Participants | 13* | (a) Report numbers of individuals at each stage of study—eg numbers potentially eligible, examined for eligibility, confirmed eligible, included in the study, completing follow-up, and analysed | 7 |
| | | (b) Give reasons for non-participation at each stage | N/A |
| | | (c) Consider use of a flow diagram | N/A |
| Descriptive data | 14* | (a) Give characteristics of study participants (eg demographic, clinical, social) and information on exposures and potential confounders | 7 |
| | | (b) Indicate number of participants with missing data for each variable of interest | N/A |
| | | (c) Summarise follow-up time (eg, average and total amount) | 7 |
| Outcome data | 15* | Report numbers of outcome events or summary measures over time | 7 |
| Main results | 16 | (*a*) Give unadjusted estimates and, if applicable, confounder-adjusted estimates and their precision (eg, 95% confidence interval). Make clear which confounders were adjusted for and why they were included | 8 |
| | | (*b*) Report category boundaries when continuous variables were categorized | 8 |
| | | (*c*) If relevant, consider translating estimates of relative risk into absolute risk for a meaningful time period | 8 |
| Other analyses | 17 | Report other analyses done—eg analyses of subgroups and interactions, and sensitivity analyses | 8-9 |
| **Discussion** | | | |
| Key results | 18 | Summarise key results with reference to study objectives | 9 |
| **Limitations** | | | |
| Interpretation | 20 | Give a cautious overall interpretation of results considering objectives, limitations, multiplicity of analyses, results from similar studies, and other relevant evidence | 10 |
| Generalisability | 21 | Discuss the generalisability (external validity) of the study results | 10 |
| **Other information** | | | |
| Funding | 22 | Give the source of funding and the role of the funders for the present study and, if applicable, for the original study on which the present article is based | 12 |

*Give information separately for cases and controls in case-control studies and, if applicable, for exposed and unexposed groups in cohort and cross-sectional studies.

**Note:** An Explanation and Elaboration article discusses each checklist item and gives methodological background and published examples of transparent reporting. The STROBE checklist is best used in conjunction with this article (freely available on the Web sites of PLoS Medicine at http://www.plosmedicine.org/, Annals of Internal Medicine at http://www.annals.org/, and Epidemiology at http://www.epidem.com/). Information on the STROBE Initiative is available at www.strobe-statement.org.

For peer review only
